# Selective Electrocatalytic Oxidation of Biomass‐Derived 5‐Hydroxymethylfurfural to 2,5‐Diformylfuran: from Mechanistic Investigations to Catalyst Recovery

**DOI:** 10.1002/cssc.202000453

**Published:** 2020-06-02

**Authors:** Peter Kisszekelyi, Rifan Hardian, Hakkim Vovusha, Binglin Chen, Xianhai Zeng, Udo Schwingenschlögl, Jozsef Kupai, Gyorgy Szekely

**Affiliations:** ^1^ Department of Organic Chemistry and Technology Budapest University of Technology and Economics Szent Gellert ter 4 Budapest 1111 Hungary; ^2^ Advanced Membranes and Porous Materials Center Physical Science and Engineering Division (PSE) King Abdullah University of Science and Technology Thuwal 23955-6900 Saudi Arabia; ^3^ Physical Science and Engineering Division (PSE) King Abdullah University of Science and Technology Thuwal 23955-6900 Saudi Arabia; ^4^ College of Energy Xiamen University Xiamen 361102 P. R. China; ^5^ Fujian Engineering and Research Center of Clean and High-Valued Technologies for Biomass, Xiamen Key Laboratory of High-Valued Utilization of Biomass Xiamen University Xiamen 361102 P. R. China; ^6^ Department of Chemical Engineering and Analytical Science The University of Manchester The Mill, Sackville Street Manchester M1 3BB United Kingdom

**Keywords:** biomass, diformylfuran, electrochemistry, hydroxymethylfurfural, organic solvent nanofiltration

## Abstract

The catalytic transformation of bio‐derived compounds, specifically 5‐hydroxymethylfurfural (HMF), into value‐added chemicals may provide sustainable alternatives to crude oil and natural gas‐based products. HMF can be obtained from fructose and successfully converted to 2,5‐diformylfuran (DFF) by an environmentally friendly organic electrosynthesis performed in an ElectraSyn reactor, using cost‐effective and sustainable graphite (anode) and stainless‐steel (cathode) electrodes in an undivided cell, eliminating the need for conventional precious metal electrodes. In this work, the electrocatalysis of HMF is performed by using green solvents such as acetonitrile, γ‐valerolactone, as well as PolarClean, which is used in electrocatalysis for the first time. The reaction parameters and the synergistic effects of the TEMPO catalyst and 2,6‐lutidine base are explored both experimentally and through computation modeling. The molecular design and synthesis of a size‐enlarged *C*_3_‐symmetric tris‐TEMPO catalyst are also performed to facilitate a sustainable reaction work‐up through nanofiltration. The obtained performance is then compared with those obtained by heterogeneous TEMPO alternatives recovered by using an external magnetic field and microfiltration. Results show that this new method of electrocatalytic oxidation of HMF to DFF can be achieved with excellent selectivity, good yield, and excellent catalyst recovery.

## Graphical abstract

**Up the tempo**: An efficient TEMPO‐based electrocatalytic transformation of biomass‐based C_6_‐platform chemical 5‐hydroxymethylfurfural (HMF) to 2,5‐diformylfuran (DFF) is reported. The molecular design and comparison of homogeneous (native, size‐enlarged) and solid‐supported (SiliaCAT, TurboBeads) TEMPOs is discussed for the oxidation in the ElectraSyn reactor with cost‐effective electrodes and green solvents. Computational modeling reveals the synergistic effect of the catalyst/base system and facilitated the catalyst design.

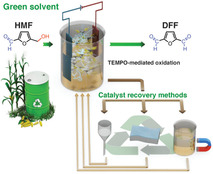

## Introduction

Owing to the growing awareness of the inconvenient utilization of diminishing fossil resources, the fast‐rising levels of carbon dioxide emissions, and the ever‐increasing demand in energy, biomass‐based chemical platforms have gained much interest. In particular, the utilization of agricultural wastes shows great promise. Catalytic transformation of lignocellulosic biomass into value‐added chemical compounds could provide a renewable, carbon‐neutral feedstock platform that might be a sustainable alternative to the crude oil and natural gas based bulk chemical industry.^1^

**Figure 1:**
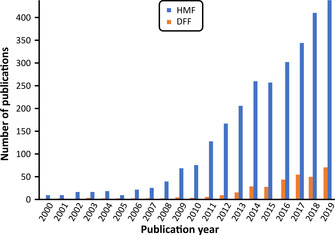
Annual number of publications related to HMF and DFF. Search engine: Web of Science; keywords: 5‐hydroxymethylfurfural and 2,5‐diformylfuran; 16.10.2019.

Within the furan family, 5‐hydroxymethylfurfural (HMF) is a potential C_6_ carbohydrate‐based building block and is attracting a lot of interest (Figure 1).^2^ Being accessible by the acid‐catalyzed dehydration of hexoses, HMF is also a naturally occurring substance, and its market, which is increasing rapidly worldwide, is expected to reach 61 million USD in 2024.^3^

**Scheme 1:**
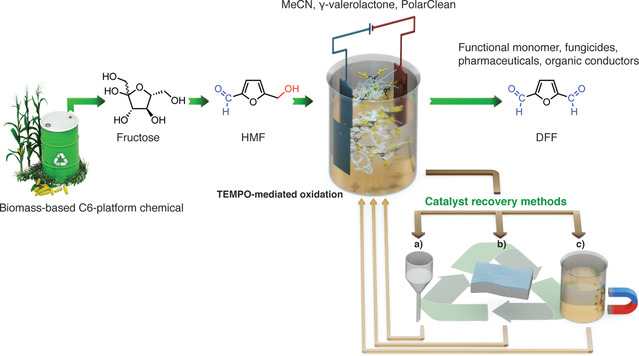
Schematic representation of recyclable TEMPO‐mediated electrochemical oxidation of biomass‐based HMF. Catalyst recovered by (a) microfiltration, (b) nanofiltration, and (c) magnetic separation.

As a platform chemical, HMF can be transformed into several high‐value derivatives.^4^ 2,5‐Furandicarboxylic acid and its dimethyl ester are both promising monomers to produce furan‐based polyesters as an alternative to the petrochemical‐based polyethylene terephthalate (PET).^5^ The hydrogenated diol derivatives, 2,5‐bis(hydroxymethyl)furan and 2,5‐bis(hydroxymethyl)tetrahydrofuran, are both valuable polymer‐building blocks for the synthesis of polyurethanes, aromatic resins, and polyesters.^6^ 2,5‐Diformylfuran (DFF), which contains two reactive aldehyde groups, is a particularly useful derivative of HMF (Scheme 1) with potential applications as an intermediate for pharmaceuticals,^7^ functional polymers,^8^ fungicides,^9^ macrocyclic ligands,^10^ organic conductors,^11^ and as a crosslinking agent of poly(vinyl alcohol) for battery separations.^12^ This bis(aldehyde) is usually synthesized by oxidation of the primary hydroxyl group of HMF, and owing to the reactive nature of the CHO, selectivity plays a key role in the efficiency of the production. Therefore, several homogeneous and heterogeneous metal‐promoted (vanadium, manganese, and precious metals) oxidation procedures have been suggested for the synthesis of DFF.^13^

Electrochemical platforms have the potential to provide an environmentally friendly solution for the oxidation of sensitive compounds. Because of the multitude of adjustable reaction parameters such as electrode materials, electrolyte, solvent, current strength, potential, the selectivity of the reaction can be fine‐tuned. Furthermore, by using renewable energy sources and recyclable catalyst/electrolyte systems, electroorganic methodologies could offer sustainable synthetic processes.^14^

The scientific literature on the electrocatalytic oxidation of HMF to DFF is scarce (Table 1). Skowroński et al. performed a selective electrooxidation with a Pt anode in a biphasic system, using acetic acid or inorganic salts as supporting electrolytes;^15^ Cao et al. utilized a PtRu alloy to exploit the simultaneous generation of electricity on the cathode in a membrane‐electrode reactor.^16^

**Table 1:** Comparison of selective electrocatalytic oxidations of HMF to DFF.

Anode	Cathode	Catalyst	Solvent	DFF selectivity [%]	Yield [%]	Recycling	Ref.	
Pt	Pt	–	CH_2_Cl_2_/aq. electrolyte	>99	68^[a]^	–	15	
PtRu	Pt	–	H_2_SO_4_ (aq)	89	40^[b]^	–	16	
Pt	Pt	4‐AcNH‐TEMPO, KI	CH_2_Cl_2_/aq. electrolyte	69	58^[a]^	electrolyte	21	
**graphite**	**stainless steel**	**recyclable TEMPO**	**MeCN, GVL, or PolarClean**	**>99**	**78** ^[a]^	**catalyst**	**this work**

[a] Isolated yield. [b] Calculated yield.

In addition to direct electrolysis, *N*‐oxyl radicals are commonly used catalysts for the indirect oxidation of primary and secondary alcohols.^17^ Particularly, 2,2,6,6‐tetramethylpiperidinyl‐*N*‐oxyl (TEMPO) and its derivatives are common oxidants with industrial‐ and laboratory‐scale applications.^18^ Under electrochemical conditions, the formation of the active reactant from persistent organic radicals can be accomplished in the absence of chemical oxidants.^19^, ^20^ The catalyst‐promoted electrooxidative synthesis of DFF in a biphasic system, using 4‐acetamido‐TEMPO and a recyclable NaHCO_3_ (aq)/KI electrolyte, was demonstrated.^21^

In the pursuit of sustainable chemical transformations, catalyst recovery plays a pivotal factor for meeting ecological and economical demands. The recovery and reuse of TEMPOs have generally required a great variety of solid‐supported heterogeneous and homogeneous organic supports.^22^ Although the recovery of insoluble catalysts is straightforward, the catalytic activity and selectivity may become impaired when anchored to solid carriers. On the contrary, homogeneous catalysts could grant exceptional activity and selectivity, but their inefficient recovery is a problem yet to be solved.^23^

Organic solvent nanofiltration (OSN) is a sustainable recycling technique for homogeneous catalysts.^24^ Its scale‐up and implementation in continuous processes are rather straightforward, therefore feasible for industrial utilizations. As the efficiency of separation is largely dependent on the molecular weight gap between the catalyst and the other solutes, size‐enlargement of small catalysts is generally required. Consequently, herein, we explore commercial and size‐enlarged TEMPO catalyst recovery by means of magnetism, microfiltration, and nanofiltration (Scheme 1).

Recently, we have demonstrated that the MCM‐41‐supported metal catalyst promoted the conversion of carbohydrates into HMF,^25^ and here we report a TEMPO‐mediated electrocatalytic oxidation method for the selective transformation of HMF into DFF (Scheme 1). The commercially available compact electrolysis cell (IKA ElectraSyn 2.0) as reactor, green solvents (MeCN, γ‐valerolactone, Rhodiasolv^®^ PolarClean), and non‐precious‐metal‐based electrodes (graphite, stainless steel) were selected. To the best of our knowledge, this is the first report on using a non‐precious‐metal‐based electrode for selective HMF conversion. With rational catalyst design, supported by quantum chemical studies, a new homogeneous size‐enlarged *C*_3_‐symmetric tris‐TEMPO derivative (**Hub^1^**) was synthesized to facilitate the recovery of the catalyst by nanofiltration. A comparison of the recovery and catalytic performance of commercially available TEMPO derivatives (SiliaCAT^®^ TEMPO, TurboBeads™ TEMPO) and the OSN compatible **Hub^1^‐TEMPO** was performed.

## Results and Discussion

### Electrocatalytic oxidation of HMF to DFF

Electrocatalytic oxidation can be performed directly when electron transfer (ET) between the substrate and the anode takes place at the electrode surface in a heterogeneous manner. By employing a catalyst, the ET involving the substrate becomes a homogeneous process. The latter indirect method can mitigate over‐oxidized side product formation and electrode passivation, which is essential for developing a sustainable process. Therefore, the oxidation of HMF gained from fructose25b was investigated in a direct process by using a galvanostatic setup (current: 1 mA).

**Figure 2:**
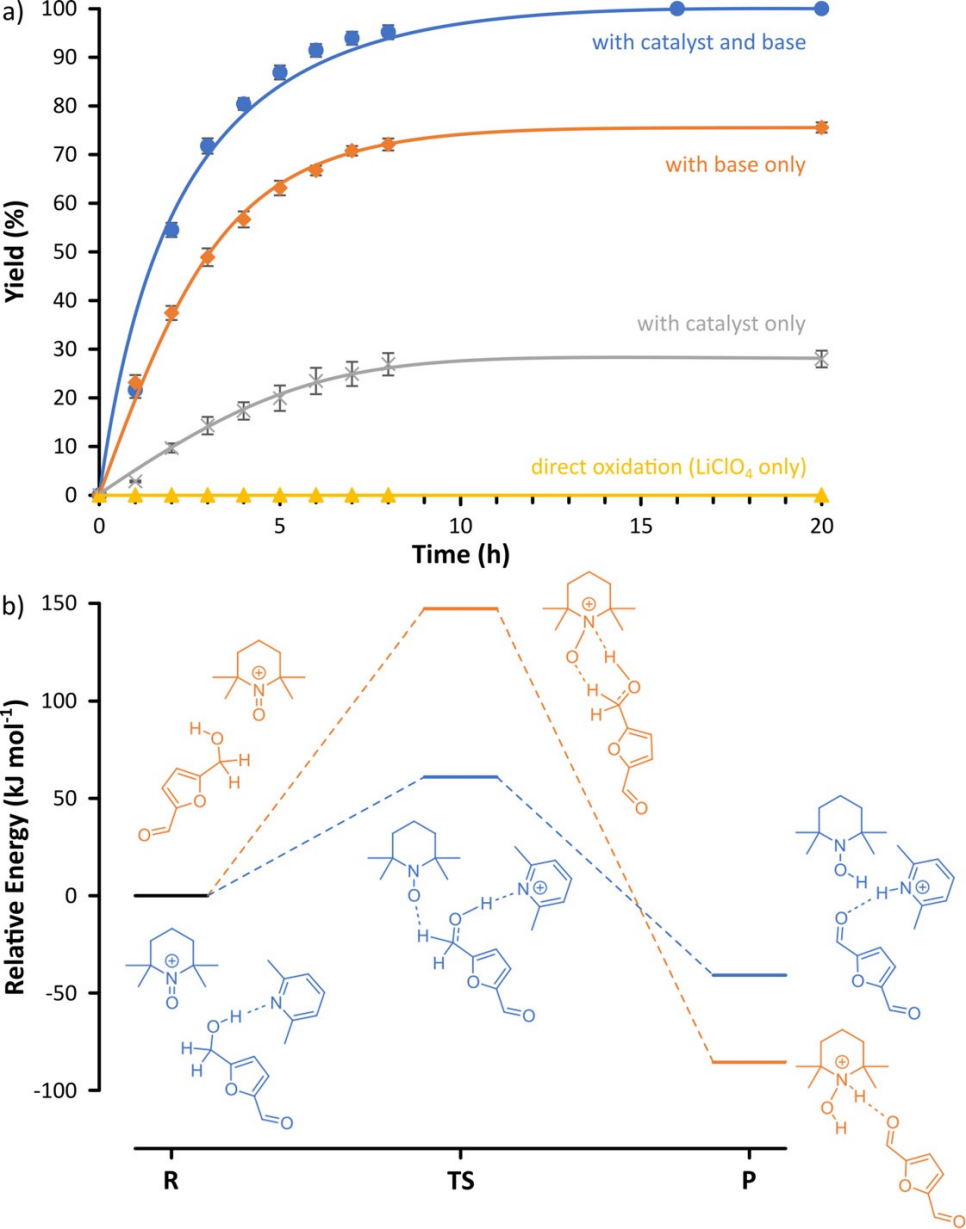
Synergistic effects of the TEMPO catalyst and 2,6‐lutidine base during the electrooxidation of HMF using (a) experimental and (b) computation methods. R: reactant, TS: transition state, P: product. Refer to the Supporting Information for the comparison of the reaction kinetics.

Graphite (anode) and stainless steel (cathode) were chosen to achieve cost‐effective operation.^26^ This arrangement improved sustainability as the commonly used platinum electrodes were replaced (Table 1). The direct oxidation with the LiClO_4_ electrolyte avoided product formation, whereas the addition of the 10 mol % TEMPO catalyst enabled a reaction with a moderate yield of 28 % (Figure 2 a). The addition of 2,6‐lutidine as the base resulted in virtually complete consumption (more than 99 %) of the starting material (HMF) without the formation of undesired byproducts. To analyze whether the base alone or the combined effect of the catalyst–base pair caused the increased yield, the electrochemical oxidation was also performed in the presence of the base but without TEMPO. Although the yield decreased to 76 %, it was still found to be higher than that of the TEMPO‐catalyzed method (28 %). Consequently, the synergistic effect of the catalyst and the base were further investigated with computational methods (Figure 2 b).

The schematic pathways of hydride ion transfer for the conversion of HMF to DFF both in the presence and absence of the base are shown in the energy diagram (Figure 2 b). The presence of the base lowers the activation energy by 58 %. The base polarizes the O−H bond of HMF through hydrogen bonding. The hydride ion transfers from HMF to the catalyst in the transition state, which is followed by the product formation as a result of the completion of the hydride ion transfer. The calculated activation energy for the formation of DFF is 61.57 kJ mol^−1^.

**Figure 3:**
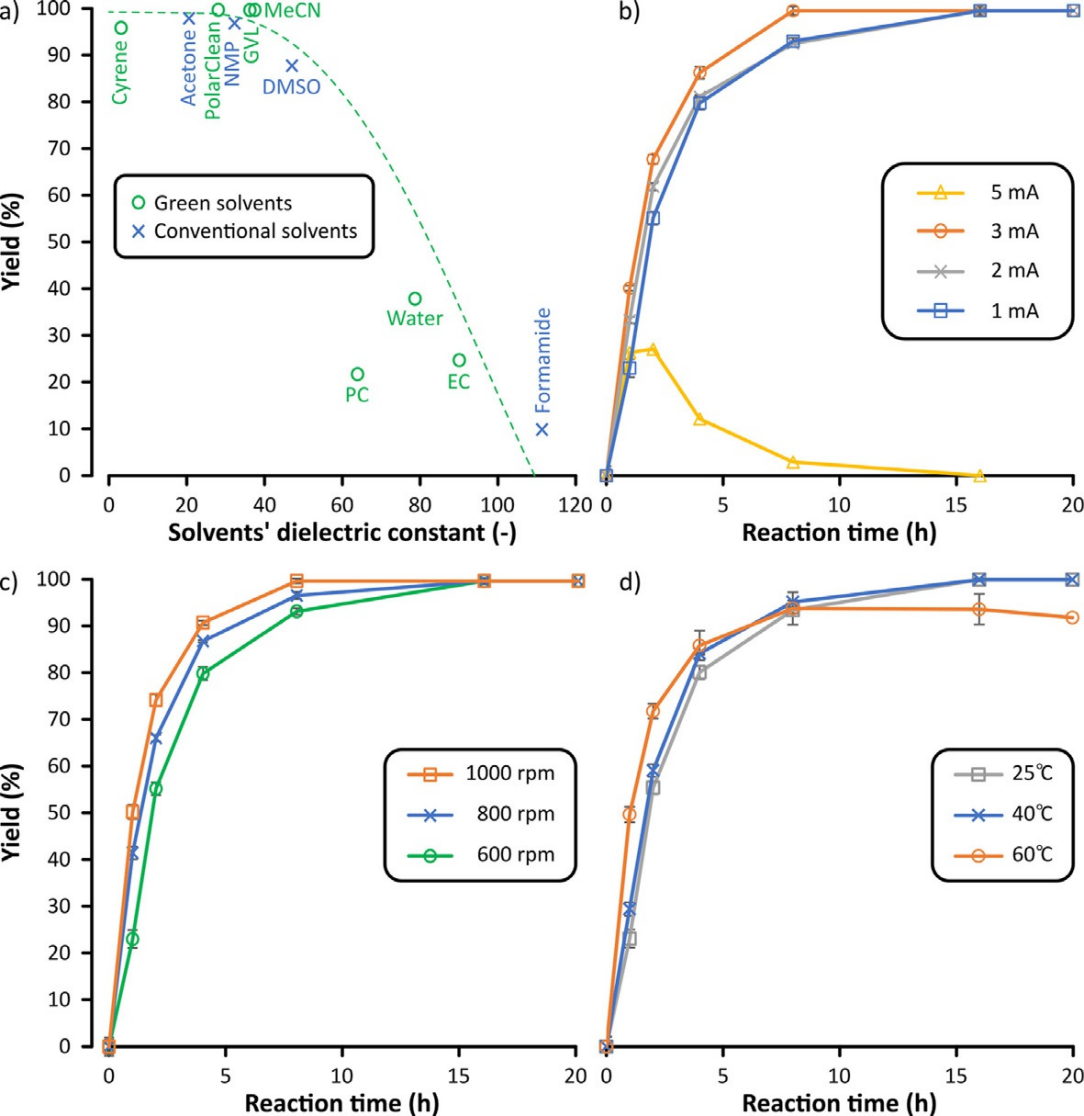
Effects of reaction parameters on the yield of electrocatalytic oxidation from HMF to DFF: (a) solvent, (b) current strength, (c) stirring rate, and (d) temperature. General reaction conditions: the reaction mixture was electrolyzed in 10 mL of solvent for 20 h at room temperature with a stirring speed of 600 rpm using a graphite anode and a stainless‐steel cathode at 1 mA current strength. Refer to the Supporting Information for the reaction kinetics.

After selecting the applicable catalyst/base system, the parameters influencing the synthesis of DFF were explored. First, 11 conventional and alternative solvents were tested in the oxidation process (Figure 3 a). The categorization of the solvents was done based on green solvent selection guides.^27^ Among these, solvents with dielectric constants higher than 40, such as ethylene carbonate (EC) and propylene carbonate (PC), water, and dimethyl sulfoxide (DMSO), provided lower yields and produced a considerable amount of unidentified side products. On the contrary, the use of solvents with dielectric constants lower than 40 resulted in excellent yields. This observation suggests that solvents with higher dielectric constants might have unfavorable effects on the solvation or stability of the ionic species. This could result in higher cell resistance, and consequently, in side product formation such as over‐oxidation or reaction with the solvent during the ET. The solvent effect on electrochemical processes is a complex matter and further investigations are needed in this field. Among the green solvents, the best results were achieved with γ‐valerolactone (GVL), PolarClean (methyl‐5‐(dimethylamino)‐2‐methyl‐5‐oxopentanoate), and acetonitrile (MeCN), as complete conversions were obtained without side reactions. Both GVL and PolarClean are emerging green solvents with the potential to pave the way toward sustainable electrolysis.^28^ To the best of our knowledge, this is the first application of PolarClean in electrocatalytic reactions.

The effect of current strength on the oxidation was examined (Figure 3 b). Increasing the electric current to 2 and 3 mA resulted in no significant change in the outcome of the reaction. However, after 8 h of constant current electrolysis at 5 mA, almost no product could be detected in the reaction mixture owing to accelerated side product formation. Deformation of the electrodes was also observed (see the Supporting Information).

Owing to the heterogeneous nature of the electrochemical process, the effects of both stirring rate (Figure 3 c) and reaction temperature (Figure 3 d) were investigated. Although higher stirring speed resulted in slightly faster product formation, no significant change in yield was observed by increasing the temperature from room temperature to 40 °C. At a more elevated temperature (60 °C), a small decrease in the yield was detected after 8 h, possibly owing to the over‐oxidation of the desired DFF. The increase in the concentration of TEMPO to 20 and 30 mol % resulted in virtually no change in the reaction. Moreover, a reticulated vitreous carbon (RVC) electrode was also tested as the anode (instead of graphite), but despite its larger surface area, no significant change in the rate of the reaction was observed. Also, the delicate structure of the RVC electrode presented additional difficulties in comparison to the standard graphite electrode. Refer to the Supporting Information for further details.

### Catalyst design and recovery

**Figure 4:**
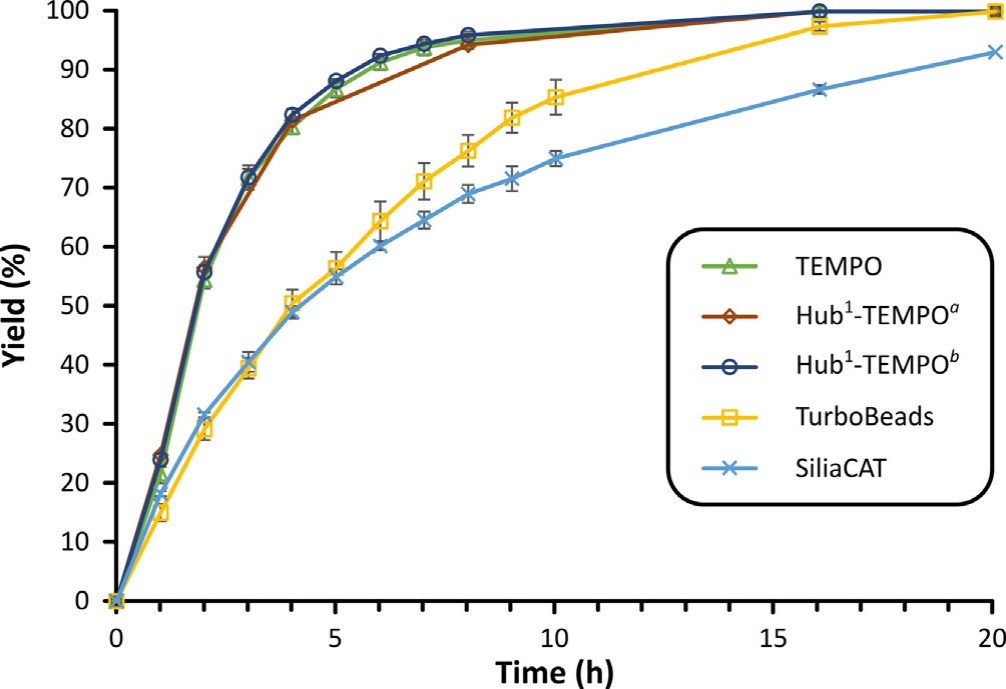
Comparison of homogeneous and solid‐supported TEMPO derivatives in the oxidation of HMF. For the kinetic comparison refer to the Supporting Information. [a] 10 mol % catalyst (3 equiv. active units). [b] 3.3 mol % catalyst (1 equiv. active unit).

Two commercially available solid‐supported TEMPO catalysts (TurboBeads: 50 nm diameter and 15 m^2^ g^−1^, SiliaCAT: 1.2 μm diameter and approx. 500 m^2^ g^−1^) were applied in the electrocatalytic oxidation of HMF under the optimized reaction conditions (Figure 4). Both compounds are heterogeneous catalysts with the TEMPO units immobilized on iron oxide cores and silica gel, respectively. These inert and resistant solid supports enable the facile separation of the catalyst from the reaction mixture by using an external magnetic field and microfiltration, respectively. A moderately slower reaction rate was observed in comparison to the homogeneous TEMPO system (Figure S9 in the Supporting Information). The TurboBeads provided full conversion after 16 h, whereas SiliaCAT provided a good yield (93 %) in 20 h. The reactions for both heterogeneous catalysts can be described with pseudo‐first‐order kinetics (*k*_TurboBeads_=0.1868 s^−1^, *k*_SiliaCAT_=0.1335 s^−1^), whereas no satisfactory correlation for the homogeneous TEMPO was observed.

To overcome the difficulties in recovering the native TEMPO and the slower reaction rate of the heterogeneous TEMPO derivatives, a size‐enlarged TEMPO for membrane recovery was designed in silico. Catalysts with high molecular weight (*M*_W_) exhibit high retention by nanofiltration membranes.

**Figure 5:**
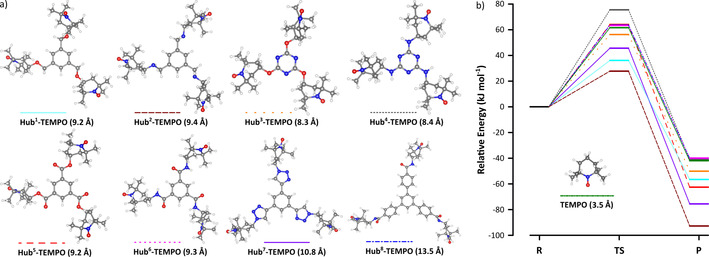
Size‐enlarged catalyst design. (a) Geometric structures of substituted TEMPO catalysts after optimization in acetonitrile medium (the radii of the molecules appear in parentheses) and (b) relative energy profiles for the conversion of HMF to DFF. For more detailed figures refer to the Supporting Information.

Owing to the small *M*_W_ gap between TEMPO and the other reaction components in the electrocatalytic oxidation, size‐enlargement of TEMPO was required to facilitate its recovery by organic solvent nanofiltration (OSN). Catalyst anchoring to soluble macromolecules^29^ or small trifunctional hubs^30^ is an efficient approach used in the recycling of high‐value organocatalysts. The latter approach exploits the *C*_3_‐symmetric multifunctional hub to provide straightforward synthesis, facile characterization, and high catalytic unit to inactive moiety ratio. The hub, bond type, or bond length between the hub and the catalyst allows fine‐tuning of the catalytic activity and enables catalyst recovery (Figure 5).

**Figure 6:**
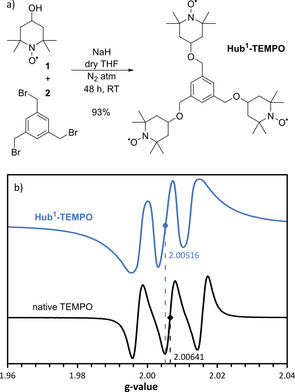
(a) Synthesis and (b) electron paramagnetic resonance spectra of the sized‐enlarged catalyst **Hub^1^‐TEMPO**.

Accordingly, eight size‐enlarged TEMPO derivatives (**Hub** ^***x***^ **‐TEMPO**) were considered for the oxidation of HMF (Figure 5) by using the M062X/6‐31G* level of the density functional theory (DFT) as implemented in the Gaussian software. Three hubs, namely benzene, 1,3,5‐triazine, and 1,3,5‐triphenylbenzene, and five covalent bonds, namely ether, amine, ester, amide, and 1,2,3‐triazole, were studied. The triazine type **Hub^3^‐TEMPO** has the smallest radius (8.3 Å), whereas the **Hub^8^‐TEMPO** with the triphenylbenzene unit has the largest (13.5 Å). The size‐enlargement resulted in an increase in the catalyst radius by as much as 286 %. The corresponding energy diagrams for the formation of DFF when using the different TEMPO derivatives are shown in Figure 5 b. The activation energy for the formation of DFF is lower for **Hub^1–3^‐** and **Hub^7^‐TEMPO**s than for the native TEMPO. Therefore, we conclude that the formation of DFF from HMF is kinetically more favorable when using these derivatives rather than the others, including the native TEMPO. In particular, **Hub^2^‐TEMPO**, closely followed by **Hub^1^‐TEMPO**, showed the lowest activation and product energies of 27.73 and −92.80 kJ mol^−1^, respectively. The synthesis of **Hub^1^‐TEMPO** was found to be the most straightforward through the *O*‐alkylation of 4‐hydroxy‐2,2,6,6‐tetramethylpiperidinyl‐*N*‐oxyl (**1**) with 1,3,5‐tris(bromomethyl)benzene (**2**), which produced the catalyst with excellent yield (93 %, Figure 6 a). To confirm its structure by NMR spectroscopy (Figures S25 and S26 in the Supporting Information), **Hub^1^‐TEMPO** was successfully reduced with l‐ascorbic acid (Scheme S9 in the Supporting Information). Refer to the Supporting Information for the experimental protocol and spectra.

Electron paramagnetic resonance (EPR) spectroscopy showed a downshift in the *g*‐value of the solvated native TEMPO (from 2.00641 to 2.00516) as a result of the size‐enlargement (Figure 6 b).

This phenomenon means that the free radical electrons are more loosely bound to the nitrosyl oxide, which could enhance the catalytic activity. These findings are in line with the predictions of the DFT study.

**Figure 7:**
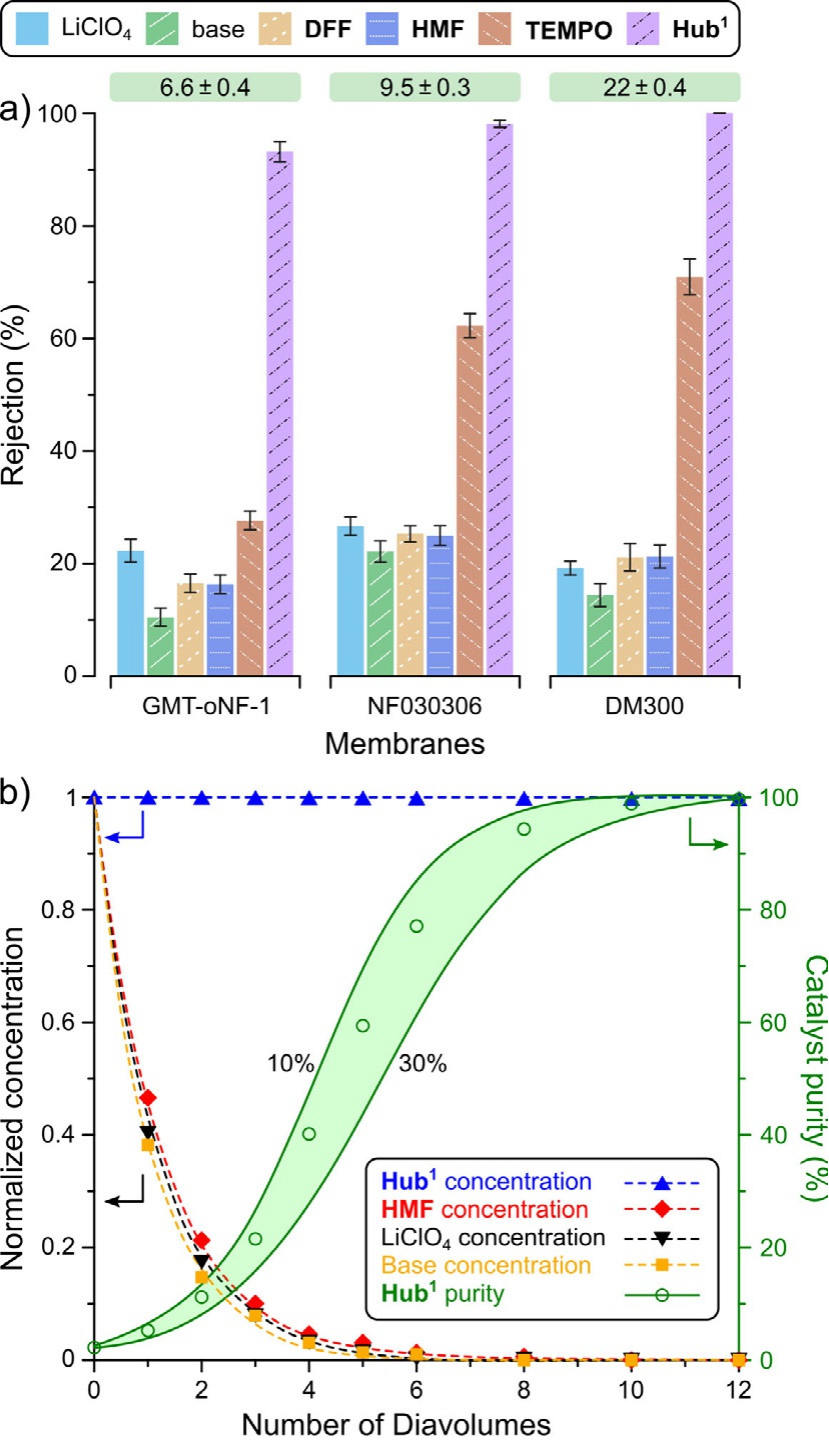
(a) Solute rejection for different membranes in acetonitrile at 30 bar. The values in the boxes above each membrane are fluxes expressed in L m^−2^ h^−1^. (b) Solute concentration and purity profiles during catalyst recovery by diafiltration. The curves are modeled, whereas the symbols are experimental datapoints. The area in green represents the catalyst purity when the other solutes show rejections between 10 % and 30 %.

Nonetheless, in comparison to the native TEMPO, the homogeneous **Hub^1^‐TEMPO** added in equivalent mole percentage showed no significant differences in yield or the progression of the reaction (Figure 4). Even when the catalyst was used such that an equivalent amount of TEMPO units was present in the reaction mixture (one third the mole percentage in comparison that of the native TEMPO), virtually no change was observed in the catalytic performance. We can conclude that the size‐enlargement did not adversely affect the catalytic performance. The GMT‐oNF‐1, NF030306, and DM300 membranes were screened to identify the most suitable membrane for the catalyst recovery by diafiltration (Figure 7 a). Based on the molecular weights, the rejection gap between the commercial TEMPO and the other solutes (approx. 50 %), as well as the absolute rejection of TEMPO (approx. 30–70 %) were not sufficiently large for successful diafiltration. On the contrary, the rejection of **Hub^1^‐TEMPO** was found to range between 90 % and 100 % for all the membranes. In particular, DM300 fully retained the enlarged catalyst while still being able to effectively purge all other solutes, showing rejection as low as 10–20 %. DM300 also demonstrated a high flux of 22±0.4 L m^−2^ h^−1^, which was 3.3 and 2.3 times higher than that of the GMT‐oNF‐1 and NF030306 membranes, respectively. Consequently, DM300 was chosen for the catalyst recovery using diafiltration (Figure 7 b). The concentration profile revealed that the solutes were completely purged out of the system within 10–12 diavolumes, and the catalyst purity reached 100 %. The highlighted area shows the results of the mathematical modeling for the catalyst purity when the other solutes showed rejections between 10 % and 30 %, requiring 10 and 12 diavolumes to reach virtually 100 % catalyst purity. This result demonstrates the robustness of the proposed nanofiltration‐based catalyst recovery.

## Conclusions

Biomass‐derived HMF was successfully converted to DFF with 78 % isolated yield and virtually 100 % selectivity by utilizing the compact ElectraSyn reactor in a galvanostatic setup in an undivided cell for environmentally friendly organic electrosynthesis. In comparison to the previous literature reports that employed platinum as the electrode material, graphite (anode) and stainless steel (cathode) were chosen to achieve cost‐effective operation in this study. Among the green solvents tested, PolarClean was successfully used in electrocatalysis for the first time. The effects of current strength, stirring rate, temperature, catalyst molar ratio, electrode surface area, and the roles of TEMPO and the lutidine base on the electrooxidation were explored both experimentally and through DFT modeling. Computer‐aided modeling was used for size‐enlarged catalyst design and structure optimization. The reaction pathways of the electrocatalytic conversion were determined, and the relative energy profiles of the native and designed catalysts were compared. Synergetic effects of TEMPO and lutidine were observed, ensuring high yield and selectivity simultaneously. The homogeneous size‐enlarged *C*_3_‐symmetric tris‐TEMPO derivative was successfully recovered by using organic solvent nanofiltration.

## Experimental Section

### Materials

Rhodiasolv® PolarClean HSP was purchased from Solvay, whereas γ‐valerolactone and dimethyl sulfoxide were obtained from Alfa Aesar. Acetonitrile, *n*‐hexane, propylene carbonate, and THF were bought from Merck, whereas acetone, anhydrous sodium carbonate, sodium chloride, and hydrochloric acid were supplied by Sinopharm Chemical Reagent Co. Ltd. (Shanghai, China). Ethyl acetate was purchased from either Merck or Sinopharm. All these compounds were used without further purification. Type II Millipore water was used. TEMPO (Alfa Aesar), 4‐OH‐TEMPO (Merck), SiliaCAT TEMPO (Merck), TurboBeads™ TEMPO (Merck), 1,3,5‐tris(bromomethyl)benzene (Fluorochem), 2,6‐lutidine (Merck), HMF (Merck, Alfa Aesar, or prepared based on our previous procedure25b), sodium hydride, l‐ascorbic acid, and LiClO_4_ (Merck) were used as supplied. Choline chloride (ChCl) and fructose were purchased from Aladdin Chemical Technology Co. Ltd. (Shanghai, China).

The electrochemical experiments were carried out by using an IKA ElectraSyn 2.0 potentiostat equipped with either a single vial holder, or a six‐reaction carousel, or a GOGO module connected to an IKA KS 4000 i control shaker. The reactions were conducted in constant current mode, without a reference electrode. The electrodes and vials were purchased from IKA. The electrodes were washed multiple times with water, and acetone, and were rubbed dry with tissue paper before each use.

Infrared spectra were recorded with a Bruker Alpha‐T FTIR spectrometer (s: strong, m: medium, w: weak). Electron paramagnetic resonance spectroscopy was carried out in an EPR spectrometer (Xenon series from Bruker) at room temperature, and the unit was operated in the X‐Band mode with a microwave frequency of 9.4–9.8 GHz and a modulation frequency of 100 kHz. An ER 221 Bruker cell tube with an inner diameter of 3 mm and an outer diameter of 4 mm was used to load the samples. For solid state measurements, the samples were mixed with KBr powder to dilute the concentration. The sweep width was set at 600 G with a modulation amplitude of 0.4 G. The radio frequency power was set to 0.6325 mW with power attenuation of 25 dB. For solvated state measurements, the samples were solvated with acetonitrile. The sweep width was set at 8000 G with a modulation amplitude of 4 G. The radio frequency power was set to 0.6325 mW with power attenuation of 25 dB. NMR spectra were recorded either with a Bruker DRX‐500 Avance spectrometer (at 500 MHz for ^1^H and at 125 MHz for ^13^C spectra) or with a Bruker 300 Avance spectrometer (at 300 MHz for ^1^H and at 75.5 MHz for ^13^C spectra), as specified for each compound. High‐resolution mass measurements were performed with a Thermo Exactive plus EMR Orbitrap mass spectrometer, which was used with a Thermo Ultimate 3000 UHPLC with 100 % methanol as the mobile phase. Melting points were recorded with a Boetius micro‐melting point apparatus, and the observations were not corrected. Silica gel 60 F_254_ (Merck) plates were used for thin‐layer chromatography (TLC) and the spots were visualized either by ultraviolet light (254 nm) or by staining with an acidic H_2_O/EtOH solution of 2,4‐dinitrophenylhydrazine (DNP). Silica gel 60 (70–230 mesh, Merck) was used for column chromatography. The ratios of the solvents for the eluents are given in terms of volume (mL mL^−1^). Yields (except for isolated yields) were determined based on the HPLC chromatograms. For the detailed calculation procedure, refer to the Supporting Information.

### Particle size determination

The particle size of SiliaCAT was determined by means of dynamic light scattering using a Zetasizer Nanoseries instrument (Malvern Panalytical). The sample was dispersed in deionized water inside a glass cuvette cell with a square aperture and measured immediately after the preparation of the dispersion by shaking. The measurement was carried out at 25 °C with an equilibrium time of 120 s. Ten runs, each of duration 10 s, were performed for six data collections. For further details, refer to the Supporting Information.

### Synthetic procedure for the preparation of HMF from fructose

HMF was prepared based on our previously described procedure25b with some minor modifications: in a typical run, the conversion of fructose into HMF was conducted in a glass flask (500 mL) equipped with a condenser. The deep eutectic solvent system was formed with fructose (20 g, 0.11 mol, 1 equiv) and ChCl (60 g, 0.43 mol, 4 equiv). Then, HCl (0.2 mL, 37 %) was added as the catalyst. The flask was placed into an oil bath and heated (100 °C) with vigorous stirring. After the reaction was completed, the black mixture was dissolved in saturated NaCl solution (10 mL) and then extracted with ethyl acetate (5×30 mL). Anhydrous sodium carbonate (10 g) was added to the obtained organic solution and filtered to remove water and acid. Then, the organic solvent was removed with a rotary evaporator. The concentrate was dissolved in acetone (50 mL) and further distilled to obtain 13.2 g (95 %) HMF. The spectral data were fully consistent with those reported in the literature.25b

### General procedure for the electrochemical oxidation of HMF into DFF

Without taking precautions to exclude air and moisture, the ElectraSyn vial (5 mL) equipped with a stir bar was charged with HMF (31.5 mg, 0.25 mmol, 1.0 equiv), TEMPO (3.9 mg, 0.025 mmol, 0.1 equiv), 2,6‐lutidine (29 μL, 0.25 mmol, 1.0 equiv), LiClO_4_ (53.2 mg, 0.5 mmol, 2.0 equiv), and MeCN (5 mL). The ElectraSyn vial cap equipped with the anode (graphite) and cathode (stainless steel) was inserted into the mixture. The reaction mixture was electrolyzed at a constant current of 1 mA for 20 h. Then, the vial cap was removed, and the electrodes were rinsed with CH_2_Cl_2_ (10 mL), which was combined with the reaction mixture. The crude mixture was concentrated under reduced pressure. The resulting mixture was taken up in CH_2_Cl_2_ (25 mL) and washed three times with water (10 mL). The organic phase was dried over anhydrous MgSO_4_ and concentrated in vacuo. The crude product was purified by preparative TLC (SiO_2_, CH_2_Cl_2_/MeOH 40:1) to give DFF (48 mg, 78 %) as a white solid. The yield was recorded as the average of three parallel experiments (standard deviation: ±2 %). *R*_f_=0.73 (SiO_2_, CH_2_Cl_2_/MeOH 20:1, visualized by DNP); m.p. 106–109 °C (lit.^15^ 108–109 °C); ^1^H NMR (500 MHz, CDCl_3_): *δ*_H_=9.86 (2 H, s, CHO), 7.35 ppm (2 H, s, CH); ^13^C NMR (125 MHz, CDCl_3_): *δ*_c_=179.4 (2 C, CHO), 154.3 (2 C), 119.6 ppm (2 C); IR (KBr): $\tilde{\nu }$_max_=3140 (m), 3128 (w), 3103 (m), 1681 (s), 1564 (w), 1511 (w), 1410 (m), 1266 (m), 1243 (m), 1189 (m), 1175 (m), 1022 (m), 1002 (w), 979 (m), 960 (s), 846 (w), 827 (m), 809 (m), and 797 cm^−1^ (m). The spectral data are fully consistent with those reported in the literature.^15^

### 1,3,5‐Tris((2,2,6,6‐tetramethylpiperidin‐*N*‐oxyl‐4‐yl)oxymethyl)benzene (Hub^1^‐TEMPO)

4‐OH‐TEMPO (**1**, 506 mg, 2.94 mmol, 3.5 equiv) was dissolved in dry THF (1 mL) in a dried round‐bottomed flask under N_2_ atmosphere. Next, NaH (60 % dispersion in mineral oil, 176 mg, 4.40 mmol, 5.25 equiv) was added, and stirred at room temperature until the intensive gas formation stopped. Then, a solution of 1,3,5‐tris(bromomethyl)benzene (**2**, 300 mg, 0.84 mmol, 1 equiv) in dry THF (1 mL) was added to the reaction mixture and stirred for 2 d, during which precipitation was observed. After completion of the reaction, MeOH (3 mL) was added dropwise, followed by evaporation under reduced pressure. The remaining material was taken up in ethyl acetate (25 mL) and washed three times with water (10 mL). The organic phase was dried with anhydrous MgSO_4_ and concentrated under reduced pressure. The crude product was purified by column chromatography with gradient elution (SiO_2_, Hex/EtOAc 1:1 to 2:3) to yield **Hub^1^‐TEMPO** (493 mg, 93 %) as an orange solid. The structure of **Hub^1^‐TEMPO** was confirmed by the NMR spectra of the N‐OH derivative (**S1**). Refer to the Supporting Information for further details. *R*_f_=0.55 (SiO_2_, Hex/EtOAc 1:1); m.p. 100–103 °C; IR (KBr): $\tilde{\nu }$_max_=2991 (m), 2976 (s), 2937 (m), 2878 (m), 1610 (w), 1465 (m), 1396 (w), 1374 (m), 1360 (s), 1347 (s), 1308 (w), 1289 (w), 1244 (m), 1220 (m), 1191 (m), 1177 (s), 1154 (s), 1105 (s), 1026 (w), 1015 (w), 956 (w), 902 (w), 854 (m), 846 (w), and 685 cm^−1^ (w); HRMS (ASAP^+^): *m*/*z* calcd for C_36_H_60_N_3_O_6_: 630.4477 [*M*]^+^; found: 630.4474; calcd for C_36_H_61_N_3_O_6_: 631.4555 [*M*+H]^+^; found: 631.4551. The solvated and solid‐state EPR spectra of **Hub^1^‐TEMPO** can be found in the Supporting Information.

### Organic solvent nanofiltration

**Figure 8:**
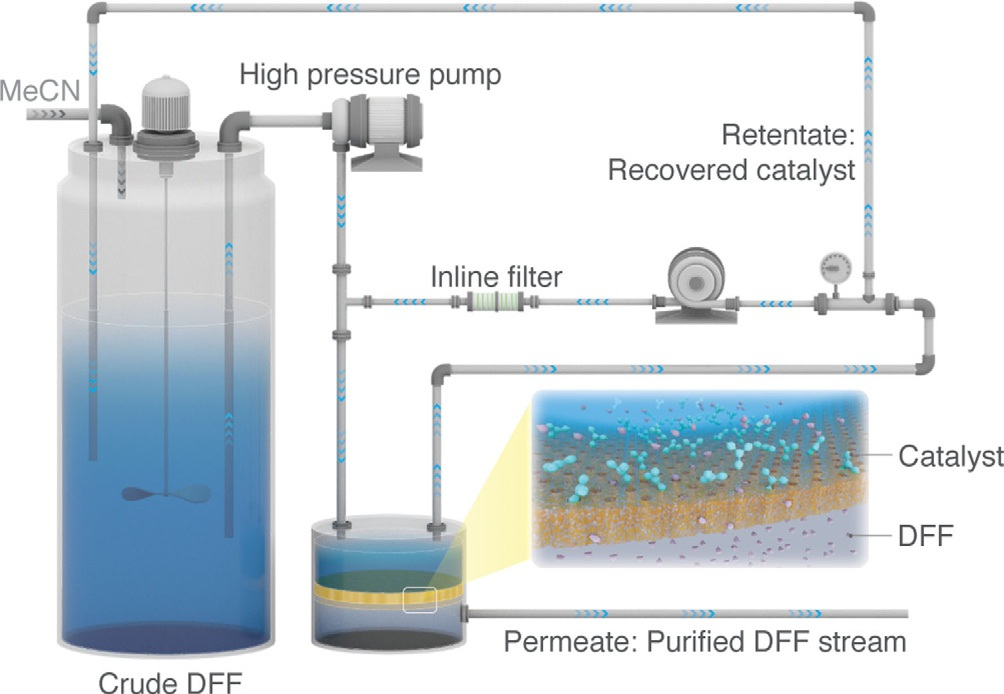
Cross‐flow nanofiltration apparatus for catalyst recovery.

The membrane separations were performed by using a typical crossflow nanofiltration rig (Figure 8). A Michael–Smith–Engineers gear pump was used for the recirculation of the retentate, and the speed was set at 1.2 L min^−1^. The commercial membranes were washed with acetonitrile and conditioned under a pressure of 30 bar for 24 h prior to rejection and flux measurements to ensure that the system reached a steady state. The solvent flux was determined by measuring the volume of the solvent permeating through the membrane within a given time for a certain surface area. The solute rejection was obtained from the ratio of the permeate and retentate concentrations of the solutes. The diafiltration of the crude reaction mixture was carried out at 30 bar using a DM300 membrane with an active area of 52 cm^2^. Fresh acetonitrile was continuously fed into the vessel to compensate for the solvent volume leaving the system through the permeate stream, thereby maintaining a constant system volume. Samples of the permeate and retentate streams were periodically taken for analysis. The number of diavolumes, defined as the volume ratio of the permeate and retentate streams at a given time, was used to describe the progress of the filtration. The recovered catalyst was reused multiple times, and its characterization is shown in Figures S33 and S34 in the Supporting Information.

### Computational methods

All quantum chemical calculations for the conversion of HMF to DFF were performed with the Gaussian 09 package^31^ and the ground‐state geometries were optimized by using the hybrid meta‐exchange‐correlation functional M062X with the 6‐31G* basis set. The transition states were analyzed by means of frequency calculation (single imaginary frequency). The polarizable continuum model was used for solvation.

## Conflict of interest

*The authors declare no conflict of interest*.

## Supporting information

As a service to our authors and readers, this journal provides supporting information supplied by the authors. Such materials are peer reviewed and may be re‐organized for online delivery, but are not copy‐edited or typeset. Technical support issues arising from supporting information (other than missing files) should be addressed to the authors.

Supplementary
