# Supplementary material for: Selective Electrocatalytic Oxidation of Biomass‐Derived 5‐Hydroxymethylfurfural to 2,5‐Diformylfuran: from Mechanistic Investigations to Catalyst Recovery
Source: ChemSusChem. 2020 Jun 2;13(12):3127–36. doi: 10.1002/cssc.202000453 (PMC7318667; doi:10.1002/cssc.202000453)
Supplement: Supplementary file 1 — Supplementary [file CSSC-13-3127-s001.pdf]

ChemSusChem

Supporting Information

Selective Electrocatalytic Oxidation of Biomass-Derived 5-Hydroxymethylfurfural to 2,5-Diformylfuran: from Mechanistic Investigations to Catalyst Recovery

Peter Kisszekelyi,[a] Rifan Hardian,[b] Hakkim Vovusha,[c] Binglin Chen,[d] Xianhai Zeng,[d, e] Udo Schwingenschlögl,[c] Jozsef Kupai,*[a] and Gyorgy Szekely*[b, f]

Table of Contents

1. Electrochemical oxidation of HMF into FDCA 3
2. Rational catalyst selection 4
2.1. Proposed synthesis routes of size-enlarged C3-symmetric TEMPO derivatives 4
2.2. QM calculations 7
2.3. Comparison of the different C3-symmetric TEMPO derivatives 11
3. Methods and materials 13
4. TEMPO-mediated electrocatalytic oxidation – additional information 15
4.1. Schematic representation of the applied TEMPOs in the oxidation of HMF to DFF 15
4.2. Calculation method of the conversion and the yield 16
4.3. RVC vs Graphite electrode 18
4.4. Amount of TEMPO mediator 19
4.5. Constant current electrooxidation at 5 mA 19
4.6. Kinetic studies 20
5. Structure elucidation of C3-symmetric *tris*-TEMPO derivative (Hub^1-TEMPO) 23
5.1. Characterization of **Hub^1-TEMPO** 23
5.2. Reduction of **Hub^1-TEMPO** to N-OH derivative, characterization of **S1** 26
6. DFF characterization 30
7. Hub^1-TEMPO recovery 32
8. Particle size determination of SiliaCAT TEMPO 33
9. QM calculation coordinates 34
10. References 75

1. Electrochemical oxidation of HMF into FDCA

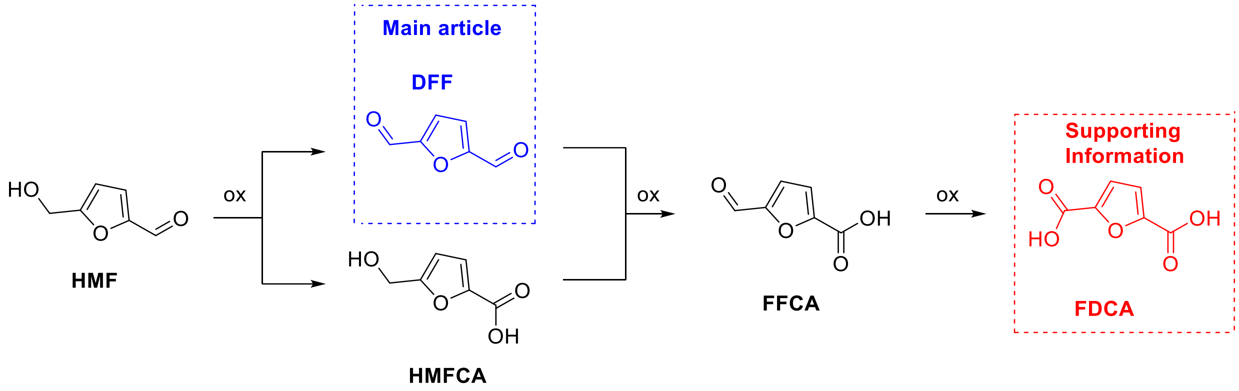

Scheme S1. Possible products during the electrochemical oxidation of HMF.

During the oxidation of HMF, when the reaction is not selective, several compounds can be isolated from the reaction mixture (**Scheme S1**). Oxidation of the OH group gives the desired DFF, but with the reaction of the CHO function HMFCA is formed. Both intermediates can be further oxidized into FFCA and that finally into FDCA as the compound with the highest oxidation state. Besides oxidation reactions, the beforementioned compounds can also react with each other forming additional by-products (ester, acetal, etc.). As comparison, some recently published methods for the electrocatalytic oxidation of HMF into FDCA were collected (**Table S1**). Both direct, and mediated approaches have been investigated. The reaction media is mostly aqueous, to facilitate the product formation.

Table S1. Recently published examples for the electrochemical oxidation of HMF to FDCA.[a]

Ref.	Anode/Cathode	Mediator	Solvent	Yield (%)
[1]	Au / Pt Or carbon felt / Pt	TEMPO	0.5 M borate buffer	99
[2]	Ni_2P NPA / carbon rod or Ni_2P NPA / Ni_2P NPA	-	1 M KOH (aq)	>99
[3]	Co-P / Co-P	-	1 M KOH (aq)	90
[4]	$Ni_xCo_{3-x}O_4$ / carbon rod	-	0.1 M KOH (aq)	90
[5]	carbon felt / Pt	TEMPO or ACT-TEMPO	0.5 M sodium borate buffer and 0.1 M NaOH (aq)	>95
[6]	carbon felt / Ag/C	ACT-TEMPO	0.5 M sodium borate buffer	98

[a] NPA: nanoparticle; ACT: acetamido

2. Rational catalyst selection

At the beginning of our work eight size-enlarged C3-symmetric TEMPO derivative structures were suggested to be examined as the mediator in the electrocatalytic oxidation of HMF. The **Hub^1-TEMPO** was selected for further experiments based on molecule radius, energy profile of DFF formation, stability of the formed covalent bond, number of synthetic steps and costs of starting materials.

2.1. Proposed synthesis routes of size-enlarged C3-symmetric TEMPO derivatives

The proposed synthesis procedures and the corresponding references based on analogous compounds are collected in Schemes S2–S8.

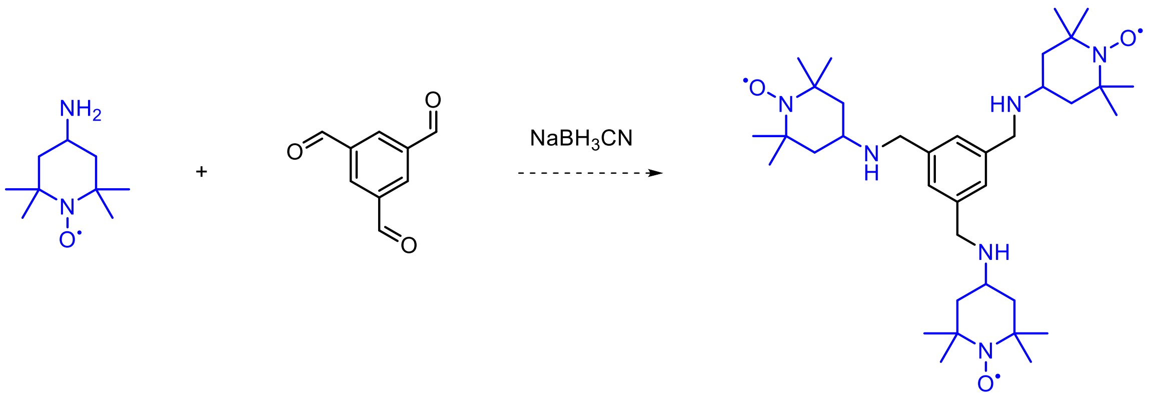

Hub^2-TEMPO

Scheme S2. Proposed synthesis plan of **Hub^2-TEMPO**.[7]

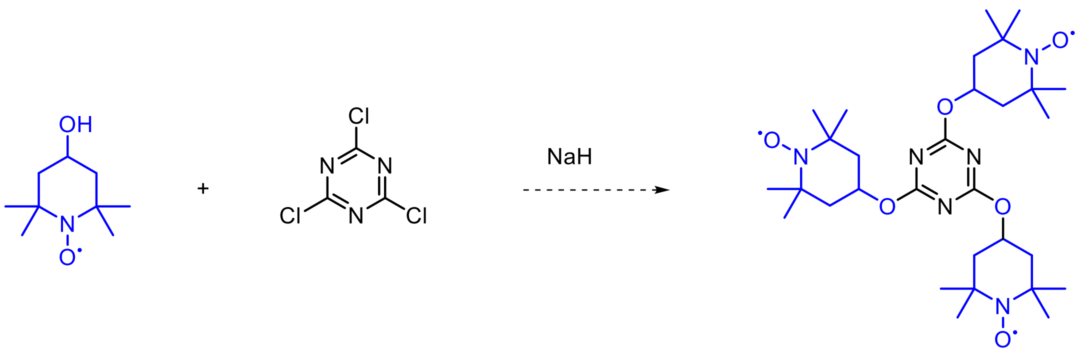

Hub^3-TEMPO

Scheme S3. Proposed synthesis plan of **Hub^3-TEMPO**.[8]

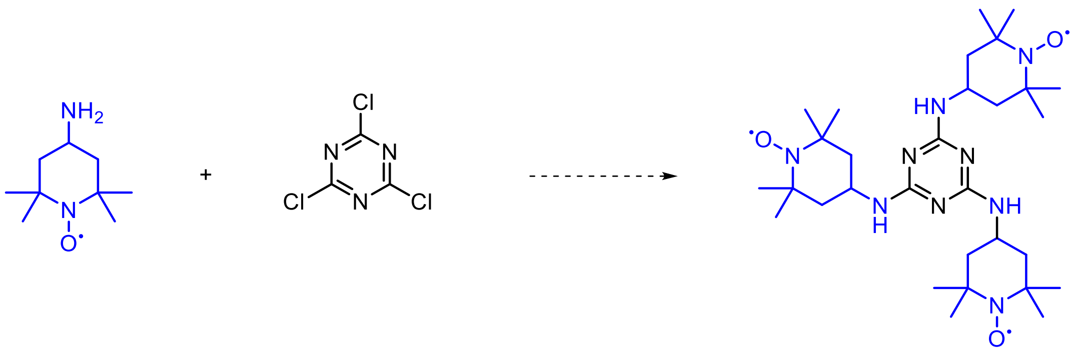

Hub^4-TEMPO

Scheme S4. Proposed synthesis plan of **Hub^4-TEMPO**.[8]

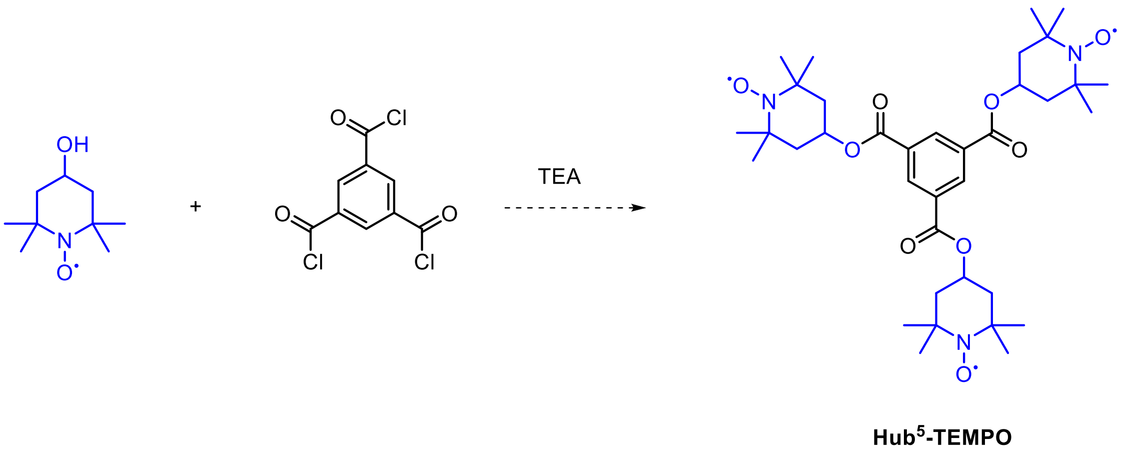

Scheme S5. Proposed synthesis plan of **Hub⁵-TEMPO**.[9]

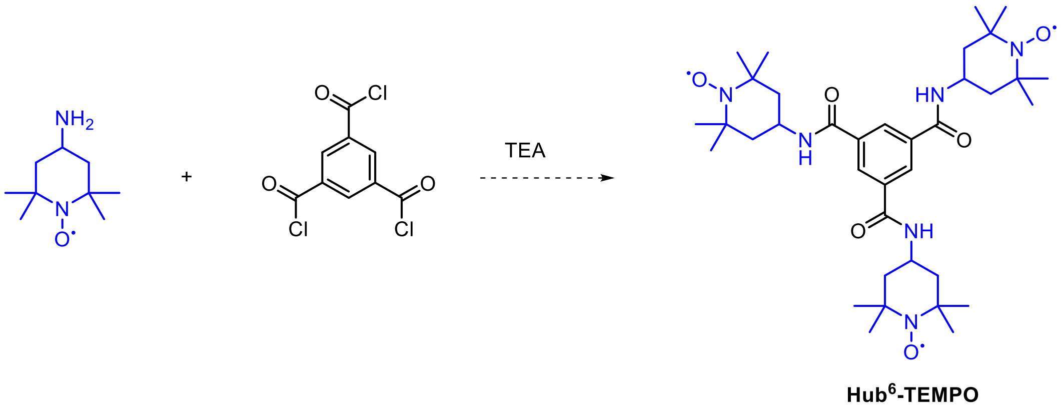

Scheme S6. Proposed synthesis plan of **Hub⁶-TEMPO**.[10]

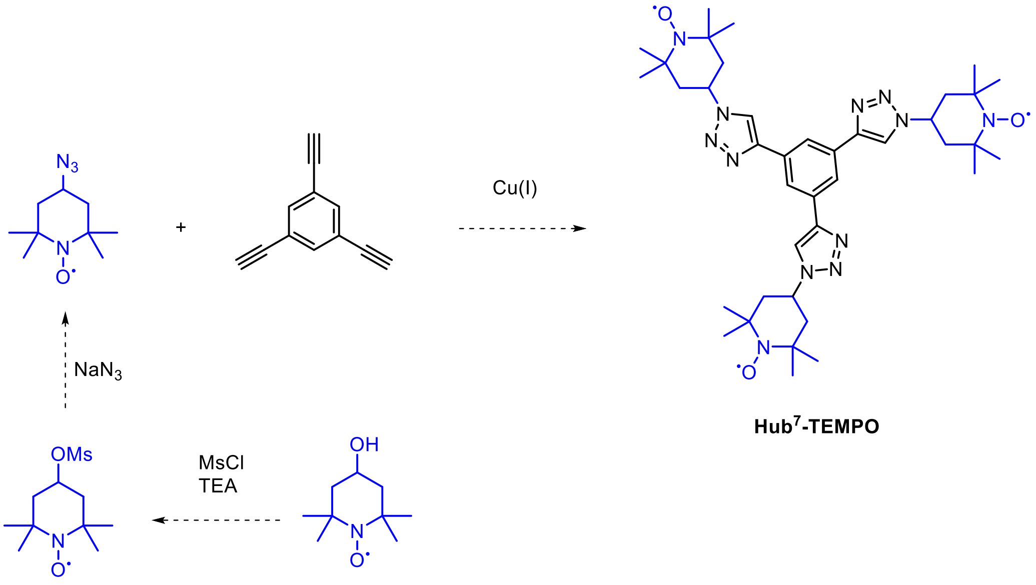

Scheme S7. Proposed synthesis plan of **Hub⁷-TEMPO**.[11]

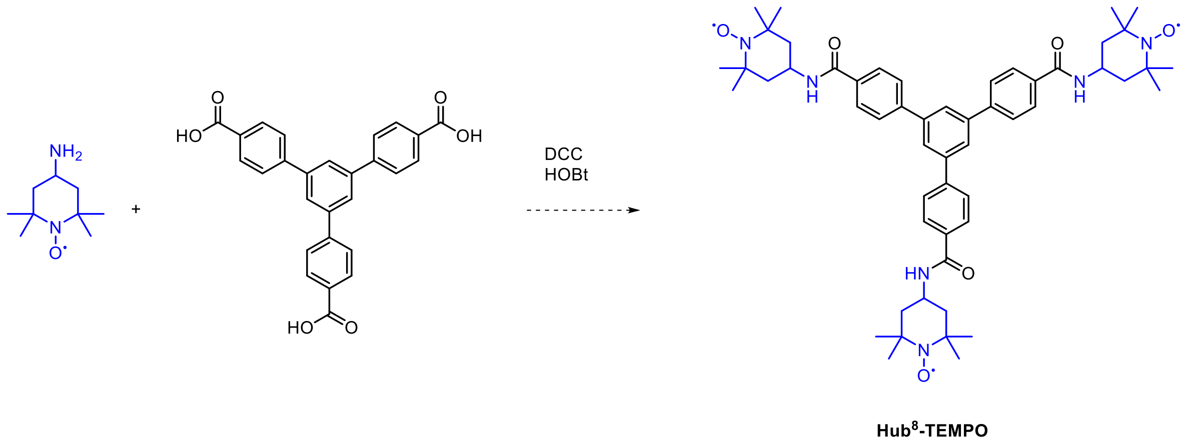

Scheme S8. Proposed synthesis plan of **Hub⁸-TEMPO**.[12]

2.2. *QM calculations*

For the oxidation of HMF eight size-enlarged TEMPO derivatives (**Hub^x-TEMPO**) were considered *in silico* using M062X/6-31G* level of density functional theory (DFT) as implemented in Gaussian software (Figure S1-S8). Three different hubs, namely benzene, 1,3,5-triazine and 1,3,5-triphenylbenzene; and five covalent bonds, namely ether, amine, ester, amide, and 1,2,3-triazol were studied.

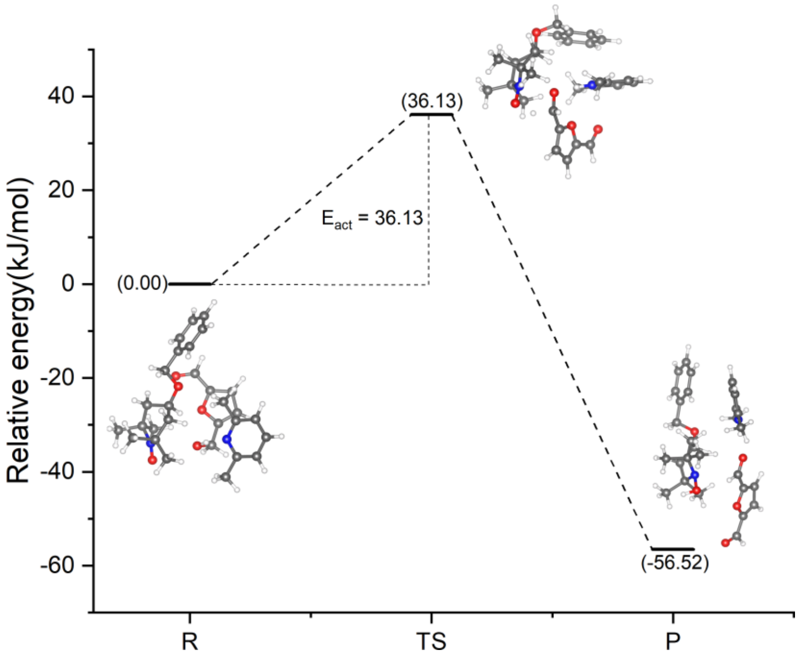

Figure S1. Relative energy profile for the conversion of HMF to DFF with **Hub^1-TEMPO** catalyst.

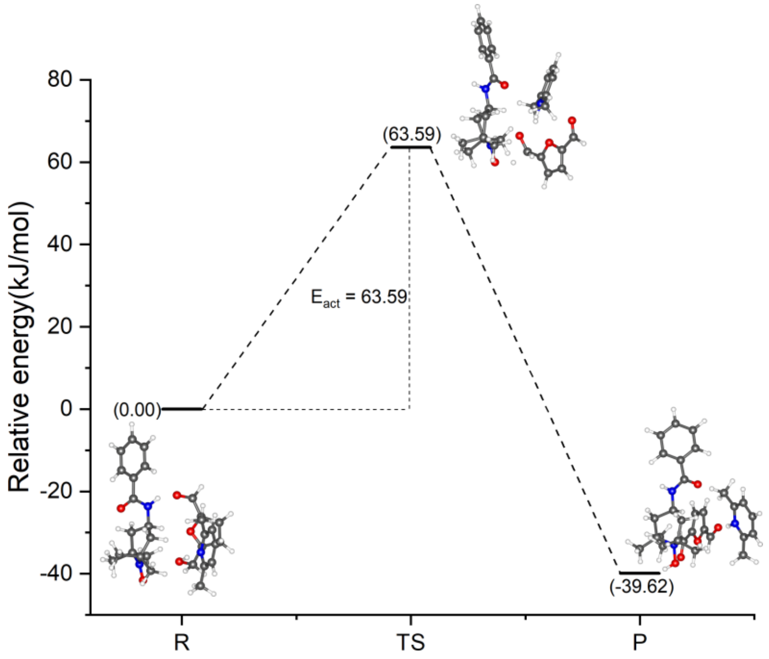

Figure S2. Relative energy profile for the conversion of HMF to DFF with **Hub[6]-TEMPO** catalyst.

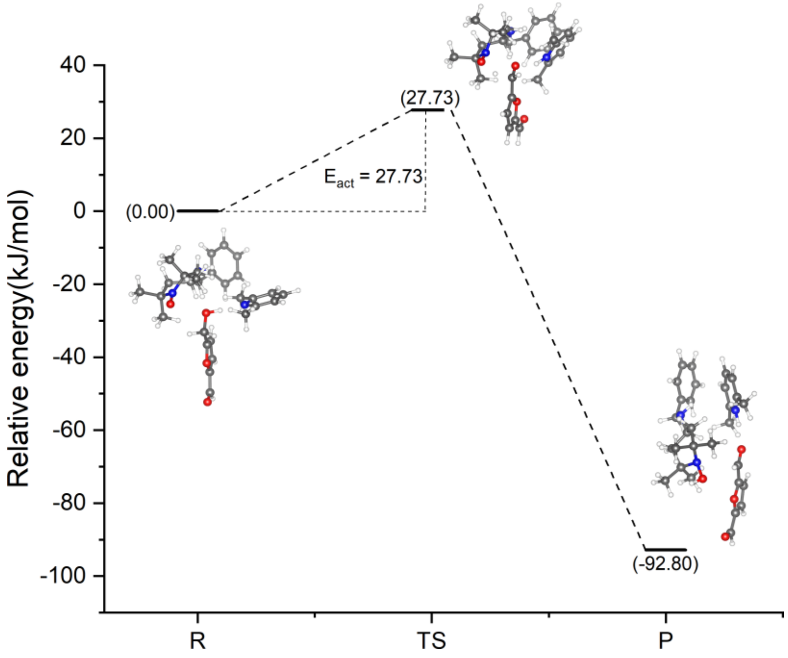

Figure S3. Relative energy profile for the conversion of HMF to DFF with **Hub[2]-TEMPO** catalyst.

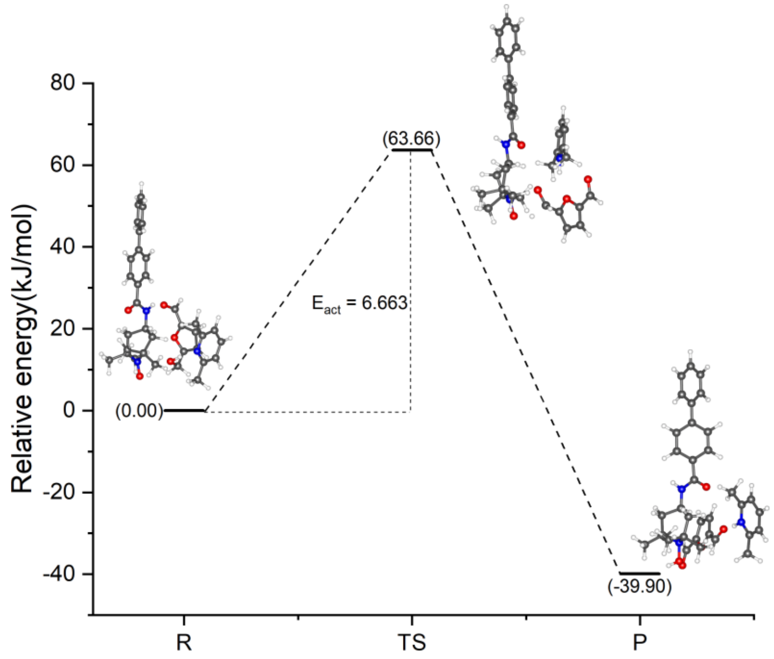

Figure S4. Relative energy profile for the conversion of HMF to DFF with **Hub[8]-TEMPO** catalyst.

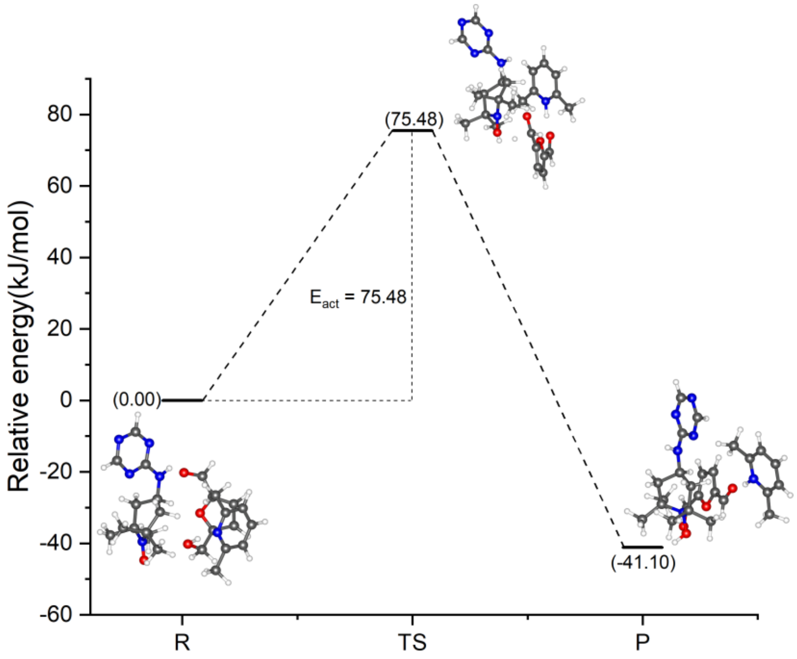

Figure S5. Relative energy profile for the conversion of HMF to DFF with **Hub[4]-TEMPO** catalyst.

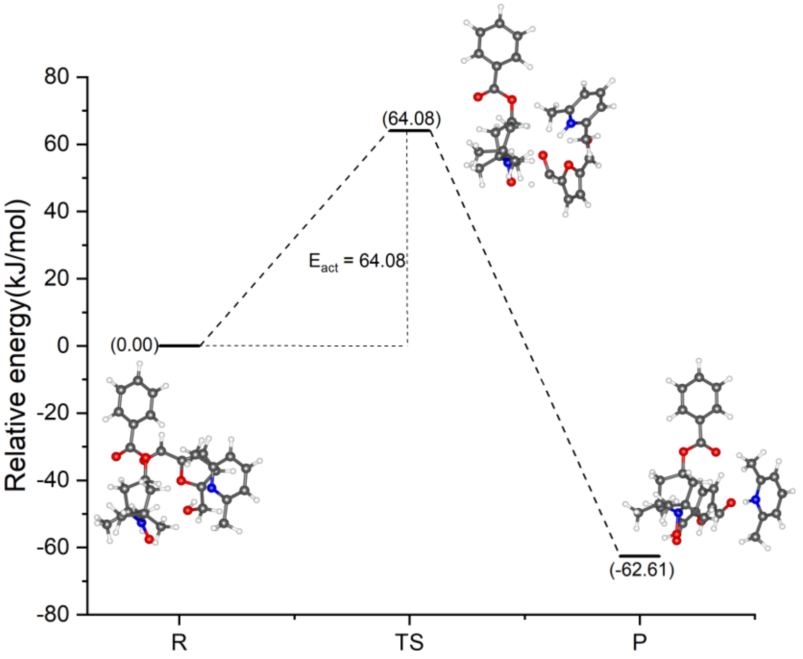

Figure S6. Relative energy profile for the conversion of HMF to DFF with **Hub[5]-TEMPO** catalyst.

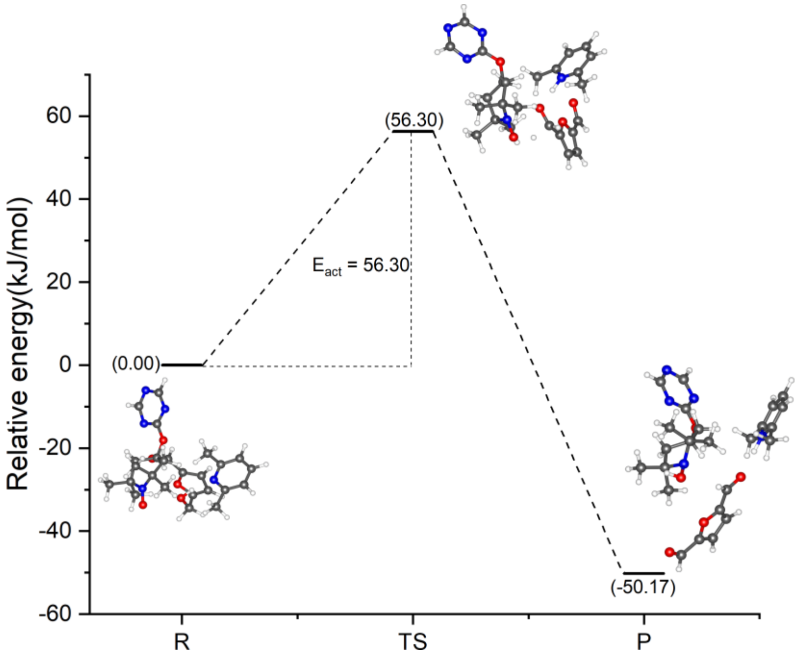

Figure S7. Relative energy profile for the conversion of HMF to DFF with **Hub[3]-TEMPO** catalyst.

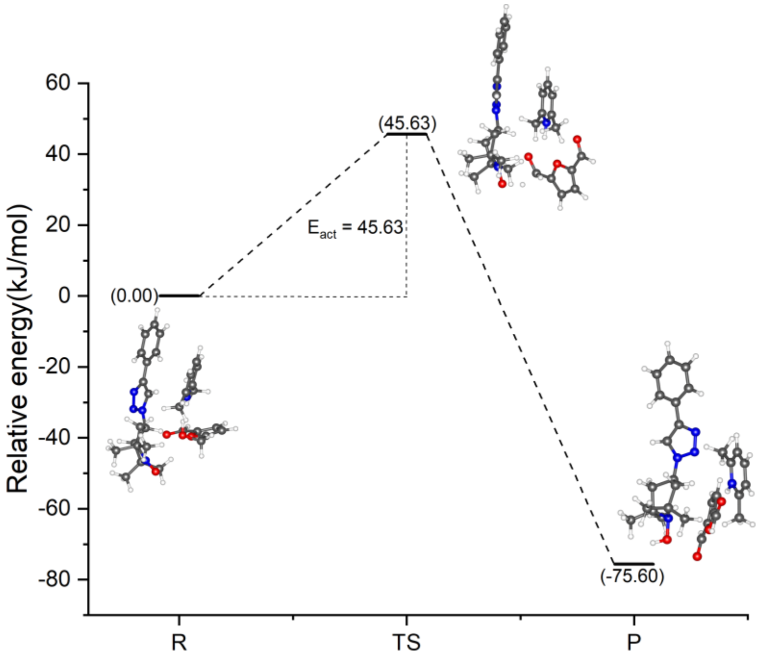

Figure S8. Relative energy profile for the conversion of HMF to DFF with **Hub^{7}-TEMPO** catalyst.

2.3. Comparison of the different C3-symmetric TEMPO derivatives

The comparison of the different suggested size-enlarged C3-symmetric TEMPO derivatives is shown in Table S2. The molecule radius and the energy profile were determined by QM calculations (see Figure S1–S8). The larger the molecule radius, the better retention can be expected on the membrane during the nanofiltration process. With lower calculated formation energy, faster reaction rates can be predicted. To ensure the stability of the mediator, labile anchor-types are unfavored. Regarding the synthesis, the reaction route should be straightforward and cost-effective. In Table S3, the actual costs of the needed chemicals are collected.

Table S2. Comparison of the different C3-symmetric TEMPO derivatives based on size, activity, stability, difficulty of synthesis and price (*green: favoured; yellow: neutral; red: unfavoured*)

Derivative	Molecule radius (Å)	Transition state (TS) relative Energy (kJ mol^{-1})	Type of compound (stability)	Difficulty of synthesis	Number of steps	Cost (USD $mmol^{-1}$)
Hub[1]-TEMPO	9.2	36.13	ether		1	20
Hub[2]-TEMPO	9.4	27.73	amine		1	101
Hub[3]-TEMPO	8.3	56.30	phenol		1	14
Hub[4]-TEMPO	8.4	75.48	aromatic amine		1	55
Hub[5]-TEMPO	9.2	64.08	ester		1	15
Hub[6]-TEMPO	9.3	63.59	amide		1	56
Hub[7]-TEMPO	10.8	45.63	triazole		3	47
Hub[8]-TEMPO	13.5	63.66	amide		1	172

Table S3. Prices of available chemicals for the synthesis of size-enlarged C3-symmetric TEMPO derivatives (Source: https://www.sigmaaldrich.com/united-states.html; 24.12.2019)

Compound name	M (g mol^{-1})	Pack size (g)	Pack size (mmol)	Price (USD)	USD/mmol
4-OH-TEMPO	172.24	1	5.81	26.3	4.5
4-NH_2-TEMPO	171.26	1	5.84	106	18.2
Benzene-1,3,5-tricarboxaldehyde	162.14	0,25	1.54	71.1	46.1
1,3,5-Benzenetricarbonyl trichloride	265.48	10	43.50	79.4	1.8
Cyanuric chloride	184.41	5	27.11	18.2	0.7
1,3,5-Tris(bromomethyl)benzene	356.88	5	14.01	84.1	6.0
1,3,5-Triethynylbenzene	150.18	0.25	1.66	55.6	33.4
1,3,5-Tris(4-carboxyphenyl)benzene	438.43	1	2.28	267	117.1

3. Methods and materials

Rhodiasolv® PolarClean HSP was purchased from Solvay, while γ-valerolactone and dimethyl sulfoxide were obtained from Alfa Aesar. Acetonitrile, hexane, propylene carbonate, and THF were bought from Merck, while acetone, anhydrous sodium carbonate, sodium chloride and hydrochloric acid were supplied by Sinopharm Chemical Reagent Co. Ltd. (Shanghai, China). Ethyl acetate was purchased from either Merck or Sinopharm. All these compounds were used without further purification. Type II Millipore water was used. TEMPO (Alfa Aesar), 4-OH-TEMPO (Merck), SiliaCAT TEMPO (Merck), TurboBeads™ TEMPO (Merck), 1,3,5-tris(bromomethyl)benzene (Fluorochem), 2,6-lutidine (Merck), HMF (Merck, Alfa Aesar, or prepared based on our previous procedure[25b]), sodium hydride, L-ascorbic acid, and LiClO4 (Merck) were used as supplied. Choline chloride (ChCl) and fructose were purchased from Aladdin Chemical Technology Co. Ltd. (Shanghai, China).

The electrochemical experiments were carried out using an IKA ElectraSyn 2.0 potentiostat equipped with either a single vial holder, or a 6-reaction carousel, or a GOGO module connected to an IKA KS 4000 i control shaker. The reactions were conducted in constant current mode, without a reference electrode. The electrodes and vials were purchased from IKA. The electrodes were washed multiple times with water, and acetone, and were rubbed dry with tissue paper before each use.

Infrared spectra were recorded on a Bruker Alpha-T FT−IR spectrometer (s: strong, m: medium, w: weak).

Electron paramagnetic resonance spectroscopy was carried out in an EPR spectrometer (Xenon series from Bruker) at room temperature, and the unit was operated in the X-Band mode with a microwave frequency of 9.4–9.8 GHz and a modulation frequency of 100 kHz. An ER 221 Bruker cell tube with an inner diameter of 3 mm and an outer diameter of 4 mm was used to load the samples. For solid state measurements, the samples were mixed with KBr powder to dilute the concentration. The sweep width was set at 600 G with a modulation amplitude of 0.4 G. The radio frequency power was set to 0.6325 mW with power attenuation of 25 dB. For solvated state measurements, the samples were solvated with acetonitrile. The sweep width was set at 8000 G with a modulation amplitude of 4 G. The radio frequency power was set to 0.6325 mW with power attenuation of 25 dB.

NMR spectra were recorded either on a Bruker DRX-500 Avance spectrometer (at 500 MHz for ^{1}H and at 125 MHz for ^{13}C spectra) or on a Bruker 300 Avance spectrometer (at 300 MHz for ^{1}H and at 75.5 MHz for ^{13}C spectra), as specified for each compound.

High resolution mass measurements were performed on a Thermo Exactive plus EMR Orbitrap mass spectrometer, which was used with a Thermo Ultimate 3000 UHPLC with 100% methanol as the mobile phase; or on a Micro-TOFII Mass Spectrometer (Bruker Daltonics) was used for determining the m/z of the studied molecule. The Micro-TOF spectrometer could reach a high resolution (> 10,000) and reliable mass accuracy (<5 ppm mass error. Electrospray ionization in positive mode (ESI+) was applied for the studied compound. The Mass spectrometer was calibrated using a purchasable "Calibration Mix ESI (Agilent)" by following the manufacturer guideline. The sample was dissolved in acetonitrile (LC-MS grade) prior to the measurement. The samples were directly infused with a Hamilton (500 µL) syringe using an independent infusion pump.

LCMS was performed using an Ultimate-3000-TM RSLCnano from thermoscientific, equipped with a UV (254 nm) diode array detector Nanospray Flex Ion source to interface with Thermo Scientific Mass Spectrometers. 1.5 m of fused silica (20 µm and 50 µm) and a tile for cutting fused silica capillaries have been included in the pump (NCS-3500RS and NCP-3200RS) accessory kits. The connection between the capillary outlet and the emitter is realized using a 1/32" micro tight® union assembly included with the Nanospray Flex Ion Source. The sample was dissolved in MeCN prior to analysis. Reversed phase LC separation was conducted by using 95% water and 5% acetonitrile in a Hypersil GOLD column (100 × 2.1 mm). During LC-MS, scans were performed only for positive ions.

Particle size was determined by means of dynamic light scattering using a Zetasizer Nanoseries instrument (Malvern Panalytical). The measurement was carried out at 25 °C with an equilibrium time of 120 s. One data collection consisted of ten runs, each with a duration of 10 s.

Melting points were recorded with a Boetius micro-melting point apparatus, and the observations were not corrected.

UV/VIS spectrum was recorded on AvaSpec-Sensline spectrometer equipped with Avalight-DHS light source and AvaSpec-ULS-RS-TEC detector. The sample was dissolved in MeCN (1 mg mL^{-1}) and was measured in absorbance mode and the results were evaluated with the AvaSoft 8.10 software.

Silica gel 60 F_{254} (Merck) plates were used for thin-layer chromatography (TLC) and the spots were visualized either by ultraviolet light (254 nm) or by staining with an acidic H_2O/EtOH solution of 2,4-dinitrophenylhydrazine (DNP). Silica gel 60 (70−230 mesh, Merck) was used for column chromatography. The ratios of the solvents for the eluents are given in terms of volume (mL mL^{-1}).

Yields (except for isolated yields) of the electrochemical oxidation reactions and the purity of the recovered Hub^1-TEMPO were determined based on the LC-(UV)-MS chromatograms. Samples were dissolved in acetonitrile prior to analysis. LC-(UV)-MS was performed using an Ultimate-3000-TM RSLCnano from thermoscientific, equipped with a UV (254 nm) diode array detector Nanospray Flex Ion source to interface with Thermo Scientific Mass Spectrometers. 1.5 m of fused silica (20 µm and 50 µm) and a tile for cutting fused silica capillaries have been included in the pump (NCS-3500RS and NCP-3200RS) accessory kits. The connection between the capillary outlet and the emitter is realized using a 1/32" micro tight® union assembly included with the Nanospray Flex Ion Source. Reversed phase LC separation was conducted by using 95% water and 5% acetonitrile in a Hypersil GOLD column (100 × 2.1 mm). During LC-MS, scans were performed only for positive ions. HMF and DFF were analyzed quantitatively using a UV detector set at 265 nm and an xBridge Shield RP18 3.5 µm C18 column (250 × 4.6 mm). The eluent comprised of 5 mM NH_4HCO_2 aqueous solution and methanol solvent in channels A and B, respectively. The resolution of HMF and DFF was achieved with isocratic elution of 70% A and 30% B for 10 min run time and the flow rate was set at 1 mL min^{-1}. The identification and quantification of compounds were determined from the calibration curves by applying standard solutions with known concentrations of pure compounds.

4. TEMPO-mediated electrocatalytic oxidation – additional information

4.1. Schematic representation of the applied TEMPOs in the oxidation of HMF to DFF

For the electrocatalytic oxidation of HMF to DFF both homogeneous and heterogeneous TEMPO catalysts have been investigated (Figure S9). The recovery of the soluble TEMPOs

(TEMPO, **Hub[1]-TEMPO**) was attempted by nanofiltration, while the solid TEMPOs (SiliaCAT, TurboBeads) were separated by filtration and by using magnet, respectively.

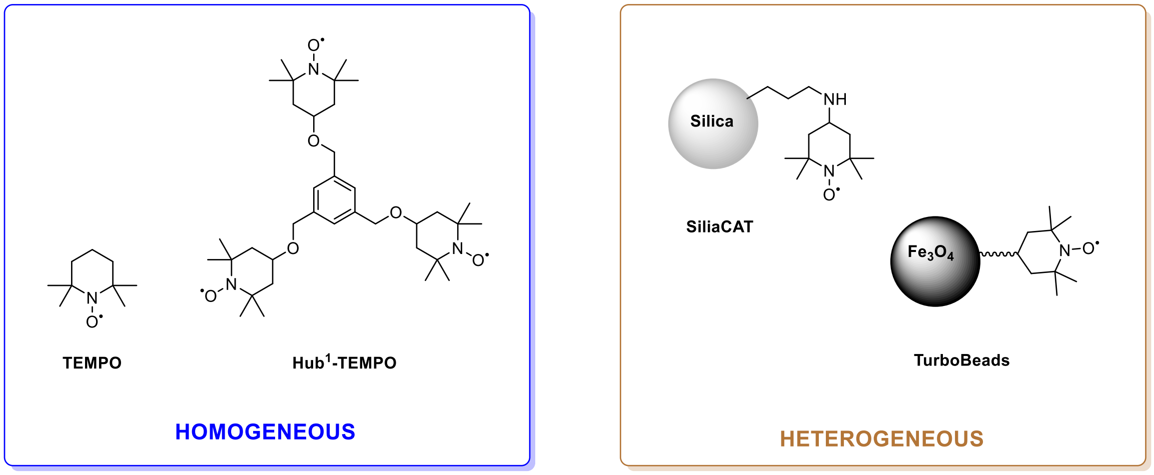

Figure S9. Schematic representation of the applied homogeneous (blue) and heterogeneous (brown) TEMPOs in the oxidation of HMF to DFF

4.2. *Calculation method of the conversion and the yield*

The conversion was calculated based on peak areas in the HPLC spectra. First the calibration curve of HMF and DFF was determined by linear regression (Figure S10). Using Equation S1, the concentration of the product (C_P) can be calculated.

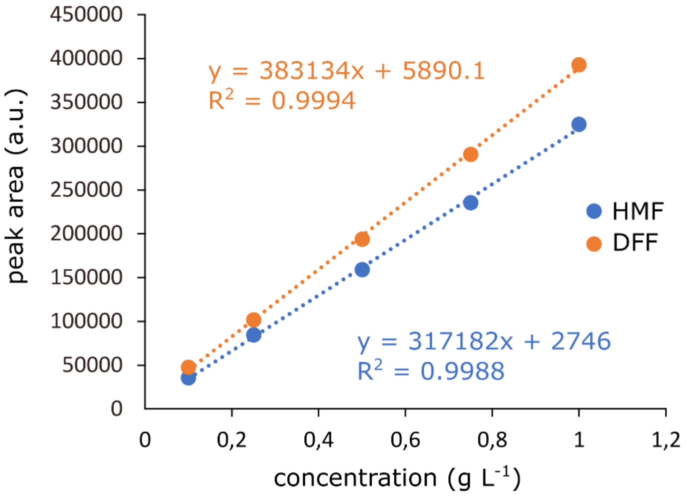

Figure S10. HPLC calibration for HMF and DFF.

The conversion was calculated by Equation S2, where A_{SM} is the peak area of the starting material (HMF) and A'_P is the corrected peak area of the product (DFF) calculated from the calibration equation of the starting material using Equation S3.

Eq S1. $$C_P = \frac{A_P - 5890.1}{383134}$$

Eq S2. $$conversion\ (\%) = \frac{A'_P}{A_{SM} + A'_P} \cdot 100$$

Eq S3. $$A'_P = 317182 \cdot C_P + 2746$$

The yield of the reaction was calculated based on the mass balance. First, the concentration (mmol L^{-1}) of the starting material and the product were calculated. The selectivity can be decided from Equation S4. Finally, the yield is determined by multiplying the conversion with the selectivity (Equation S5). For the most part, the yield is given as the average result of three parallel experiments. In these cases, the error is also presented.

Eq S4. $$selectivity = \frac{C_{SM}^N + C_P^N}{C_{SM}^1 + C_P^1}$$, where N: sample number

Eq S5. $$yield\ (\%) = selectivity \cdot conversion$$

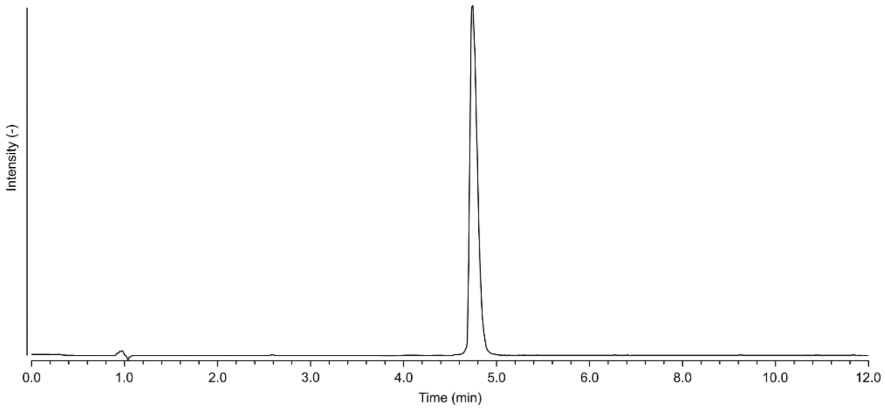

Figure S11. HPLC chromatogram for HMF.

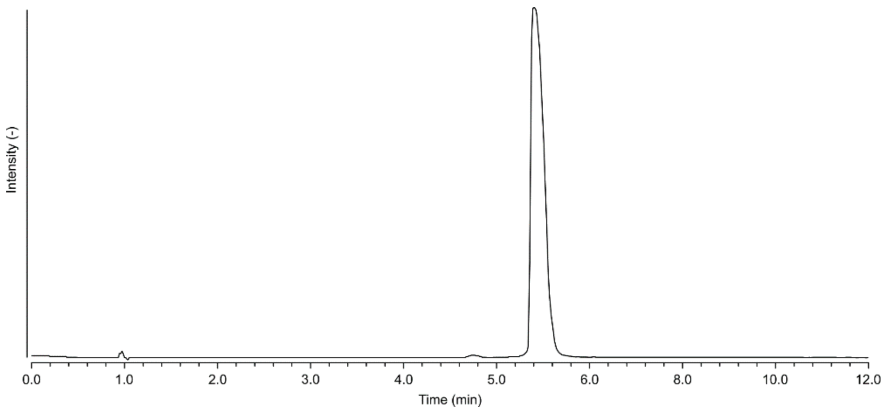

Figure S12. HPLC chromatogram for DFF.

4.3. RVC vs Graphite electrode

By changing the anode material from graphite to RVC, the electrocatalytic oxidation of HMF was slightly faster, although after 16 h both electrode types provided full conversion (Figure S13). Considering their price (graphite SK-50, set of 12: EUR 148.00 *vs* RVC, set of 12: EUR 251.00)[13], and the more robust structure of the graphite electrode, application of RVC electrode was considered disadvantageous.

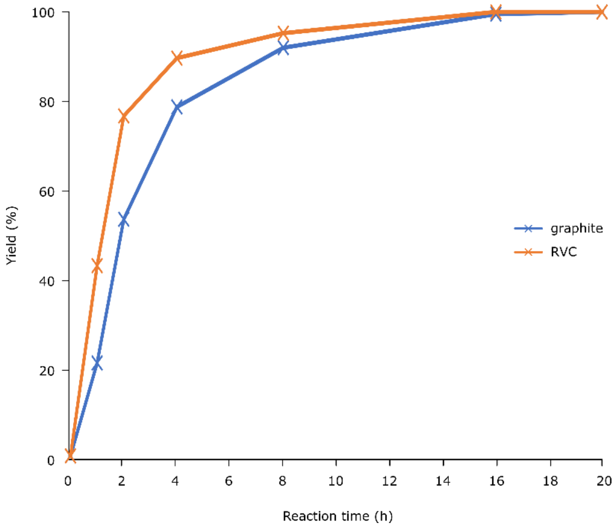

Figure S13. The effect of anode material on the electrooxidation of HMF into DFF.

4.4. Amount of TEMPO mediator

The effect of applied mediator amount was also investigated. Accordingly, the electrooxidation was performed with 20%, and 30% mol% TEMPO mediator as well (Figure S14). Increasing the concentration of TEMPO in the reaction mixture showed no significant impact on the reaction rate.

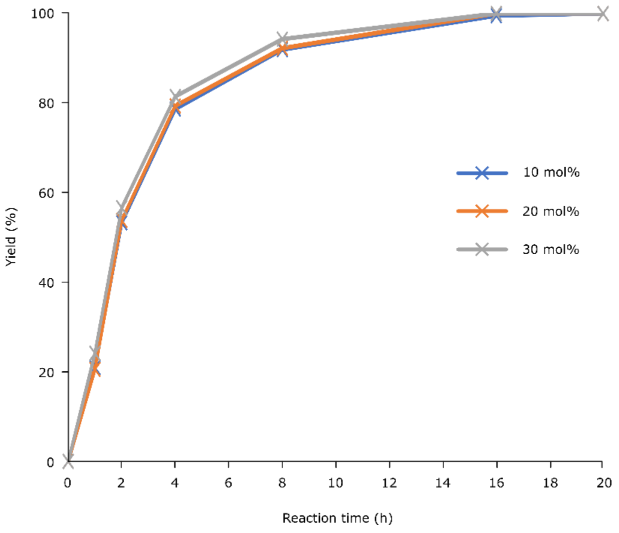

Figure S14. The effects of mediator mol% on the electrooxidation of HMF into DFF.

4.5. Constant current electrooxidation at 5 mA

During the examination of current strength upon the reaction rate, deformation of the electrodes was experienced at 5 mA constant electrolysis. After the first hour appearance of dark brown precipitation was observed. After 16 h, the potential reached 10V while the electrodes started to show deformation (Figure S15). The experiment was discontinued at this point.

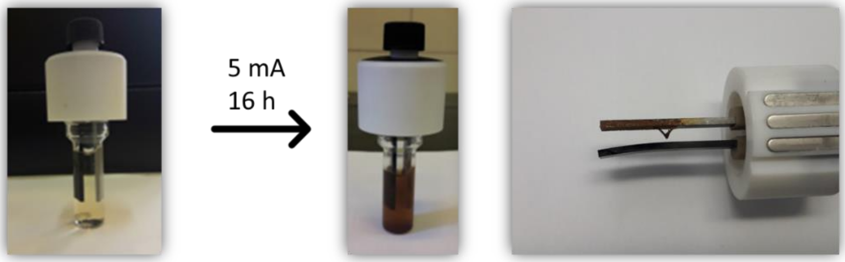

Figure S15. Electrode deformation experienced at 5 mA constant current electrolysis.

4.6. Kinetic studies

To determine the order of reaction rate, samples were taken hourly from the reaction mixtures for the different TEMPO mediated oxidation reactions and analyzed by HPLC. Since the concentration of the mediator remains constant and the substrate is the limiting species, the reaction can be assumed to follow pseudo-first order kinetics.[14] Consequently, the concentration of the mediator can be included in the rate constant (k'). The total reaction rate is described in Equation S6, and the application of $t = 0$ and $C_{substrate} = C^0_{substrate}$ boundary conditions gives Equation S7.

Eq S6. $$r = -r_{substrate} = -\frac{dC_{substrate}}{dt} = k'C_{substrate}$$

Eq S7. $$lnC_{substrate} = -k't + lnC^0_{substrate}$$

where r is the total reaction rate, k' is the pseudo-first-order reaction rate coefficient (in h^{-1}), and C is the concentration of substrate (in mol L^{-1}). With the transformation of Equation S7, we can analyze the linear correlation between $ln(\frac{C_{substrate}}{C^0_{substrate}})$ and t (Equation S9).

Eq S8. $$lnC_{substrate} - lnC^0_{substrate} = -k't$$

Eq 4. $$ln(\frac{C_{substrate}}{C^0_{substrate}}) = -k't$$

The experimental results of the solid supported TEMPOs, namely TurboBeads (Figure S18) and SiliaCAT (Figure S19), show excellent correlation with the pseudo-first order kinetic model that validate the assumed linear relationship between $ln(\frac{C_{substrate}}{C^0_{substrate}})$ and t. On the contrary, the homogeneous TEMPOs catalyzed reactions gave loose-fitting accordance with the assumed linear correlation (Figure S16 and S17). Therefore, we can assume that under homogeneous conditions multiple parallel processes could take place. The calculated rate coefficients (k') and the corresponding coefficients of determination (R^2) are collected in Table S4.

Table S4. Calculated rate coefficients (*k'*) and coefficients of determination (R^2) in the kinetic study

Catalyst	*k'*	R^2
TEMPO	0.3116	0.9618
Hub[1]-TEMPO	0.3654	0.9782
TurboBeads	0.1868	0.9994
SiliaCAT	0.1335	0.9906

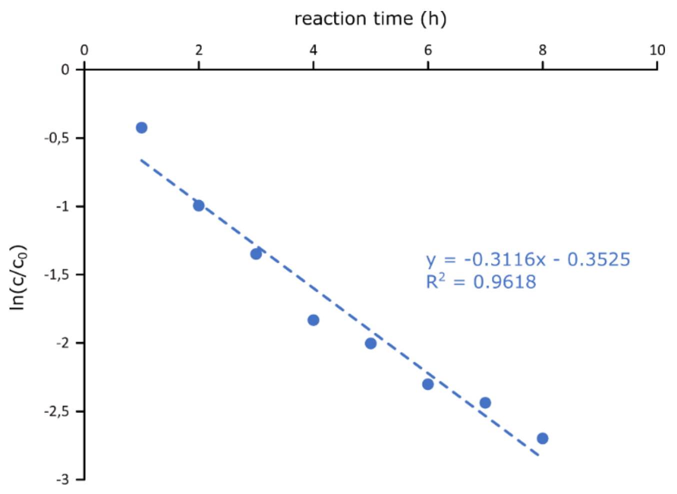

Figure S16. Kinetic study of TEMPO mediated electrochemical oxidation of HMF.

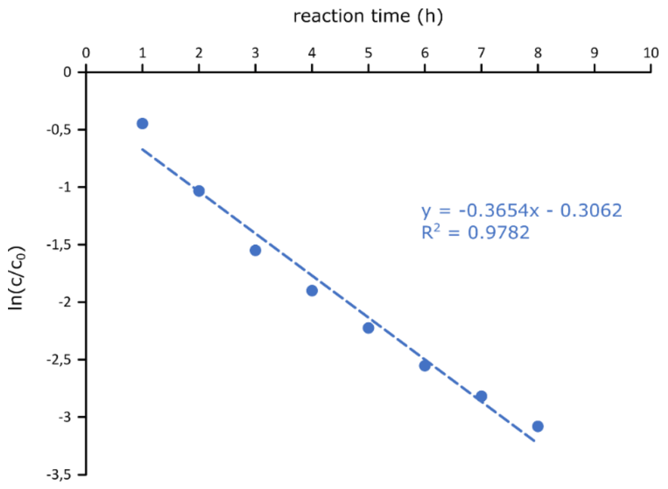

Figure S17. Kinetic study of **Hub[1]-TEMPO** mediated electrochemical oxidation of HMF.

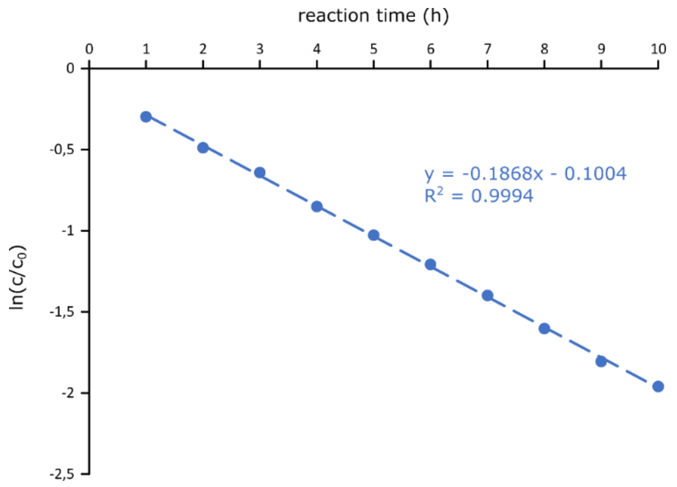

Figure S18. Kinetic study of TurboBeads mediated electrochemical oxidation of HMF.

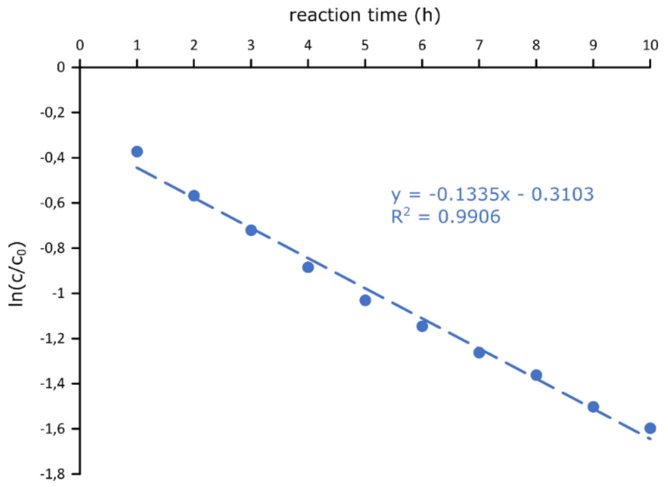

Figure S19. Kinetic study of SiliaCAT mediated electrochemical oxidation of HMF.

5. Structure elucidation of C3-symmetric *tris*-TEMPO derivative (Hub[1]-TEMPO)

*5.1. Characterization of **Hub[1]-TEMPO***

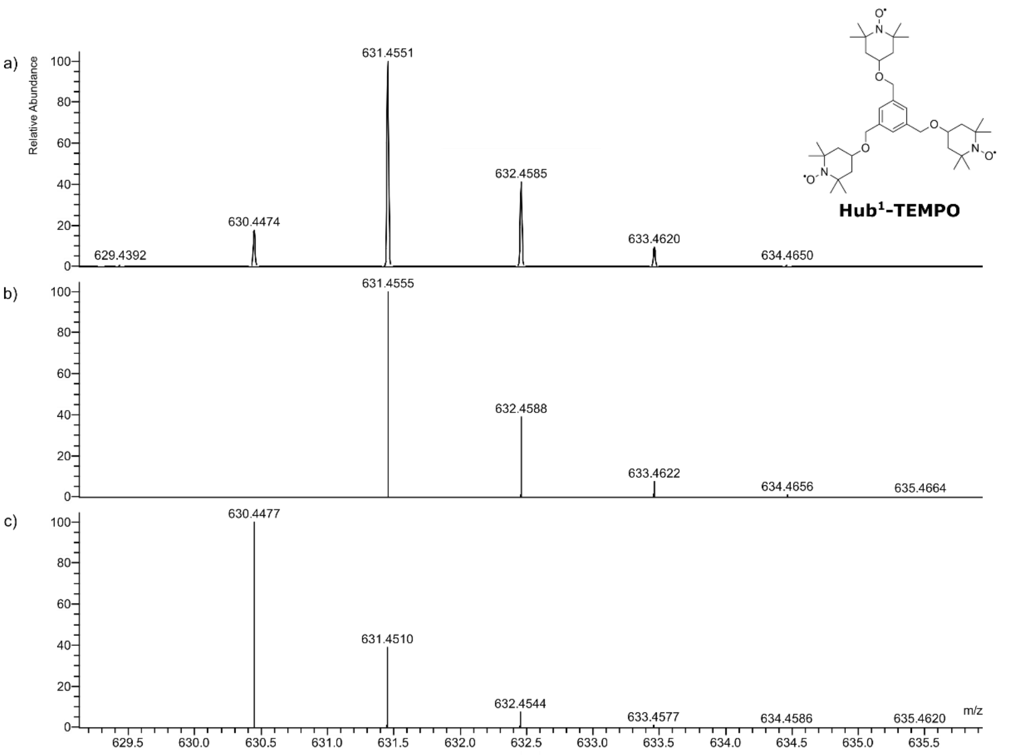

Figure S20. (a) Observed ASAP+ HRMS of **Hub[1]-TEMPO**, (b) calculated isotopic distribution of the $[M + H]^+$ ion of **Hub[1]-TEMPO**, and (c) calculated isotopic distribution of the $[M]^+$ ion of **Hub[1]-TEMPO**

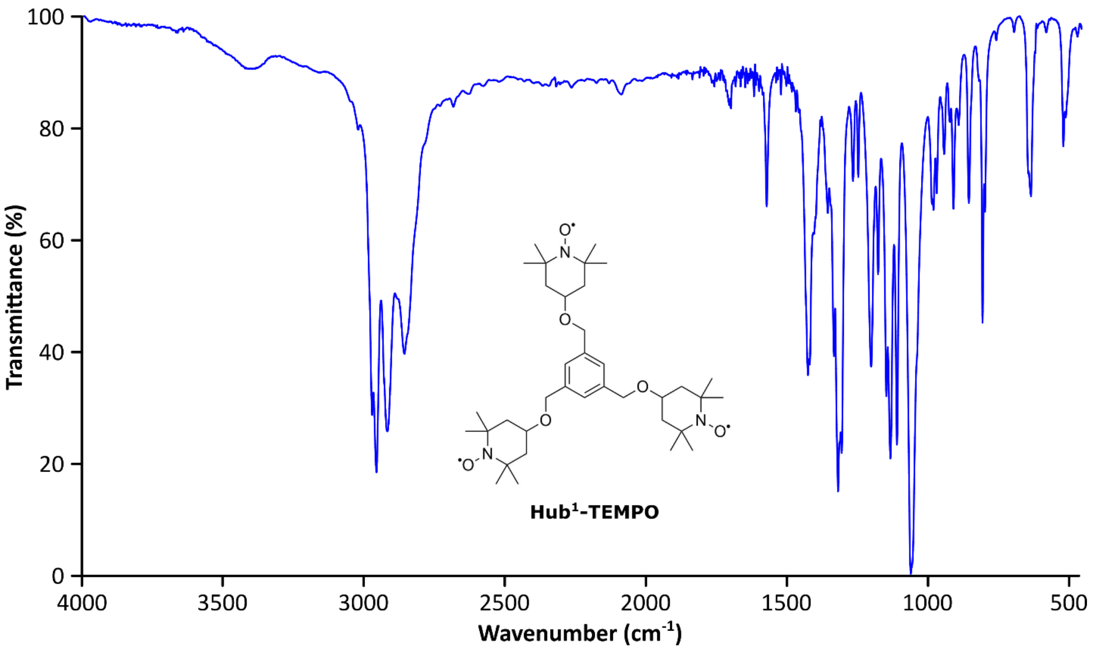

Figure S21. Infrared spectrum of **Hub¹-TEMPO** (KBr)

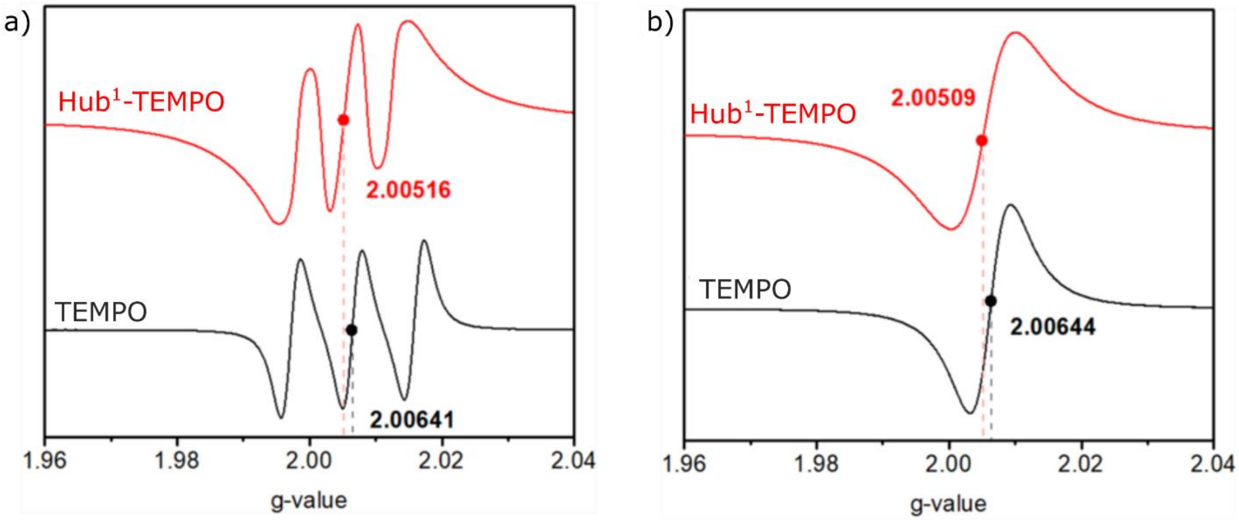

Figure S22. EPR spectra of pristine TEMPO (black) and TEMPO derivative (red) in solvated state (a), and solid state (b).

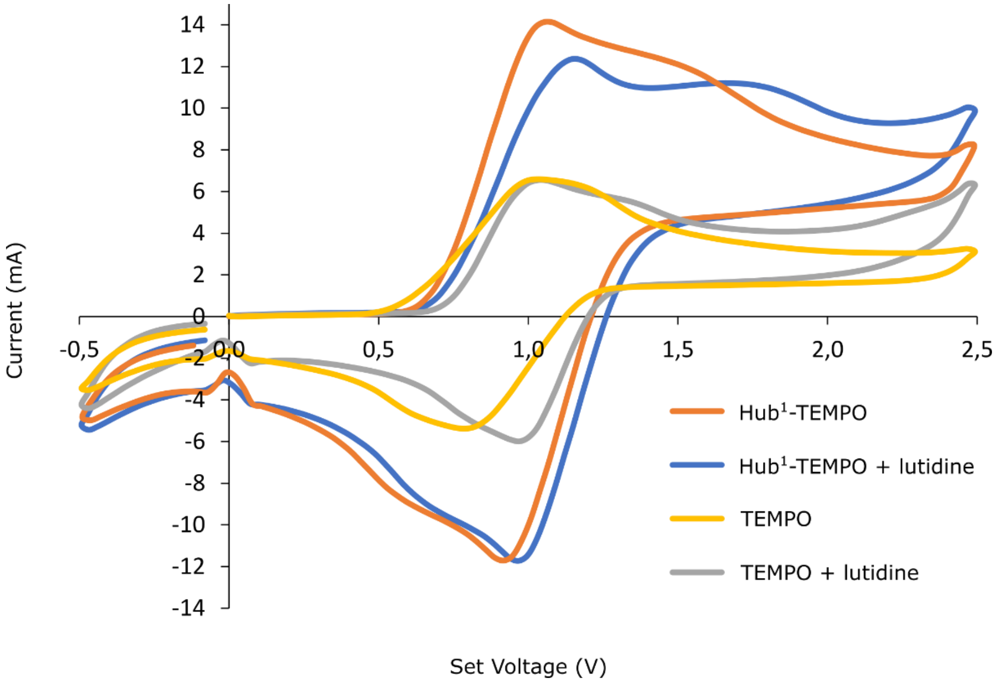

Figure S23. CVs of **TEMPO** and **Hub^{1}-TEMPO** with or without lutidine additive. Electrodes: glassy carbon (WE) and Pt (CE), with Ag/AgCl reference electrode (ElectraSyn CV package); Segments: 3; Initial V: 0; Direction: rising; Upper V: 2.5; Lower V: -0.5; Final V: 0; Sweep (mV s^{-1}): 200)

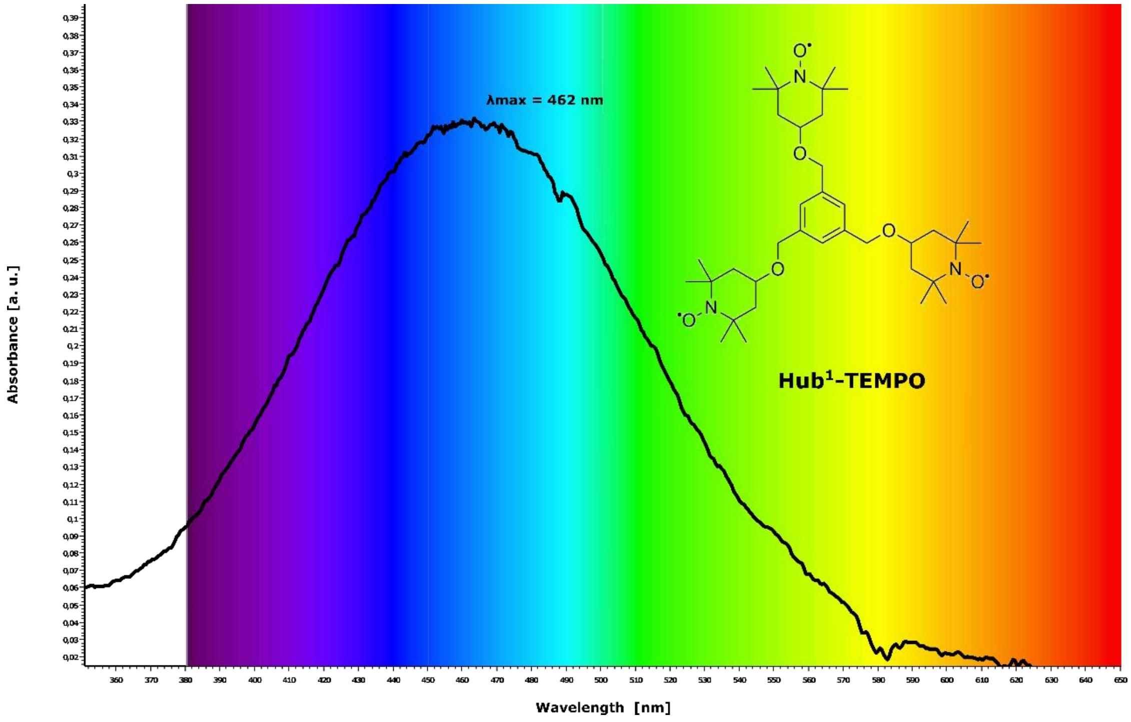

Figure S24. UV/VIS spectra of **Hub^{1}-TEMPO** (MeCN, 7.5 mg mL^{-1})

5.2. *Reduction of **Hub¹-TEMPO** to N-OH derivative, characterization of **S1***

The molecular structure of the C3-symmetric *tris*-TEMPO derivative (**Hub[1]-TEMPO**) was intended to be determined by transformation into the N-OH form. Compound **Hub[1]-TEMPO** therefore was reduced using L-ascorbic acid and the structure of **S1** was determined by HRMS, NMR and IR spectroscopy (Scheme S9). Due to the analytical results, for the structure of **S1** a monoradical form can be proposed. We can assume, that by reducing the **Hub[1]-TEMPO** no significant changes in the bond-matrix (and as a result in NMR chemical shifts) will occur. Therefore, the structure determined for **S1** corresponds to the structure of **Hub[1]-TEMPO** as well.

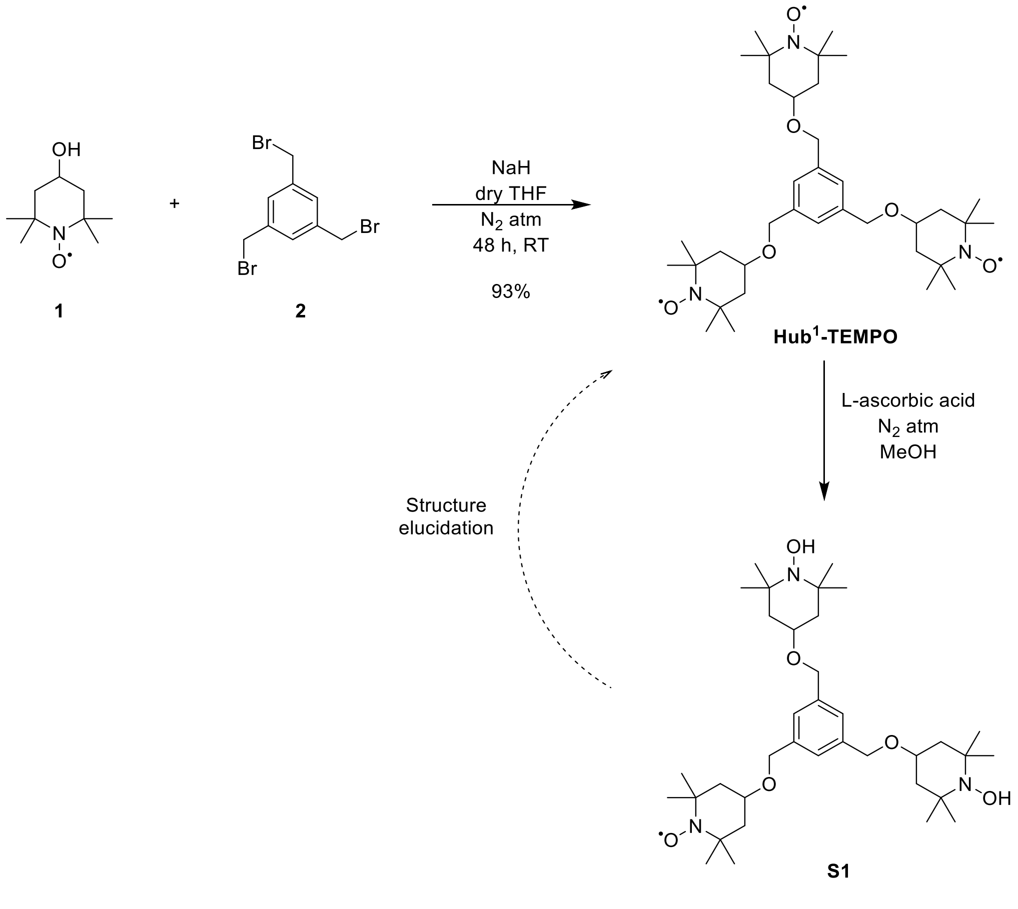

Scheme S9. Preparation of sized-enlarged TEMPO derivative **Hub[1]-TEMPO** and its reduction into **S1** for structure elucidation.

Reduced Hub¹-TEMPO (**S1**)

The title compound was prepared based on an analogous procedure.[15] Starting material **Hub¹-TEMPO** (100 mg, 0.16 mmol, 1 eq) was dissolved in MeOH (1 mL). The orange solution was deoxygenated with N_2, then L-ascorbic acid was added (102 mg, 0.58 mmol, 3.6 eq). After stirring at RT for 5 minutes, the reaction mixture turned to light yellow. Stirring was continued for 1 h, then the solvent was evaporated under reduced pressure. The remaining solid was taken up in DCM (10 mL), was washed three times with water (5 mL), the organic phase was dried over anhydrous $MgSO_4$ and concentrated in vacuo to give crude **S1** (93 mg, 92%) as a white solid. The product was submitted for analysis without further purification.

^{1}H NMR (500 MHz, $CDCl_3$): δ_H 7.23 (3H, s, ArH), 5.50 (2H, broad s, *N*-OH), 4.54 (6H, s, CH_2), 3.75 (3H, m, CH), 2.04 (6H, m, CH_2), 1.66 (6H, m, CH_2), 1.32 (18H, s, CH_3), 1.21 (18H, s, CH_3) ppm

^{13}C NMR (125 MHz, $CDCl_3$): δ_c 139.1 (3C, Ar), 125.8 (3C, Ar), 70.1 (3C, CH_2, *O*-alkyl), 60.9 (3C, CH, *O*-alkyl), 44.3, 31.4, 21.2 ppm

IR (KBr) ν_{max}: 3436 (m), 2974 (s), 2938 (s), 1459 (m), 1373 (m), 1360 (s), 1308 (w), 1245 (m), 1220 (w), 1194 (m), 1174 (m), 1085 (s), 957 (w), 896 (w) cm^{-1}

Mp: 48–51 °C

HRMS-ESI$^+$ (m/z): $[M]^+$ calcd for $C_{36}H_{62}N_3O_6$: 632.46331, found: 632.46124

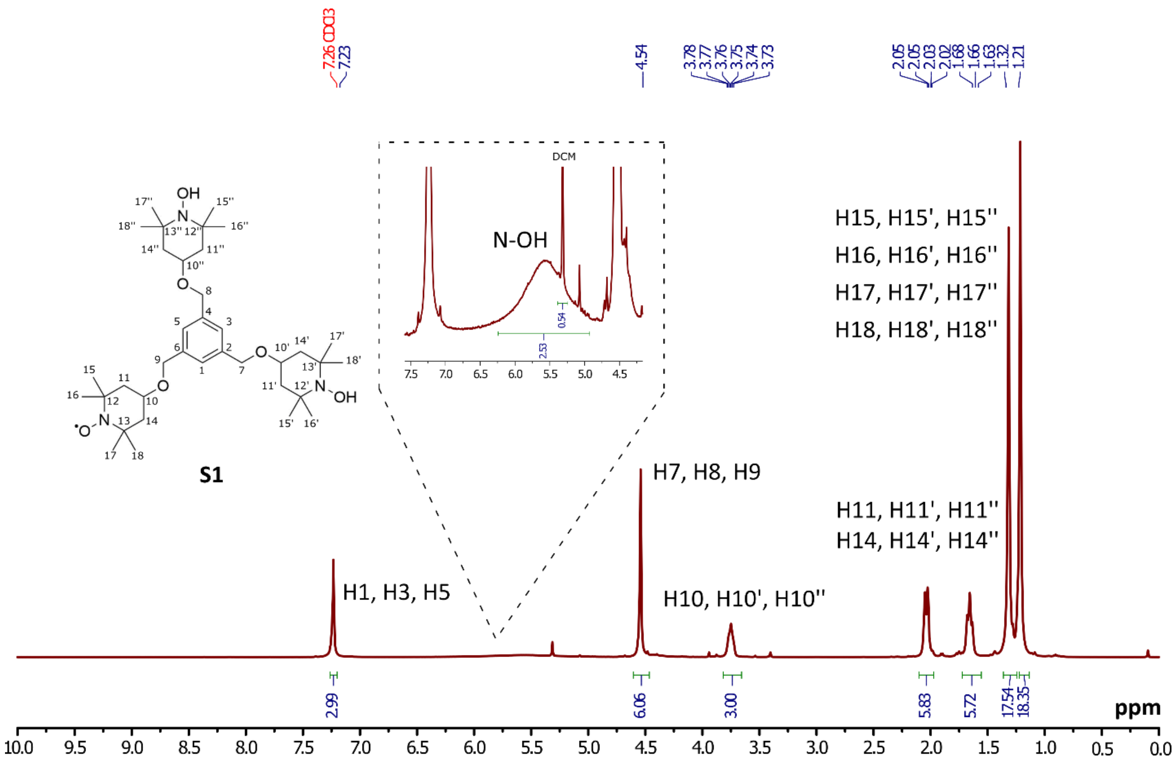

Figure S25. ^{1}H NMR spectrum of **S1** ($CDCl_3$, 500 MHz)

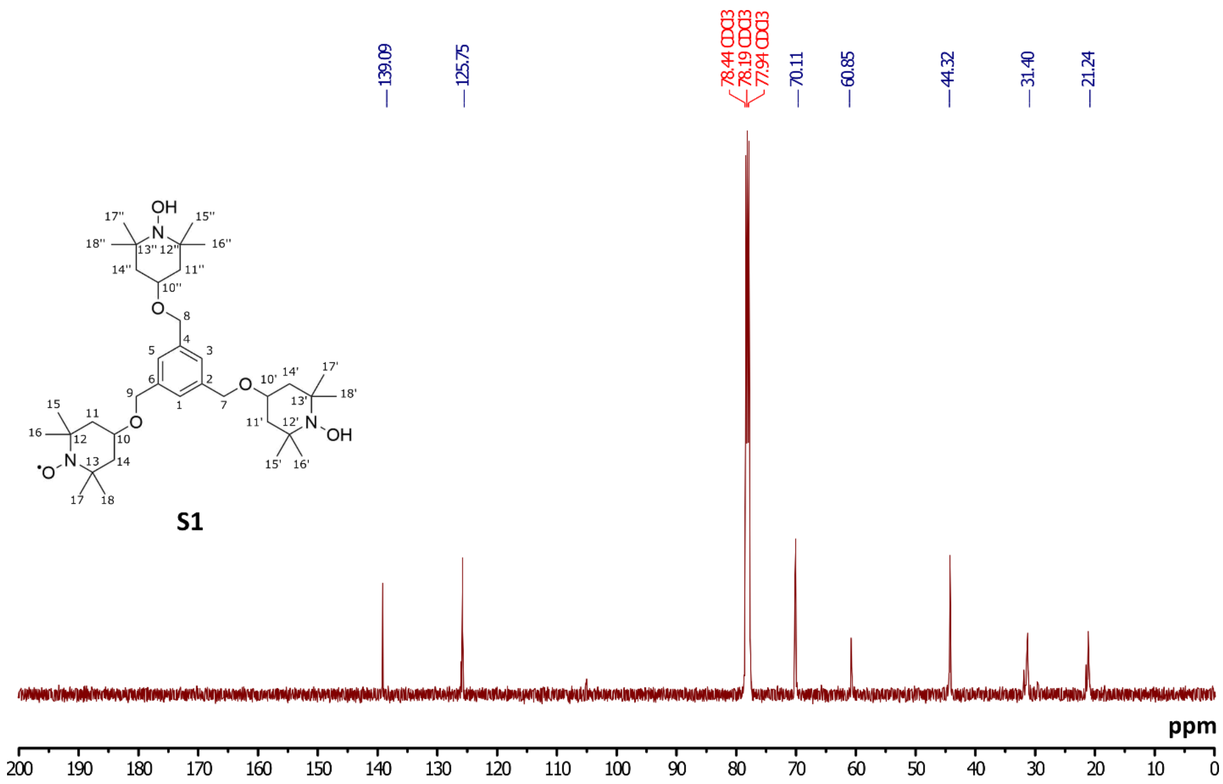

Figure S26. ^{13}C NMR spectrum of **S1** ($CDCl_3$, 125 MHz)

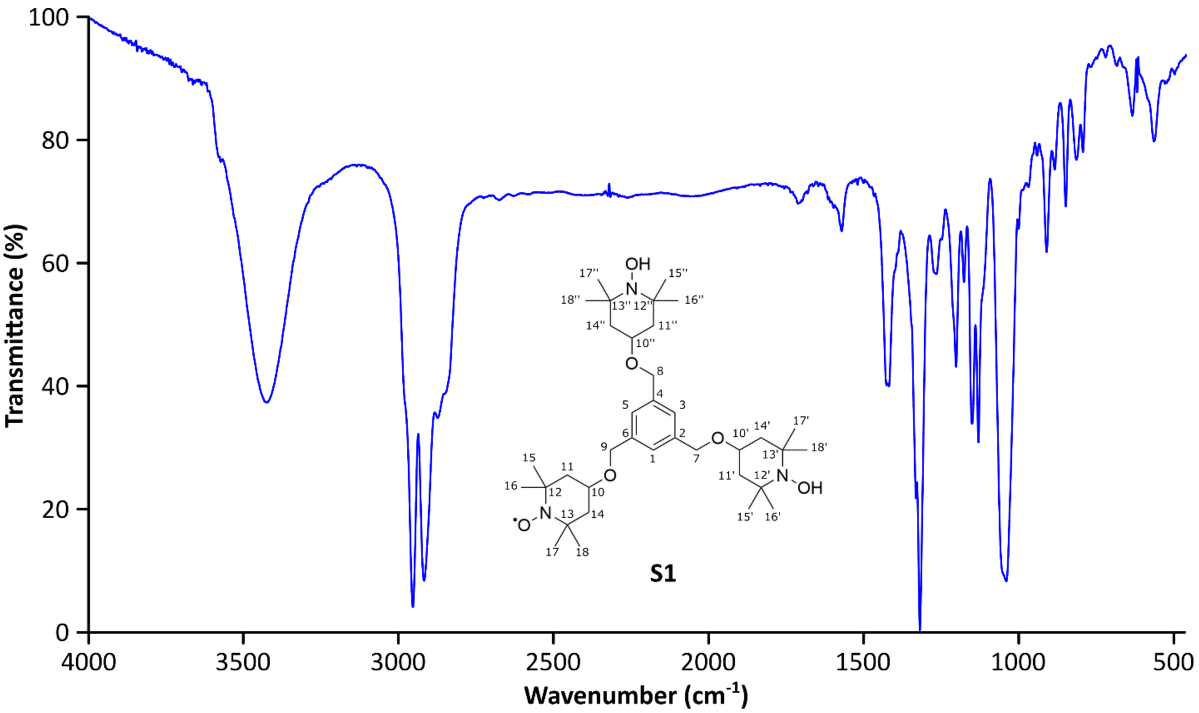

Figure S27. Infrared spectrum of **S1** (KBr)

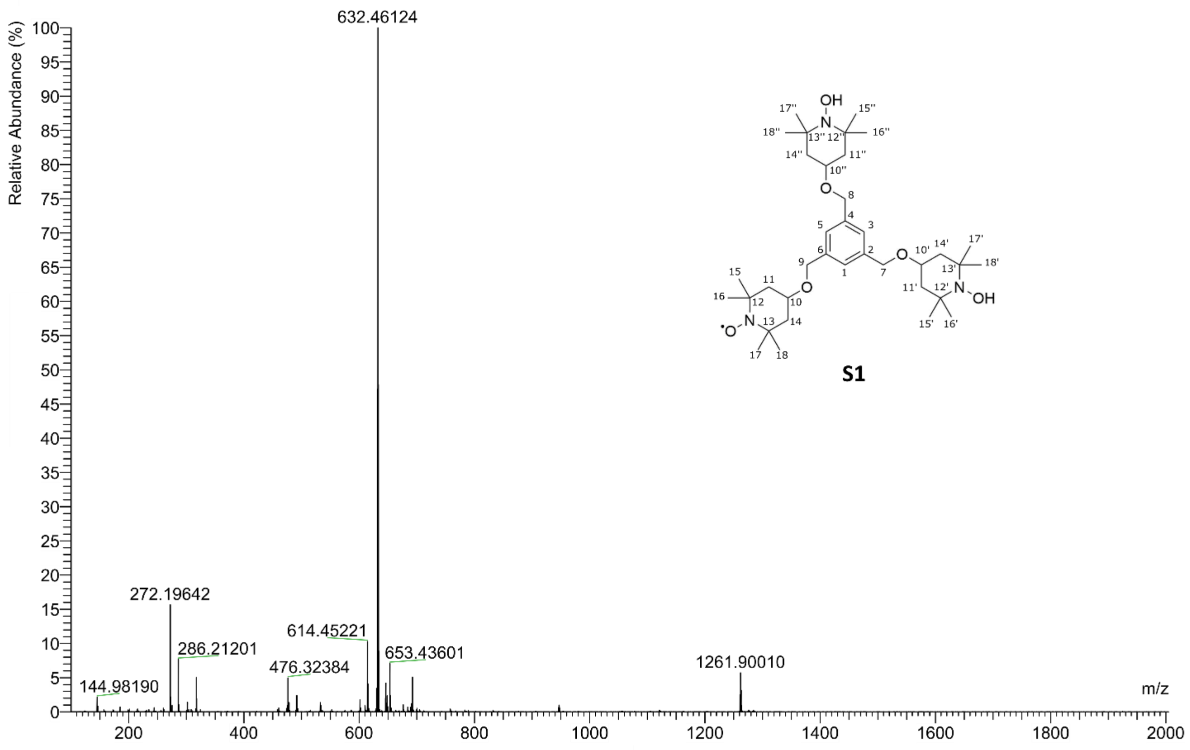

Figure S28. Full observed ESI+ HRMS of **S1**

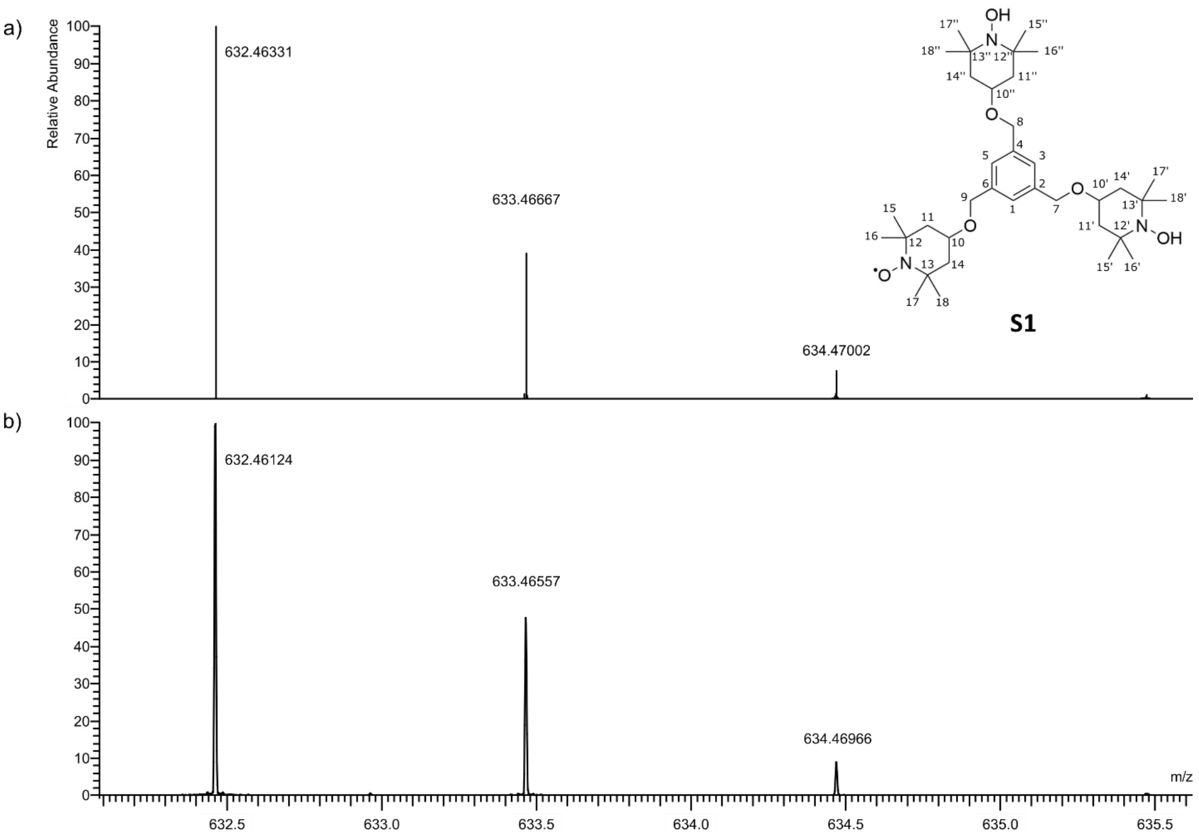

Figure S29. (a) Calculated isotopic distribution of the [M]+ ion of **S1**, (b) Observed ESI+ HRMS of **S1**

6. DFF characterization

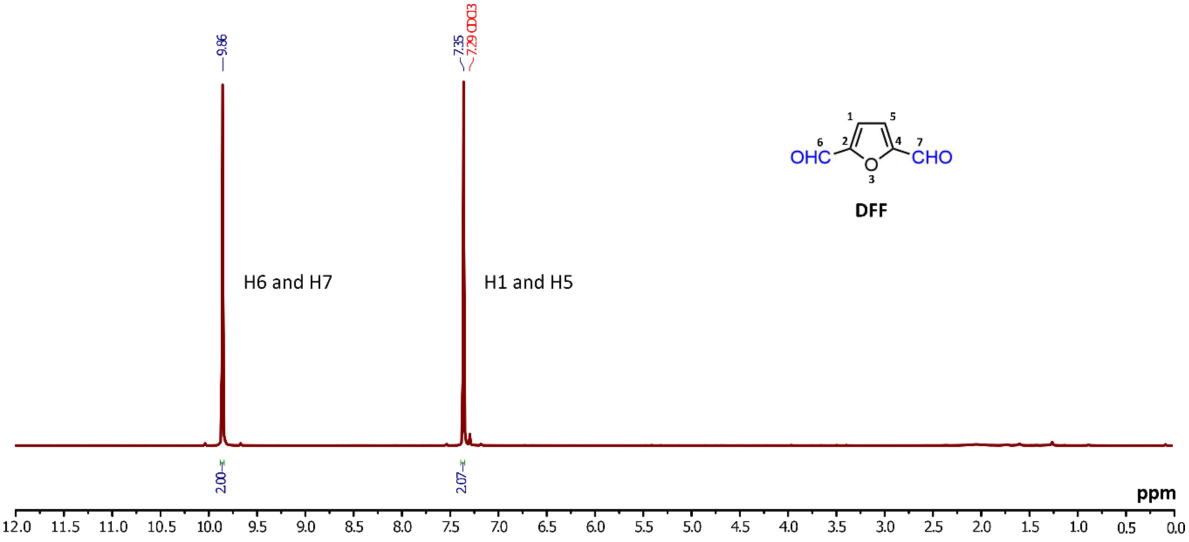

Figure S30. ^{1}H NMR spectrum of DFF ($CDCl_3$, 500 MHz)

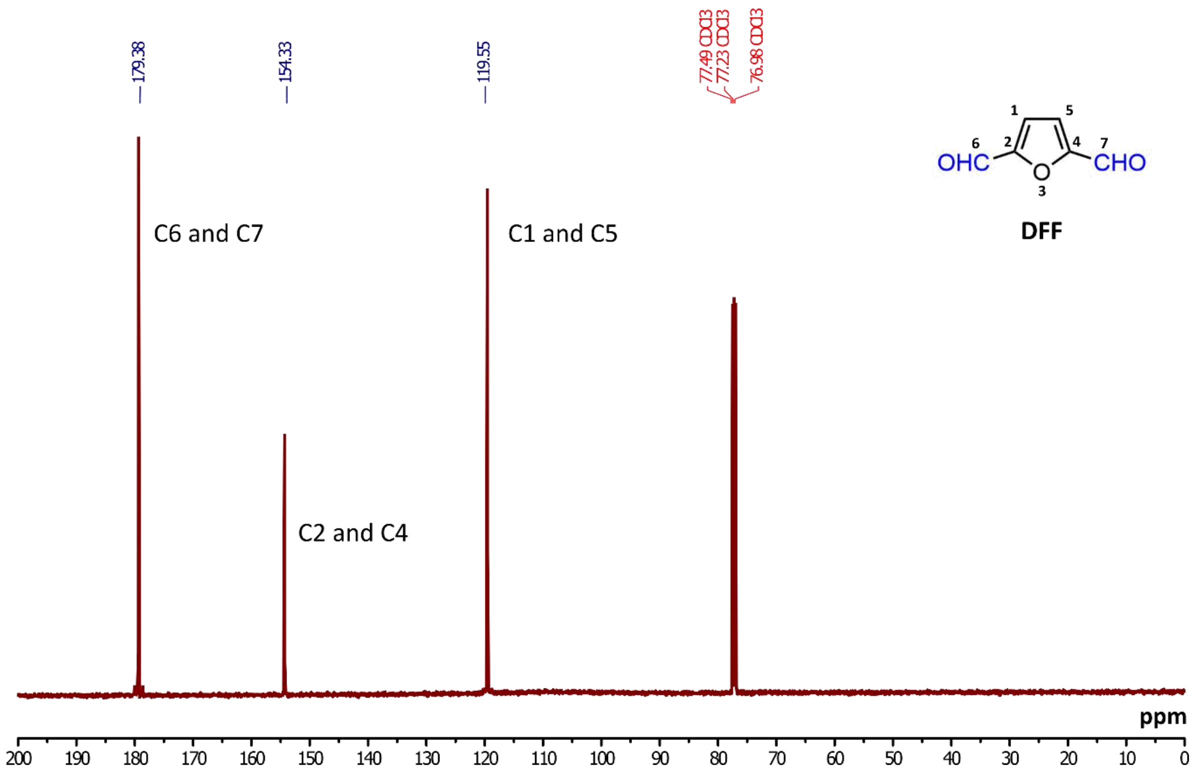

Figure S31. ^{13}C NMR spectrum of DFF ($CDCl_3$, 125 MHz)

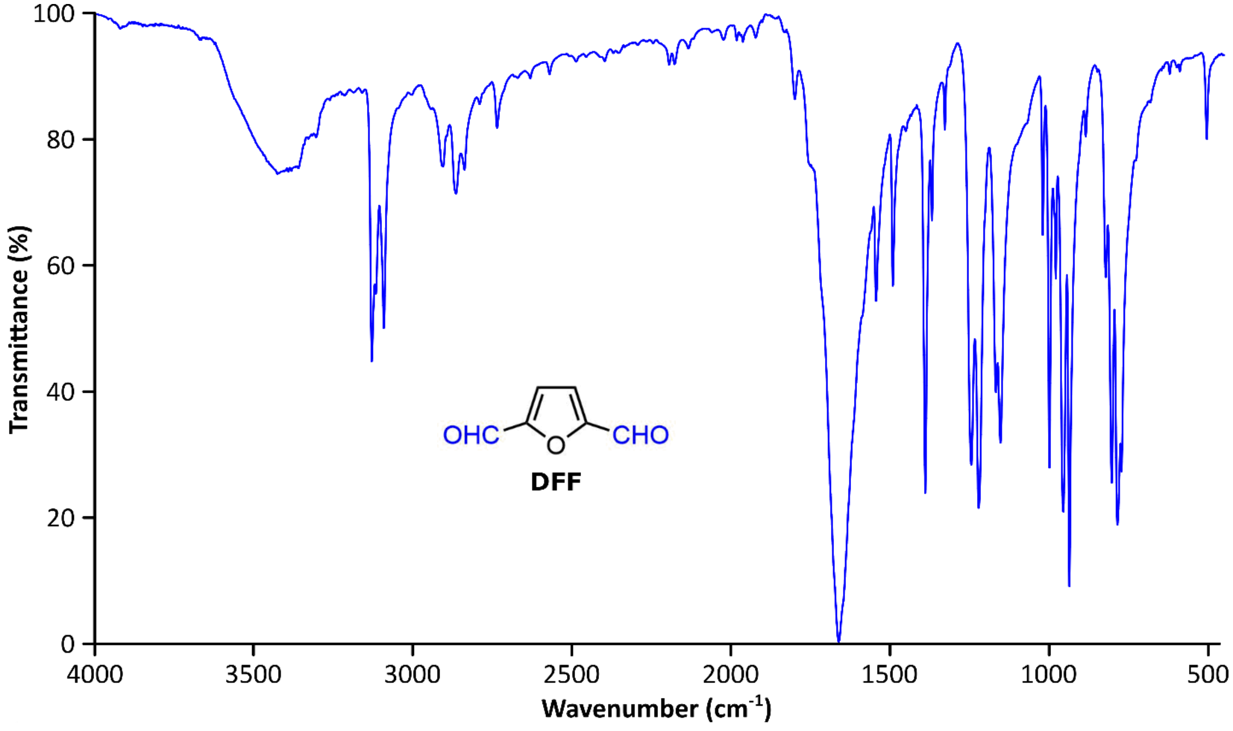

Figure S32. Infrared spectrum of DFF (KBr)

7. Hub[1]-TEMPO recovery

Following the OSN experiments, **Hub[1]-TEMPO** was reanalyzed by LC and HRMS to validate its stability. The recovered catalyst showed no significant change after application.

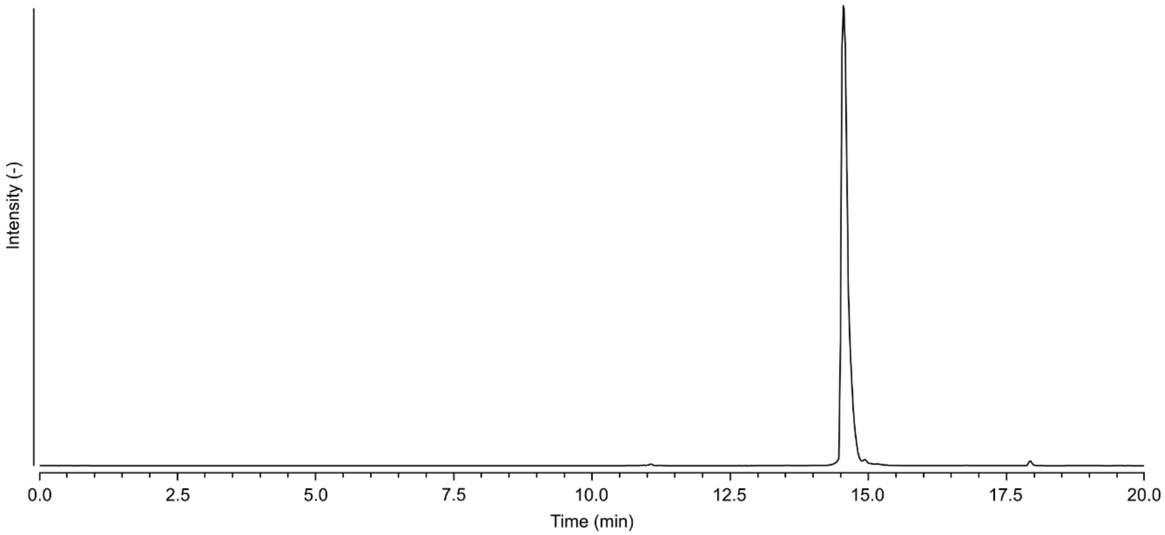

Figure S33. HPLC chromatogram of the recovered **Hub[1]-TEMPO**.

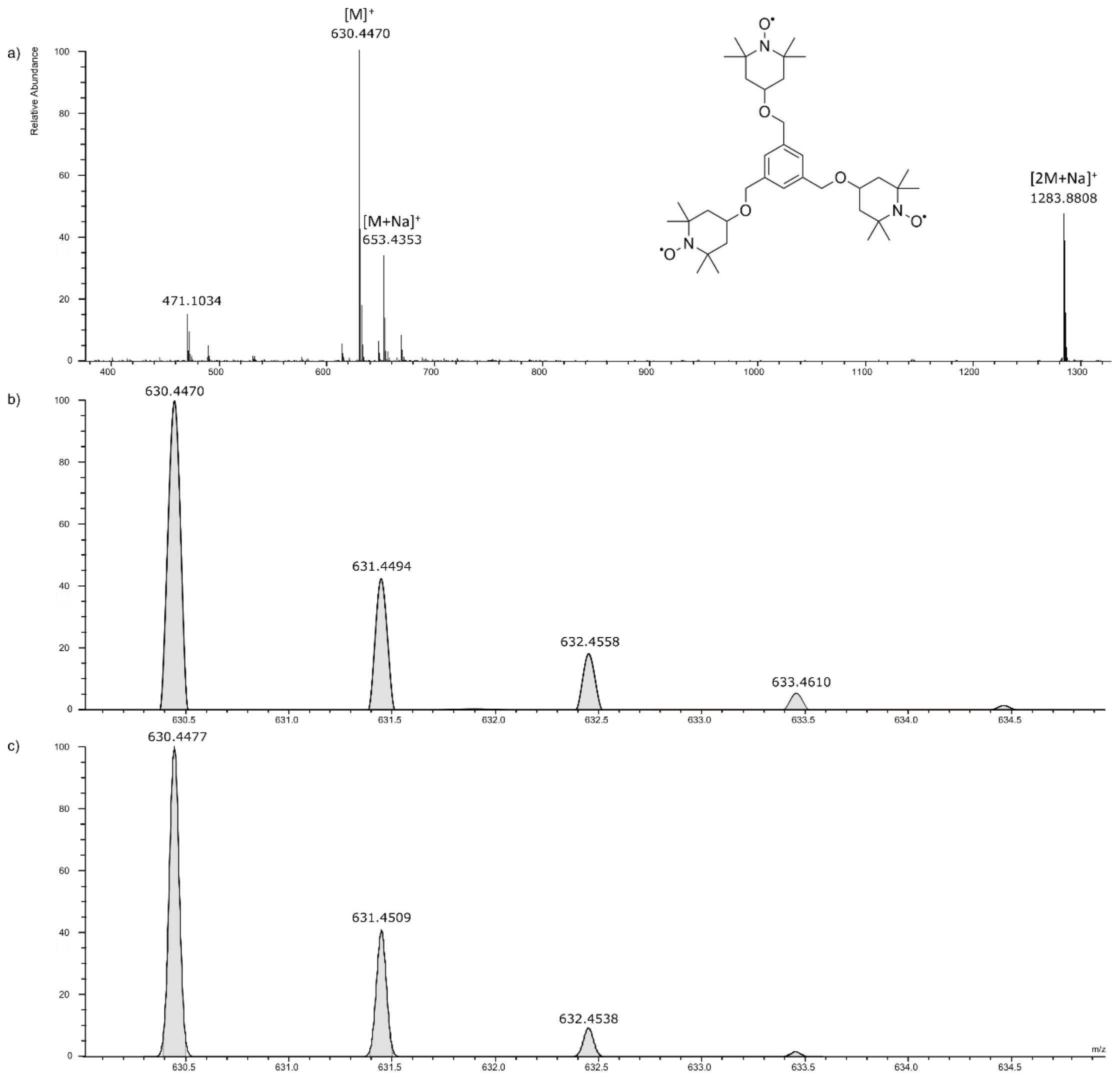

Figure S34. (a) Full observed ESI+ HRMS of **Hub[1]-TEMPO**, (b) calculated isotopic distribution of the [M]+ ion of **Hub[1]-TEMPO**, (c) Observed ESI+ HRMS of **Hub[1]-TEMPO**

8. Particle size determination of SiliaCAT TEMPO

The particle size of SiliaCAT TEMPO was determined by means of dynamic light scattering (DLS) method. The sample was dispersed in deionized water inside a glass cuvette cell with square aperture and measured immediately after the dispersion was gently shake. The measurement was carried out at 25 °C with 120 s equilibrium time. 10 number of runs with the duration of 10 s for each run were performed for 6 times data collections.

Table S5. Particle size determination of SiliaCAT TEMPO.

Number of measurements	Z-average (nm)
1	1587
2	1137
3	1362
4	996
5	1116
6	1136
**Average particle size (nm)**	**1222**

9. QM calculation coordinates

XYZ coordinates and total energies of substituted TEMPO catalyst in acetonitrile solvent.

Hub[1]-TEMPO (-2021.59538729) **Hub[2]-TEMPO** (-1962.01262158)

C	7.291000	-1.159000	-0.511000	C	1.076000	6.836000	1.306000
C	5.870000	-0.820000	-0.962000	C	0.496000	5.423000	1.247000
C	4.866000	-1.994000	-0.976000	C	-0.372000	5.100000	0.011000
C	5.252000	-3.122000	-0.017000	C	-0.007000	5.975000	-1.200000
C	5.995000	-2.681000	1.243000	C	1.455000	6.410000	-1.286000
H	5.473000	-0.030000	-0.315000	H	1.318000	4.701000	1.300000
H	4.332000	-3.615000	0.310000	H	-0.220000	5.431000	-2.126000
C	5.181000	-1.699000	2.109000	C	2.443000	5.230000	-1.387000
H	4.325000	-2.264000	2.488000	H	2.220000	4.723000	-2.330000
H	5.781000	-1.353000	2.954000	H	3.470000	5.602000	-1.416000
H	4.798000	-0.850000	1.545000	H	2.322000	4.503000	-0.584000
C	6.473000	-3.870000	2.070000	C	1.687000	7.407000	-2.419000
H	6.952000	-3.550000	2.996000	H	2.745000	7.647000	-2.531000
H	5.594000	-4.467000	2.321000	H	1.336000	6.940000	-3.342000
H	7.165000	-4.493000	1.497000	H	1.119000	8.327000	-2.257000
C	8.127000	0.099000	-0.293000	C	2.078000	6.993000	2.446000
H	9.155000	-0.145000	-0.023000	H	2.488000	8.003000	2.484000
H	8.137000	0.653000	-1.234000	H	1.545000	6.799000	3.380000
H	7.686000	0.732000	0.481000	H	2.894000	6.271000	2.352000
C	8.012000	-2.130000	-1.467000	C	-0.009000	7.930000	1.386000
H	8.116000	-1.606000	-2.421000	H	-0.463000	7.838000	2.375000
H	9.007000	-2.371000	-1.086000	H	0.437000	8.923000	1.296000
H	7.459000	-3.054000	-1.643000	H	-0.792000	7.811000	0.637000
H	5.843000	-3.883000	-0.534000	H	-0.638000	6.868000	-1.231000

H	4.801000	-2.412000	-1.990000	H	-1.425000	5.310000	0.259000
H	5.967000	-0.383000	-1.960000	H	-0.082000	5.305000	2.169000
N	7.221000	-1.897000	0.813000	N	1.815000	7.104000	0.010000
O	8.170000	-1.863000	1.541000	O	2.693000	7.866000	0.010000
C	2.972000	-0.680000	-1.532000	C	-0.709000	2.765000	0.667000
H	3.469000	0.299000	-1.555000	H	-0.260000	3.017000	1.635000
H	3.068000	-1.122000	-2.536000	H	-1.801000	2.859000	0.790000
C	1.516000	-0.509000	-1.177000	C	-0.353000	1.340000	0.314000
C	0.871000	0.700000	-1.431000	C	-1.333000	0.357000	0.224000
C	0.791000	-1.573000	-0.640000	C	0.985000	0.997000	0.087000
C	-0.489000	0.845000	-1.156000	C	-0.993000	-0.967000	-0.080000
H	1.427000	1.541000	-1.838000	H	-2.377000	0.610000	0.386000
C	-0.566000	-1.435000	-0.353000	C	1.340000	-0.309000	-0.229000
H	1.300000	-2.513000	-0.439000	H	1.747000	1.771000	0.149000
C	-1.202000	-0.222000	-0.613000	C	0.342000	-1.287000	-0.307000
H	-2.257000	-0.111000	-0.382000	H	0.623000	-2.310000	-0.550000
C	-1.173000	2.155000	-1.450000	C	-2.052000	-2.045000	-0.125000
C	-1.306000	-2.603000	0.254000	C	2.778000	-0.681000	-0.505000
O	-0.442000	3.199000	-0.822000	H	-1.665000	-2.919000	-0.675000
O	-2.686000	-2.310000	0.311000	H	-2.254000	-2.386000	0.898000
H	-1.201000	2.343000	-2.535000	H	2.876000	-0.969000	-1.559000
H	-2.210000	2.127000	-1.089000	H	3.426000	0.197000	-0.360000
H	-1.116000	-3.504000	-0.348000	C	4.557000	-2.266000	0.035000
H	-0.927000	-2.806000	1.267000	C	5.060000	-3.041000	1.268000
O	3.584000	-1.540000	-0.585000	C	4.587000	-3.119000	-1.248000
C	-3.476000	-3.306000	0.937000	H	5.225000	-1.407000	-0.132000
C	-4.754000	-2.575000	1.384000	C	6.039000	-4.181000	0.985000
C	-3.761000	-4.482000	-0.008000	H	4.203000	-3.446000	1.818000
H	-2.937000	-3.695000	1.810000	H	5.576000	-2.361000	1.951000
C	-6.066000	-3.351000	1.264000	C	4.390000	-4.621000	-1.049000
H	-4.832000	-1.649000	0.805000	H	3.786000	-2.801000	-1.921000
H	-4.672000	-2.276000	2.432000	H	5.519000	-2.959000	-1.798000
C	-4.936000	-4.269000	-0.961000	C	6.350000	-4.993000	2.237000
H	-2.884000	-4.668000	-0.635000	C	7.345000	-3.703000	0.318000
H	-3.921000	-5.403000	0.559000	N	5.391000	-5.111000	-0.020000
C	-7.273000	-2.438000	1.458000	C	3.015000	-4.984000	-0.456000
C	-6.148000	-4.558000	2.220000	C	4.645000	-5.398000	-2.338000
N	-6.163000	-3.942000	-0.130000	H	7.085000	-5.774000	2.037000
C	-4.757000	-3.044000	-1.880000	H	6.766000	-4.305000	2.976000
C	-5.247000	-5.521000	-1.775000	H	5.444000	-5.444000	2.651000
H	-8.208000	-3.000000	1.447000	H	7.871000	-3.108000	1.069000
H	-7.166000	-1.959000	2.434000	H	7.971000	-4.556000	0.045000
H	-7.305000	-1.661000	0.690000	H	7.180000	-3.078000	-0.559000
H	-6.164000	-4.145000	3.233000	O	5.663000	-6.262000	0.006000
H	-7.073000	-5.114000	2.053000	H	2.268000	-4.652000	-1.182000
H	-5.297000	-5.234000	2.142000	H	2.934000	-6.066000	-0.330000

O	-7.246000	-4.148000	-0.594000	H	2.810000	-4.477000	0.487000
H	-3.878000	-3.246000	-2.497000	H	4.495000	-6.469000	-2.198000
H	-5.631000	-2.929000	-2.525000	H	3.928000	-5.040000	-3.081000
H	-4.575000	-2.122000	-1.328000	H	5.656000	-5.215000	-2.711000
H	-6.076000	-5.356000	-2.464000	C	-4.375000	-2.539000	-0.699000
H	-4.354000	-5.761000	-2.357000	C	-4.779000	-2.829000	0.762000
H	-5.476000	-6.368000	-1.122000	C	-5.557000	-2.018000	-1.539000
C	-0.881000	4.493000	-1.192000	H	-4.043000	-3.484000	-1.160000
C	-2.292000	4.757000	-0.620000	C	-6.234000	-3.234000	0.978000
C	0.166000	5.486000	-0.682000	H	-4.556000	-1.952000	1.377000
H	-0.909000	4.558000	-2.289000	H	-4.181000	-3.651000	1.167000
C	-2.564000	6.184000	-0.140000	C	-6.644000	-1.271000	-0.761000
H	-2.463000	4.068000	0.214000	H	-5.193000	-1.318000	-2.299000
H	-3.061000	4.540000	-1.367000	H	-6.027000	-2.840000	-2.087000
C	-0.059000	6.003000	0.737000	C	-6.600000	-3.252000	2.462000
H	1.136000	4.980000	-0.687000	C	-6.587000	-4.576000	0.309000
H	0.257000	6.336000	-1.363000	N	-7.144000	-2.223000	0.302000
C	-3.896000	6.288000	0.594000	C	-6.117000	-0.032000	-0.009000
C	-2.490000	7.233000	-1.267000	C	-7.829000	-0.899000	-1.644000
N	-1.469000	6.562000	0.837000	H	-7.617000	-3.617000	2.616000
C	-0.013000	4.883000	1.795000	H	-5.905000	-3.931000	2.961000
C	0.904000	7.134000	1.090000	H	-6.495000	-2.259000	2.907000
H	-4.118000	7.317000	0.879000	H	-5.940000	-5.333000	0.759000
H	-4.673000	5.941000	-0.090000	H	-7.629000	-4.836000	0.504000
H	-3.905000	5.653000	1.484000	H	-6.410000	-4.570000	-0.768000
H	-3.331000	7.024000	-1.934000	O	-8.289000	-2.212000	0.600000
H	-2.608000	8.239000	-0.860000	H	-5.968000	0.739000	-0.770000
H	-1.571000	7.182000	-1.850000	H	-6.859000	0.329000	0.707000
O	-1.722000	7.310000	1.732000	H	-5.160000	-0.207000	0.479000
H	1.012000	4.504000	1.799000	H	-8.576000	-0.324000	-1.093000
H	-0.247000	5.285000	2.784000	H	-7.447000	-0.282000	-2.459000
H	-0.679000	4.052000	1.558000	H	-8.299000	-1.790000	-2.070000
H	0.770000	7.465000	2.121000	N	3.191000	-1.820000	0.318000
H	1.918000	6.745000	0.976000	H	3.139000	-1.525000	1.293000
H	0.777000	7.982000	0.412000	N	-0.178000	3.691000	-0.336000
				H	-0.669000	3.510000	-1.212000
				N	-3.302000	-1.543000	-0.696000
				H	-3.113000	-1.276000	-1.661000

Hub^3-TEMPO (-1951.87727814) **Hub^4-TEMPO** (-1892.31045688)

C	-3.600000	4.048000	0.526000	C	-3.566000	4.183000	0.404000
C	-2.599000	2.909000	0.325000	C	-2.486000	3.106000	0.360000
C	-1.621000	3.087000	-0.850000	C	-1.424000	3.287000	-0.747000
C	-1.402000	4.545000	-1.249000	C	-1.174000	4.766000	-1.091000
C	-1.503000	5.555000	-0.107000	C	-1.473000	5.773000	0.022000
H	-2.027000	2.772000	1.248000	H	-1.995000	3.045000	1.337000

H	-0.400000	4.643000	-1.677000	H	-0.125000	4.916000	-1.369000
C	-0.471000	5.312000	1.013000	C	-0.561000	5.597000	1.251000
H	0.515000	5.443000	0.560000	H	0.449000	5.869000	0.931000
H	-0.600000	6.050000	1.807000	H	-0.866000	6.272000	2.054000
H	-0.520000	4.307000	1.432000	H	-0.541000	4.571000	1.623000
C	-1.429000	6.992000	-0.612000	C	-1.424000	7.210000	-0.492000
H	-1.474000	7.707000	0.211000	H	-1.571000	7.928000	0.315000
H	-0.472000	7.110000	-1.124000	H	-0.435000	7.370000	-0.926000
H	-2.234000	7.200000	-1.322000	H	-2.176000	7.374000	-1.269000
C	-4.346000	3.905000	1.849000	C	-4.494000	4.010000	1.601000
H	-5.110000	4.674000	1.964000	H	-5.285000	4.761000	1.610000
H	-4.834000	2.927000	1.846000	H	-4.952000	3.023000	1.518000
H	-3.654000	3.946000	2.694000	H	-3.935000	4.058000	2.540000
C	-4.596000	4.192000	-0.643000	C	-4.375000	4.281000	-0.905000
H	-5.203000	3.283000	-0.645000	H	-4.922000	3.339000	-0.999000
H	-5.250000	5.052000	-0.479000	H	-5.094000	5.102000	-0.851000
H	-4.113000	4.285000	-1.616000	H	-3.752000	4.404000	-1.792000
H	-2.101000	4.824000	-2.041000	H	-1.750000	5.044000	-1.977000
H	-1.961000	2.521000	-1.719000	H	-1.789000	2.789000	-1.651000
H	-3.184000	1.997000	0.187000	H	-2.994000	2.150000	0.214000
N	-2.850000	5.367000	0.563000	N	-2.878000	5.525000	0.543000
O	-3.335000	6.279000	1.138000	O	-3.444000	6.411000	1.085000
C	-0.156000	1.266000	-0.412000	C	-0.046000	1.291000	-0.329000
C	-0.868000	-0.832000	-0.584000	C	-0.920000	-0.770000	-0.507000
C	1.243000	-0.417000	-0.022000	C	1.253000	-0.512000	-0.013000
O	-1.882000	-1.646000	-0.858000	C	3.621000	-0.291000	0.631000
O	2.430000	-0.921000	0.303000	C	4.641000	-1.253000	1.264000
O	-0.325000	2.583000	-0.471000	C	4.209000	0.515000	-0.551000
C	3.500000	-0.007000	0.604000	H	3.301000	0.439000	1.381000
C	4.493000	-0.844000	1.422000	C	6.091000	-0.777000	1.202000
C	4.097000	0.580000	-0.680000	H	4.584000	-2.241000	0.795000
H	3.092000	0.809000	1.202000	H	4.404000	-1.403000	2.321000
C	5.961000	-0.455000	1.251000	C	5.386000	-0.131000	-1.279000
H	4.368000	-1.900000	1.165000	H	3.426000	0.675000	-1.295000
H	4.268000	-0.756000	2.488000	H	4.509000	1.511000	-0.211000
C	5.240000	-0.225000	-1.299000	C	7.058000	-1.838000	1.720000
H	3.316000	0.656000	-1.442000	C	6.320000	0.567000	1.920000
H	4.432000	1.604000	-0.498000	N	6.436000	-0.513000	-0.252000
C	6.888000	-1.450000	1.940000	C	5.011000	-1.450000	-1.982000
C	6.265000	0.983000	1.716000	C	6.042000	0.831000	-2.265000
N	6.290000	-0.465000	-0.230000	H	8.087000	-1.475000	1.717000
C	4.805000	-1.635000	-1.747000	H	6.774000	-2.067000	2.749000
C	5.910000	0.530000	-2.442000	H	6.992000	-2.753000	1.127000
H	7.931000	-1.141000	1.866000	H	6.123000	0.392000	2.980000
H	6.609000	-1.481000	2.996000	H	7.357000	0.888000	1.802000
H	6.772000	-2.452000	1.519000	H	5.655000	1.359000	1.573000

H	6.096000	1.000000	2.796000	O	7.564000	-0.604000	-0.594000
H	7.309000	1.234000	1.518000	H	4.317000	-1.184000	-2.784000
H	5.619000	1.734000	1.257000	H	5.896000	-1.915000	-2.422000
O	7.407000	-0.664000	-0.562000	H	4.511000	-2.162000	-1.325000
H	4.094000	-1.492000	-2.565000	H	6.838000	0.346000	-2.831000
H	5.664000	-2.198000	-2.118000	H	5.269000	1.160000	-2.963000
H	4.305000	-2.200000	-0.959000	H	6.443000	1.709000	-1.751000
H	6.692000	-0.067000	-2.912000	C	-1.920000	-3.005000	-0.811000
H	5.140000	0.744000	-3.187000	C	-1.757000	-3.600000	0.606000
H	6.331000	1.476000	-2.093000	C	-3.149000	-3.565000	-1.549000
C	-1.643000	-3.066000	-0.827000	H	-1.031000	-3.262000	-1.395000
C	-1.547000	-3.533000	0.637000	C	-2.299000	-5.016000	0.784000
C	-2.797000	-3.702000	-1.597000	H	-2.231000	-2.939000	1.338000
H	-0.706000	-3.261000	-1.352000	H	-0.696000	-3.629000	0.863000
C	-2.187000	-4.888000	0.943000	C	-4.303000	-4.033000	-0.660000
H	-1.992000	-2.771000	1.284000	H	-3.561000	-2.808000	-2.223000
H	-0.498000	-3.610000	0.934000	H	-2.851000	-4.394000	-2.195000
C	-4.036000	-4.027000	-0.761000	C	-2.250000	-5.466000	2.242000
H	-3.112000	-3.015000	-2.388000	C	-1.604000	-6.052000	-0.122000
H	-2.455000	-4.612000	-2.098000	N	-3.752000	-5.026000	0.348000
C	-2.300000	-5.122000	2.448000	C	-4.913000	-2.900000	0.189000
C	-1.454000	-6.061000	0.263000	C	-5.385000	-4.745000	-1.465000
N	-3.594000	-4.929000	0.371000	H	-2.595000	-6.494000	2.357000
C	-4.650000	-2.796000	-0.066000	H	-1.207000	-5.411000	2.563000
C	-5.088000	-4.771000	-1.575000	H	-2.845000	-4.806000	2.878000
H	-2.678000	-6.121000	2.669000	H	-0.564000	-6.103000	0.211000
H	-1.298000	-5.025000	2.872000	H	-2.062000	-7.036000	0.004000
H	-2.949000	-4.374000	2.913000	H	-1.613000	-5.782000	-1.178000
H	-0.460000	-6.109000	0.715000	O	-4.484000	-5.831000	0.809000
H	-1.976000	-7.001000	0.458000	H	-5.409000	-2.221000	-0.511000
H	-1.336000	-5.928000	-0.813000	H	-5.664000	-3.297000	0.875000
O	-4.365000	-5.702000	0.824000	H	-4.169000	-2.334000	0.750000
H	-4.978000	-2.122000	-0.862000	H	-6.229000	-5.032000	-0.837000
H	-5.517000	-3.095000	0.528000	H	-5.741000	-4.046000	-2.226000
H	-3.943000	-2.256000	0.564000	H	-4.984000	-5.630000	-1.966000
H	-5.977000	-4.987000	-0.981000	N	0.237000	-1.371000	-0.222000
H	-5.374000	-4.125000	-2.407000	N	-1.132000	0.557000	-0.581000
H	-4.684000	-5.703000	-1.979000	N	1.184000	0.821000	-0.043000
N	0.308000	-1.344000	-0.259000	N	2.462000	-1.073000	0.238000
N	-1.176000	0.466000	-0.675000	H	2.454000	-2.074000	0.382000
N	1.086000	0.895000	-0.083000	N	-1.997000	-1.555000	-0.743000
				H	-2.830000	-1.073000	-1.053000
				N	-0.181000	2.637000	-0.360000
				H	0.670000	3.173000	-0.262000

**Hub⁵-TEMPO** (-2243.67520548)				**Hub⁶-TEMPO** (-2184.08096489)			
C	5.396000	-4.459000	0.623000	C	5.983000	3.802000	0.968000
C	4.569000	-3.219000	0.281000	C	4.546000	3.394000	0.669000
C	3.662000	-3.348000	-0.957000	C	4.273000	2.933000	-0.789000
C	3.279000	-4.788000	-1.292000	C	5.546000	2.483000	-1.513000
C	3.171000	-5.729000	-0.093000	C	6.567000	1.755000	-0.629000
H	3.953000	-2.953000	1.146000	H	4.231000	2.618000	1.372000
H	2.302000	-4.783000	-1.784000	H	5.307000	1.798000	-2.333000
C	2.090000	-5.300000	0.918000	C	5.994000	0.510000	0.079000
H	1.131000	-5.381000	0.399000	H	5.727000	-0.196000	-0.713000
H	2.087000	-5.975000	1.777000	H	6.755000	0.047000	0.710000
H	2.205000	-4.270000	1.259000	H	5.096000	0.713000	0.661000
C	2.967000	-7.177000	-0.527000	C	7.833000	1.408000	-1.399000
H	2.844000	-7.839000	0.331000	H	8.499000	0.768000	-0.818000
H	2.055000	-7.212000	-1.127000	H	7.538000	0.879000	-2.307000
H	3.803000	-7.525000	-1.141000	H	8.370000	2.316000	-1.689000
C	6.069000	-4.325000	1.986000	C	6.225000	3.975000	2.467000
H	6.708000	-5.181000	2.203000	H	7.232000	4.341000	2.671000
H	6.689000	-3.426000	1.957000	H	5.507000	4.718000	2.824000
H	5.326000	-4.212000	2.780000	H	6.050000	3.039000	3.003000
C	6.436000	-4.810000	-0.459000	C	6.401000	5.064000	0.185000
H	7.154000	-3.986000	-0.472000	H	5.814000	5.893000	0.587000
H	6.962000	-5.731000	-0.198000	H	7.462000	5.278000	0.330000
H	6.010000	-4.907000	-1.458000	H	6.191000	4.980000	-0.884000
H	3.983000	-5.203000	-2.016000	H	6.044000	3.334000	-1.987000
H	4.138000	-2.887000	-1.825000	H	3.840000	3.778000	-1.331000
H	5.277000	-2.399000	0.142000	H	3.918000	4.261000	0.887000
N	4.472000	-5.661000	0.686000	N	6.942000	2.716000	0.477000
O	4.775000	-6.579000	1.366000	O	7.989000	2.603000	1.013000
C	1.173000	-0.664000	-0.518000	C	1.072000	0.966000	-0.239000
C	1.103000	0.727000	-0.581000	C	-0.284000	1.281000	-0.159000
C	0.032000	-1.414000	-0.244000	C	1.481000	-0.358000	-0.120000
C	-0.116000	1.367000	-0.367000	C	-1.228000	0.272000	0.013000
H	2.002000	1.296000	-0.796000	H	-0.585000	2.322000	-0.215000
C	-1.184000	-0.767000	-0.027000	C	0.542000	-1.374000	0.074000
H	0.076000	-2.496000	-0.194000	H	2.531000	-0.633000	-0.120000
C	-1.262000	0.623000	-0.088000	C	-0.814000	-1.056000	0.123000
H	-2.202000	1.137000	0.079000	H	-1.582000	-1.810000	0.259000
O	0.915000	3.455000	-0.711000	C	0.541000	-5.097000	0.898000
O	-3.482000	-0.881000	0.475000	C	-0.692000	-5.774000	1.551000
O	2.431000	-2.644000	-0.710000	C	0.905000	-5.766000	-0.441000
C	-4.682000	-1.610000	0.792000	H	1.414000	-5.168000	1.555000
C	-5.592000	-0.583000	1.478000	C	-1.036000	-7.186000	1.053000
C	-5.289000	-2.225000	-0.473000	H	-1.582000	-5.159000	1.364000
H	-4.412000	-2.418000	1.473000	H	-0.567000	-5.837000	2.635000
C	-7.088000	-0.755000	1.215000	C	-0.306000	-6.153000	-1.274000

H	-5.288000	0.423000	1.175000	H	1.515000	-5.075000	-1.028000
H	-5.461000	-0.630000	2.562000	H	1.534000	-6.641000	-0.266000
C	-6.239000	-1.310000	-1.245000	C	-2.338000	-7.699000	1.650000
H	-4.486000	-2.510000	-1.159000	C	0.072000	-8.243000	1.249000
H	-5.809000	-3.153000	-0.222000	N	-1.252000	-7.009000	-0.438000
C	-7.885000	0.442000	1.725000	C	-1.169000	-4.933000	-1.639000
C	-7.655000	-2.069000	1.788000	C	0.063000	-6.972000	-2.516000
N	-7.318000	-0.848000	-0.284000	H	-2.518000	-8.741000	1.383000
C	-5.569000	-0.012000	-1.740000	H	-2.263000	-7.627000	2.736000
C	-6.921000	-2.041000	-2.397000	H	-3.186000	-7.097000	1.314000
H	-8.958000	0.290000	1.601000	H	0.014000	-8.529000	2.303000
H	-7.669000	0.551000	2.790000	H	-0.140000	-9.131000	0.649000
H	-7.583000	1.359000	1.212000	H	1.082000	-7.895000	1.046000
H	-7.581000	-1.989000	2.875000	O	-2.214000	-7.448000	-0.963000
H	-8.706000	-2.183000	1.515000	H	-0.587000	-4.325000	-2.337000
H	-7.100000	-2.954000	1.472000	H	-2.095000	-5.249000	-2.124000
O	-8.377000	-0.549000	-0.714000	H	-1.386000	-4.322000	-0.763000
H	-4.799000	-0.317000	-2.454000	H	-0.801000	-7.112000	-3.167000
H	-6.300000	0.617000	-2.253000	H	0.825000	-6.411000	-3.061000
H	-5.088000	0.557000	-0.945000	H	0.482000	-7.944000	-2.239000
H	-7.578000	-1.377000	-2.959000	C	-4.534000	2.045000	-0.396000
H	-6.135000	-2.398000	-3.066000	C	-5.026000	2.497000	0.992000
H	-7.490000	-2.901000	-2.035000	C	-4.838000	3.072000	-1.502000
C	0.878000	4.891000	-0.804000	H	-5.033000	1.102000	-0.640000
C	0.728000	5.482000	0.610000	C	-6.337000	3.287000	0.980000
C	2.164000	5.309000	-1.512000	H	-4.241000	3.082000	1.479000
H	0.018000	5.162000	-1.420000	H	-5.174000	1.607000	1.612000
C	1.515000	6.766000	0.876000	C	-5.099000	4.503000	-1.032000
H	1.023000	4.726000	1.345000	H	-4.001000	3.147000	-2.203000
H	-0.321000	5.711000	0.811000	H	-5.687000	2.739000	-2.105000
C	3.363000	5.541000	-0.593000	C	-6.660000	3.879000	2.349000
H	2.448000	4.522000	-2.217000	C	-7.529000	2.472000	0.448000
H	1.987000	6.207000	-2.110000	N	-6.188000	4.442000	0.011000
C	1.514000	7.124000	2.360000	C	-3.878000	5.147000	-0.343000
C	1.025000	7.959000	0.030000	C	-5.582000	5.380000	-2.181000
N	2.959000	6.558000	0.457000	H	-7.632000	4.373000	2.349000
C	3.762000	4.287000	0.212000	H	-6.691000	3.051000	3.059000
C	4.561000	6.104000	-1.350000	H	-5.886000	4.587000	2.659000
H	2.009000	8.078000	2.541000	H	-7.707000	1.679000	1.179000
H	0.472000	7.205000	2.676000	H	-8.422000	3.098000	0.386000
H	1.997000	6.342000	2.952000	H	-7.334000	2.015000	-0.524000
H	0.007000	8.176000	0.362000	O	-6.976000	5.325000	0.064000
H	1.650000	8.836000	0.211000	H	-3.138000	5.300000	-1.135000
H	0.998000	7.748000	-1.040000	H	-4.137000	6.117000	0.088000
O	3.799000	7.215000	0.967000	H	-3.407000	4.538000	0.430000
H	4.077000	3.537000	-0.519000	H	-5.715000	6.420000	-1.887000

H	4.602000	4.516000	0.872000	H	-4.823000	5.340000	-2.965000
H	2.941000	3.868000	0.794000	H	-6.518000	4.992000	-2.584000
H	5.425000	6.227000	-0.695000	N	0.198000	-3.687000	0.735000
H	4.818000	5.388000	-2.134000	H	-0.640000	-3.358000	1.194000
H	4.317000	7.061000	-1.816000	N	-3.102000	1.778000	-0.326000
C	-2.376000	-1.613000	0.267000	H	-2.503000	2.238000	-1.000000
O	-2.356000	-2.821000	0.313000	N	3.281000	1.860000	-0.778000
C	-0.248000	2.851000	-0.421000	H	3.480000	0.991000	-1.257000
O	-1.280000	3.450000	-0.227000	C	-2.692000	0.568000	0.154000
C	2.499000	-1.304000	-0.752000	O	-3.468000	-0.227000	0.667000
O	3.523000	-0.695000	-0.960000	C	1.072000	-2.772000	0.246000
				O	2.207000	-3.072000	-0.107000
				C	2.028000	2.121000	-0.330000
				O	1.681000	3.252000	-0.008000

Hub[7]-TEMPO (-2401.04333070) **Hub[8]-TEMPO** (-2877.01747235)

C	-6.358000	-5.824000	-1.240000	C	-10.737000	-4.218000	-0.581000
C	-5.069000	-5.008000	-1.366000	C	-9.508000	-3.358000	-0.883000
C	-4.967000	-3.953000	-0.269000	C	-9.057000	-2.408000	0.249000
C	-5.401000	-4.457000	1.128000	C	-9.472000	-2.904000	1.643000
C	-5.548000	-5.972000	1.275000	C	-9.574000	-4.421000	1.800000
H	-4.200000	-5.667000	-1.362000	H	-8.675000	-4.012000	-1.152000
H	-4.663000	-4.151000	1.874000	H	-8.757000	-2.561000	2.400000
C	-4.200000	-6.724000	1.174000	C	-8.223000	-5.132000	1.583000
H	-3.594000	-6.376000	2.015000	H	-7.568000	-4.788000	2.388000
H	-4.367000	-7.796000	1.267000	H	-8.347000	-6.214000	1.663000
H	-3.665000	-6.508000	0.252000	H	-7.758000	-4.878000	0.629000
C	-6.274000	-6.330000	2.569000	C	-10.178000	-4.809000	3.147000
H	-6.295000	-7.410000	2.718000	H	-10.177000	-5.891000	3.286000
H	-5.725000	-5.867000	3.390000	H	-9.558000	-4.355000	3.922000
H	-7.290000	-5.933000	2.554000	H	-11.197000	-4.429000	3.247000
C	-6.467000	-6.893000	-2.317000	C	-10.979000	-5.257000	-1.673000
H	-7.396000	-7.454000	-2.209000	H	-11.885000	-5.833000	-1.482000
H	-6.484000	-6.370000	-3.274000	H	-11.103000	-4.717000	-2.614000
H	-5.615000	-7.573000	-2.287000	H	-10.128000	-5.939000	-1.771000
C	-7.613000	-4.930000	-1.241000	C	-12.016000	-3.391000	-0.338000
H	-7.591000	-4.389000	-2.190000	H	-12.225000	-2.872000	-1.277000
H	-8.503000	-5.559000	-1.186000	H	-12.855000	-4.051000	-0.103000
H	-7.635000	-4.207000	-0.429000	H	-11.916000	-2.641000	0.447000
H	-6.339000	-3.977000	1.418000	H	-10.426000	-2.456000	1.927000
H	-5.591000	-3.103000	-0.543000	H	-9.520000	-1.428000	0.089000
H	-5.096000	-4.513000	-2.339000	H	-9.746000	-2.778000	-1.776000
N	-6.359000	-6.497000	0.114000	N	-10.489000	-4.959000	0.714000
O	-7.049000	-7.424000	0.270000	O	-10.998000	-6.026000	0.875000
C	-0.886000	-1.088000	-0.070000	C	-1.408000	-0.353000	-0.686000
C	-1.335000	0.231000	-0.101000	C	-0.990000	0.981000	-0.690000

C	0.486000	-1.348000	-0.005000	C	-0.444000	-1.365000	-0.670000
C	-0.424000	1.290000	-0.080000	C	0.370000	1.308000	-0.692000
H	-2.395000	0.447000	-0.173000	H	-1.732000	1.774000	-0.698000
C	1.405000	-0.299000	0.023000	C	0.921000	-1.060000	-0.678000
H	0.833000	-2.375000	0.016000	H	-0.763000	-2.403000	-0.660000
C	0.943000	1.022000	-0.024000	C	1.313000	0.279000	-0.692000
H	1.655000	1.838000	-0.007000	H	2.372000	0.525000	-0.693000
C	5.955000	-2.348000	-0.192000	C	7.174000	-5.983000	-0.644000
C	6.844000	-1.720000	-1.260000	C	8.535000	-5.273000	-0.760000
C	6.697000	-2.661000	1.121000	C	7.135000	-7.029000	0.495000
H	5.524000	-3.272000	-0.574000	H	6.958000	-6.514000	-1.578000
C	8.262000	-2.271000	-1.302000	C	9.736000	-6.154000	-0.419000
H	6.881000	-0.635000	-1.122000	H	8.567000	-4.386000	-0.114000
H	6.401000	-1.906000	-2.240000	H	8.687000	-4.915000	-1.782000
C	8.053000	-1.975000	1.330000	C	8.163000	-6.826000	1.611000
H	6.081000	-2.370000	1.974000	H	6.153000	-7.006000	0.970000
H	6.832000	-3.739000	1.200000	H	7.242000	-8.036000	0.084000
C	9.114000	-1.529000	-2.318000	C	11.043000	-5.372000	-0.472000
C	8.340000	-3.793000	-1.530000	C	9.827000	-7.434000	-1.271000
N	8.883000	-2.067000	0.062000	N	9.539000	-6.641000	1.005000
C	7.935000	-0.466000	1.602000	C	7.896000	-5.540000	2.417000
C	8.843000	-2.672000	2.438000	C	8.246000	-8.030000	2.545000
H	10.098000	-1.986000	-2.414000	H	11.895000	-5.994000	-0.196000
H	8.601000	-1.591000	-3.279000	H	11.178000	-5.032000	-1.501000
H	9.212000	-0.482000	-2.031000	H	11.000000	-4.498000	0.184000
H	7.862000	-3.972000	-2.496000	H	9.985000	-7.115000	-2.304000
H	9.383000	-4.112000	-1.578000	H	10.681000	-8.035000	-0.952000
H	7.823000	-4.381000	-0.772000	H	8.926000	-8.047000	-1.234000
O	10.048000	-1.900000	0.139000	O	10.487000	-6.834000	1.684000
H	7.410000	-0.374000	2.558000	H	6.990000	-5.733000	2.999000
H	8.925000	-0.013000	1.691000	H	8.719000	-5.338000	3.106000
H	7.358000	0.047000	0.834000	H	7.708000	-4.672000	1.787000
H	9.774000	-2.146000	2.651000	H	8.916000	-7.834000	3.384000
H	8.217000	-2.664000	3.332000	H	7.241000	-8.211000	2.934000
H	9.057000	-3.708000	2.167000	H	8.581000	-8.923000	2.009000
C	-0.947000	6.254000	0.151000	C	2.388000	8.954000	0.372000
C	-1.289000	6.948000	-1.174000	C	1.788000	9.793000	-0.781000
C	-1.703000	6.825000	1.362000	C	2.128000	9.573000	1.753000
H	0.118000	6.350000	0.360000	H	3.472000	8.883000	0.231000
C	-1.525000	8.445000	-1.009000	C	1.666000	11.295000	-0.515000
H	-2.161000	6.488000	-1.647000	H	0.799000	9.398000	-1.035000
H	-0.452000	6.839000	-1.868000	H	2.400000	9.683000	-1.677000
C	-2.956000	7.661000	1.079000	C	0.873000	10.436000	1.873000
H	-2.024000	6.007000	2.012000	H	2.026000	8.785000	2.507000
H	-1.012000	7.423000	1.955000	H	2.994000	10.160000	2.067000
C	-1.858000	9.120000	-2.333000	C	0.906000	12.004000	-1.629000

C	-0.354000	9.156000	-0.314000	C	3.026000	11.980000	-0.272000
N	-2.706000	8.618000	-0.070000	N	0.886000	11.481000	0.770000
C	-4.162000	6.825000	0.616000	C	-0.420000	9.637000	1.627000
C	-3.300000	8.502000	2.313000	C	0.818000	11.175000	3.208000
H	-2.005000	10.192000	-2.203000	H	0.861000	13.080000	-1.464000
H	-0.996000	8.961000	-2.982000	H	1.446000	11.817000	-2.560000
H	-2.738000	8.674000	-2.801000	H	-0.106000	11.605000	-1.728000
H	0.530000	8.931000	-0.914000	H	3.577000	11.907000	-1.213000
H	-0.522000	10.234000	-0.300000	H	2.881000	13.036000	-0.030000
H	-0.189000	8.803000	0.700000	H	3.620000	11.508000	0.508000
O	-3.364000	9.596000	-0.149000	O	0.219000	12.446000	0.904000
H	-4.331000	6.085000	1.402000	H	-0.463000	8.871000	2.404000
H	-5.041000	7.464000	0.519000	H	-1.291000	10.290000	1.715000
H	-3.975000	6.296000	-0.319000	H	-0.424000	9.137000	0.657000
H	-4.203000	9.087000	2.145000	H	-0.102000	11.752000	3.301000
H	-3.473000	7.807000	3.137000	H	0.842000	10.424000	4.001000
H	-2.468000	9.167000	2.577000	H	1.680000	11.836000	3.327000
C	2.845000	-0.588000	0.072000	N	6.151000	-4.957000	-0.447000
C	3.513000	-1.779000	-0.107000	H	6.408000	-4.013000	-0.717000
N	4.975000	-0.147000	0.202000	N	1.822000	7.603000	0.319000
H	3.197000	-2.789000	-0.315000	H	1.693000	7.125000	1.204000
N	4.814000	-1.455000	-0.024000	N	-7.604000	-2.252000	0.186000
N	3.791000	0.374000	0.272000	H	-7.121000	-2.132000	1.069000
C	-1.857000	-2.186000	-0.099000	C	2.134000	6.814000	-0.755000
C	-3.227000	-2.146000	-0.229000	O	2.754000	7.244000	-1.722000
N	-3.605000	-3.434000	-0.229000	C	4.844000	-5.308000	-0.666000
H	-3.939000	-1.345000	-0.359000	O	4.509000	-6.472000	-0.860000
N	-2.556000	-4.247000	-0.075000	C	-7.061000	-1.590000	-0.882000
N	-1.499000	-3.502000	0.011000	O	-7.735000	-1.264000	-1.853000
C	-0.906000	2.676000	-0.093000	C	1.936000	-2.143000	-0.664000
C	-0.212000	3.857000	0.051000	C	3.149000	-1.978000	0.016000
N	-1.152000	4.813000	0.012000	C	1.701000	-3.354000	-1.333000
H	0.833000	4.094000	0.186000	C	4.105000	-2.986000	0.020000
N	-2.370000	4.285000	-0.139000	H	3.338000	-1.062000	0.568000
N	-2.228000	3.000000	-0.214000	C	2.648000	-4.367000	-1.316000
				H	0.780000	-3.494000	-1.890000
				C	3.860000	-4.185000	-0.649000
				H	5.026000	-2.846000	0.580000
				H	2.467000	-5.303000	-1.834000
				C	-2.857000	-0.681000	-0.718000
				C	-3.366000	-1.764000	0.009000
				C	-3.740000	0.086000	-1.489000
				C	-4.722000	-2.067000	-0.023000
				H	-2.700000	-2.365000	0.622000
				C	-5.094000	-0.223000	-1.534000
				H	-3.360000	0.912000	-2.082000

	C -5.596000 -1.294000 -0.791000
	H -5.092000 -2.922000 0.537000
	H -5.774000 0.358000 -2.148000
	C 0.814000 2.726000 -0.690000
	C 1.898000 3.132000 -1.478000
	C 0.154000 3.685000 0.090000
	C 2.308000 4.457000 -1.486000
	H 2.404000 2.411000 -2.113000
	C 0.572000 5.010000 0.094000
	H -0.683000 3.389000 0.716000
	C 1.655000 5.401000 -0.692000
	H 3.135000 4.777000 -2.111000
	H 0.035000 5.739000 0.694000

XYZ coordinates and total energies of R to P for the conversion HMF to DFF.

TEMPO

R(-1267.763141)	TS(-1267.73946)
C 2.9610 -0.1050 1.0850	C 2.5310 -2.1770 0.0490
C 4.2920 -0.1550 1.5000	C 3.2550 -0.9650 0.6810
C 5.1480 -1.0710 0.9020	C 2.5820 0.5460 -1.3600
C 4.6560 -1.9090 -0.0910	C 2.5610 -0.8090 -2.0860
C 3.3160 -1.8070 -0.4580	C 1.8250 -1.8750 -1.2800
N 2.4930 -0.9190 0.1260	H 3.2780 -2.9640 -0.0960
H 6.1890 -1.1270 1.2030	H 1.7970 -2.5630 0.7630
H 4.6420 0.5180 2.2750	H 2.0830 -0.6560 -3.0600
H 5.2980 -2.6320 -0.5840	H 3.5830 -1.1420 -2.2970
C -1.0370 -1.2080 1.5630	H 0.8100 -1.5220 -1.0730
C -1.7720 -2.1780 0.6020	H 1.7350 -2.7940 -1.8670
C -3.3600 -0.1730 -0.1590	N 2.6690 0.2970 0.1220
C -3.0350 0.3290 1.2510	O 2.7720 1.3400 0.8250
C -1.5380 0.2400 1.5230	C 1.3070 1.3450 -1.6320
H -1.1560 -1.6300 2.5660	H 1.2790 2.2500 -1.0170
H 0.0310 -1.2390 1.3230	H 1.2990 1.6480 -2.6830
H -3.3510 1.3750 1.2980	H 0.4210 0.7510 -1.4160
H -3.6140 -0.2170 2.0040	C 3.7910 1.3910 -1.7850
H -1.0180 0.7840 0.7310	H 3.7330 1.5620 -2.8640
H -1.2990 0.7420 2.4640	H 3.7790 2.3590 -1.2790
N -2.4680 -1.3770 -0.4740	H 4.7380 0.8930 -1.5710
O -2.3970 -1.7380 -1.5970	C 3.0910 -0.9660 2.1990
C -3.0580 0.8760 -1.2320	H 3.4930 -1.9090 2.5790
H -3.2910 0.4960 -2.2290	H 3.6420 -0.1460 2.6660

H -3.6910 1.7420 -1.0260	H 2.0370 -0.9040 2.4750
H -2.0170 1.1960 -1.1870	C 4.7620 -0.9740 0.3650
C -4.8040 -0.6800 -0.3020	H 5.2230 -1.8100 0.8990
H -5.0510 -1.4560 0.4250	H 4.9730 -1.1090 -0.6980
H -5.4590 0.1760 -0.1190	H 5.2310 -0.0470 0.7090
H -4.9900 -1.0490 -1.3140	C 0.4590 0.9670 1.5460
C -0.8310 -3.1660 -0.0710	H 1.6120 1.5370 1.3460
H -0.2560 -3.6560 0.7200	H 0.5630 0.8570 2.6410
H -1.3740 -3.9310 -0.6310	O 0.5730 -0.1030 0.8230
H -0.1470 -2.6450 -0.7440	C -0.5620 1.9720 1.1440
C -2.9110 -2.9420 1.3170	C -0.8110 3.2700 1.5040
H -2.4250 -3.6500 1.9940	C -1.9890 3.6550 0.8080
H -3.5530 -2.2950 1.9150	H -0.2210 3.8700 2.1810
H -3.5150 -3.5120 0.6060	C -2.3760 2.5670 0.0740
C 0.7020 0.5490 -2.1580	H -2.4920 4.6110 0.8450
H -0.0270 0.6750 -2.9650	C -3.5240 2.3150 -0.7850
H 1.6480 0.2180 -2.6050	O -3.7390 1.2440 -1.3160
O 0.1720 -0.4130 -1.2630	H -4.2040 3.1760 -0.9150
H 0.9280 -0.6430 -0.6520	O -1.5030 1.5390 0.2740
C 1.9860 0.8500 1.7190	H -0.9550 -0.7780 0.1450
H 1.1330 1.0220 1.0580	C -2.3300 -1.8530 1.2040
H 1.6100 0.4410 2.6650	C -3.5310 -2.5400 1.1460
H 2.4590 1.8110 1.9380	C -4.2040 -2.6410 -0.0690
C 2.7190 -2.7030 -1.5090	C -3.6710 -2.0590 -1.2120
H 1.9300 -2.1830 -2.0610	C -2.4630 -1.3800 -1.1370
H 3.4780 -3.0490 -2.2140	N -1.8450 -1.3150 0.0610
H 2.2710 -3.5890 -1.0440	H -5.1490 -3.1710 -0.1220
C 0.9240 1.8530 -1.4580	H -3.9310 -2.9810 2.0510
C 1.9550 2.7410 -1.3350	H -4.1840 -2.1120 -2.1650
C 1.4920 3.7690 -0.4600	C -1.5440 -1.6420 2.4600
H 2.9250 2.6660 -1.8050	H -1.9070 -2.3040 3.2470
C 0.2170 3.4240 -0.1050	H -0.4790 -1.8180 2.2910
H 2.0360 4.6420 -0.1270	H -1.6590 -0.6060 2.8000
C -0.7810 3.9700 0.8080	C -1.7960 -0.7340 -2.3070
O -1.8280 3.4020 1.0430	H -1.5390 0.2980 -2.0590
H -0.5190 4.9320 1.2830	H -0.8860 -1.2780 -2.5840
O -0.1280 2.2650 -0.7230	H -2.4730 -0.7200 -3.1610

P(-1267.779271)

C -3.7400 -0.8810 1.1890
C -5.1040 -0.6550 1.3060
C -5.9000 -0.6670 0.1650
C -5.3340 -0.9110 -1.0840
C -3.9700 -1.1350 -1.1820

N	-3.2400	-1.1040	-0.0450
H	-6.9680	-0.4940	0.2490
H	-5.5300	-0.4780	2.2870
H	-5.9410	-0.9350	-1.9820
C	-1.1320	2.0350	0.7850
C	-0.4010	2.1390	-0.5820
C	1.8910	2.1110	0.5420
C	1.0920	2.6150	1.7620
C	-0.1780	1.7920	1.9590
H	-1.6970	2.9600	0.9510
H	-1.8700	1.2240	0.7350
H	1.7390	2.5660	2.6450
H	0.8210	3.6700	1.6350
H	0.0840	0.7270	2.0120
H	-0.6660	2.0550	2.9040
N	0.9170	1.4810	-0.4000
O	1.5340	1.2310	-1.6400
C	2.8670	1.0130	0.9710
H	3.3580	0.5870	0.0930
H	3.6340	1.4340	1.6290
H	2.3480	0.2140	1.5120
C	2.7230	3.2520	-0.0680
H	2.1160	4.0530	-0.4950
H	3.3350	3.6960	0.7220
H	3.4150	2.8800	-0.8310
C	-1.1880	1.3380	-1.6240
H	-2.2240	1.6960	-1.6430
H	-0.7630	1.4400	-2.6260
H	-1.1910	0.2790	-1.3520
C	-0.3440	3.5980	-1.0700
H	-1.3520	3.9290	-1.3400
H	0.0320	4.2860	-0.3100
H	0.2760	3.6990	-1.9690
C	0.5330	-1.4560	-0.5200
H	0.6350	-0.8710	-1.4440
O	-0.5530	-1.6750	0.0140
C	-2.7870	-0.8900	2.3450
H	-1.8030	-1.2570	2.0430
H	-2.6730	0.1240	2.7440
H	-3.1770	-1.5240	3.1450
C	-3.2470	-1.4010	-2.4670
H	-3.0530	-0.4580	-2.9900
H	-2.2900	-1.8950	-2.2870
H	-3.8560	-2.0320	-3.1180
C	1.7620	-1.9810	0.0340
C	2.0310	-2.7280	1.1550

C	3.4370	-2.8780	1.1880
H	1.3000	-3.1060	1.8560
C	3.9130	-2.2120	0.0840
H	4.0310	-3.4000	1.9250
C	5.2870	-1.9550	-0.3910
O	5.5240	-1.2380	-1.3290
H	6.0780	-2.4590	0.1930
O	2.9030	-1.6810	-0.6230
H	-2.2160	-1.2730	-0.1230
H	1.8050	2.0890	-2.0190

Hub[1]-TEMPO

R(-1613.191992)				TS(-1613.178095)			
C	1.1850	-2.9190	-0.6090	C	-2.1130	1.9460	1.2580
C	1.2940	-4.2840	-0.8770	C	-3.3390	1.0320	1.0620
C	2.5550	-4.8440	-1.0300	C	-2.6680	0.6210	-1.4440
C	3.6750	-4.0300	-0.9130	C	-2.0450	2.0140	-1.2810
C	3.4980	-2.6750	-0.6450	C	-1.2480	2.1760	0.0030
N	2.2740	-2.1400	-0.4970	H	-2.4720	2.9280	1.5830
H	2.6660	-5.9050	-1.2330	H	-1.4940	1.5380	2.0620
H	0.3970	-4.8890	-0.9570	H	-1.3840	2.2000	-2.1350
H	4.6760	-4.4340	-1.0210	H	-2.8130	2.7950	-1.3110
C	0.5260	1.1320	-1.6580	H	-0.4100	1.4610	-0.0020
C	1.6890	2.1530	-1.6840	N	-3.0730	0.1070	-0.0880
C	0.5520	3.4410	0.3740	O	-3.7090	-0.9810	-0.1030
C	-0.7070	2.7800	-0.1940	C	-1.6780	-0.3680	-2.0600
C	-0.4250	1.3030	-0.4670	H	-2.0920	-1.3820	-2.0540
H	-0.0260	1.2340	-2.5990	H	-1.4970	-0.0820	-3.1000
H	0.9480	0.1220	-1.6250	H	-0.7350	-0.3700	-1.5150
H	-1.4890	2.8580	0.5680	C	-3.9200	0.6810	-2.3310
H	-1.0540	3.3060	-1.0910	H	-3.6340	1.0830	-3.3080
H	0.0910	0.9100	0.4110	H	-4.3380	-0.3180	-2.4720
N	1.7750	2.8510	-0.3380	H	-4.6890	1.3310	-1.9100
O	2.8380	3.0310	0.1450	C	-3.6010	0.2080	2.3210
C	0.7420	3.1570	1.8660	H	-3.7700	0.9050	3.1460
H	1.6380	3.6490	2.2480	H	-4.4880	-0.4200	2.2160
H	-0.1330	3.5560	2.3850	H	-2.7370	-0.4120	2.5680
H	0.7910	2.0860	2.0630	C	-4.6140	1.8360	0.7470
C	0.6100	4.9550	0.1160	H	-4.8960	2.4000	1.6410
H	0.5020	5.2100	-0.9400	H	-4.4770	2.5540	-0.0630
H	-0.2280	5.4000	0.6600	H	-5.4360	1.1600	0.4940
H	1.5400	5.3790	0.5050	C	-1.6960	-2.0320	0.8150
C	3.0370	1.5030	-1.9600	H	-2.8720	-1.8830	0.2910

H 2.9340 0.9220 -2.8800	H -2.0390 -2.4180 1.7930
H 3.8280 2.2430 -2.1000	O -1.2390 -0.8190 0.7820
H 3.3120 0.8310 -1.1430	C -1.0410 -3.0710 -0.0280
C 1.4280 3.2950 -2.6910	C -1.4340 -4.3150 -0.4430
H 1.5210 2.8540 -3.6870	C -0.3360 -4.8550 -1.1640
H 0.4260 3.7190 -2.6040	H -2.3910 -4.7790 -0.2550
H 2.1770 4.0880 -2.5970	C 0.6470 -3.9050 -1.1380
C 2.3180 -0.3440 2.2060	H -0.2720 -5.8240 -1.6400
H 2.6100 0.4490 2.9030	C 2.0110 -3.9100 -1.6490
H 3.0520 -1.1560 2.2920	O 2.7970 -2.9990 -1.4870
O 2.3120 0.2230 0.9060	H 2.2880 -4.8270 -2.1970
H 2.2260 -0.5550 0.2840	O 0.2170 -2.8100 -0.4470
C -0.1560 -2.2570 -0.4630	H 0.5570 -0.6310 0.7400
H -0.0880 -1.3640 0.1600	C 1.8080 -0.9680 2.3200
H -0.5480 -1.9430 -1.4370	C 3.1250 -1.1260 2.7110
H -0.8900 -2.9340 -0.0190	C 4.1390 -1.0230 1.7580
C 4.6710 -1.7380 -0.5320	C 3.8290 -0.7380 0.4370
H 4.4800 -0.9630 0.2160	C 2.5020 -0.5530 0.0660
H 5.5830 -2.2750 -0.2630	N 1.5580 -0.6850 1.0190
H 4.8500 -1.2390 -1.4910	H 5.1740 -1.1680 2.0540
C 0.9640 -0.8750 2.5610	H 3.3470 -1.3460 3.7480
C 0.4580 -2.0840 2.9450	H 4.5970 -0.6550 -0.3230
C -0.9580 -1.9220 3.0030	C 0.6350 -1.1260 3.2370
H 1.0270 -2.9810 3.1460	H 0.9580 -1.0500 4.2760
C -1.2110 -0.6270 2.6440	H -0.1310 -0.3740 3.0340
H -1.6960 -2.6690 3.2610	H 0.1780 -2.1110 3.0910
C -2.4320 0.1450 2.4390	C 2.0610 -0.1810 -1.3080
O -2.4320 1.3110 2.1030	H 1.3350 -0.9100 -1.6740
H -3.3700 -0.4150 2.6020	H 1.6120 0.8190 -1.2970
O -0.0400 0.0120 2.3930	H 2.9150 -0.1730 -1.9850
O -1.5600 0.4880 -0.6000	O -0.7460 3.4990 0.0010
C -2.6530 0.9940 -1.3560	C 0.4380 3.6720 0.7510
H -2.9820 1.9580 -0.9490	H 0.3950 3.1170 1.7000
H -2.3650 1.1420 -2.4080	H 0.4800 4.7380 1.0000
C -3.7680 -0.0090 -1.2450	C 1.6740 3.2840 -0.0330
C -3.8940 -1.0440 -2.1700	C 1.7160 3.5070 -1.4130
C -4.6560 0.0630 -0.1700	C 2.8030 2.7760 0.6090
C -4.9000 -1.9970 -2.0280	C 2.8720 3.2320 -2.1360
H -3.2070 -1.1000 -3.0120	H 0.8360 3.9080 -1.9070
C -5.6620 -0.8870 -0.0260	C 3.9670 2.5160 -0.1130
H -4.5510 0.8700 0.5520	H 2.7800 2.6020 1.6830
C -5.7840 -1.9180 -0.9550	C 4.0040 2.7390 -1.4870
H -4.9990 -2.7940 -2.7580	H 2.8970 3.4150 -3.2060
H -6.3580 -0.8180 0.8050	H 4.8480 2.1440 0.4020
H -6.5730 -2.6560 -0.8480	H 4.9120 2.5420 -2.0470

P(-1613.213733)

C	-2.3250	2.0860	-1.0280
C	-3.6860	2.2970	-0.8470
C	-4.1160	3.2100	0.1090
C	-3.1910	3.9160	0.8770
C	-1.8380	3.6910	0.6870
N	-1.4720	2.7880	-0.2490
H	-5.1780	3.3810	0.2540
H	-4.3870	1.7340	-1.4540
H	-3.5110	4.6400	1.6180
C	-0.6500	-0.5370	1.2210
C	0.5350	-0.2950	2.1900
C	1.9780	-2.0220	0.9710
C	0.6130	-2.5640	0.4860
C	-0.2650	-1.4080	0.0220
H	-1.4740	-1.0100	1.7690
H	-1.0320	0.4200	0.8450
H	0.7770	-3.2690	-0.3350
H	0.1100	-3.1150	1.2910
H	0.3170	-0.7960	-0.6770
N	1.7670	-0.6110	1.4200
O	2.9130	-0.1360	2.0790
C	2.9650	-1.9710	-0.2000
H	3.8970	-1.4990	0.1220
H	3.1880	-2.9860	-0.5420
H	2.5520	-1.4050	-1.0420
C	2.5810	-2.9500	2.0380
H	1.9890	-3.0060	2.9540
H	2.6390	-3.9620	1.6260
H	3.6050	-2.6540	2.2890
C	0.5680	1.1930	2.5570
H	-0.3850	1.4590	3.0300
H	1.3780	1.4250	3.2510
H	0.6860	1.8010	1.6560
C	0.3560	-1.0960	3.4920
H	-0.4720	-0.6720	4.0690
H	0.1210	-2.1470	3.3090
H	1.2480	-1.0430	4.1270
C	2.2900	1.7280	-0.3520
H	2.6220	1.7680	0.6950
O	1.1920	2.1360	-0.7190
C	-1.7530	1.1200	-2.0120
H	-0.6750	1.2650	-2.1250
H	-1.9370	0.0900	-1.6760
H	-2.2370	1.2500	-2.9830

C	-0.7500	4.3800	1.4530
H	-0.6880	3.9750	2.4680
H	0.2210	4.2460	0.9710
H	-0.9620	5.4490	1.5300
C	3.2410	1.1720	-1.2930
C	3.1930	0.9680	-2.6500
C	4.4120	0.3330	-2.9850
H	2.3780	1.2410	-3.3040
C	5.1050	0.2010	-1.8050
H	4.7440	0.0050	-3.9600
C	6.3960	-0.4410	-1.4950
O	6.7940	-0.5870	-0.3670
H	6.9600	-0.7920	-2.3780
O	4.4070	0.7190	-0.7830
H	-0.4590	2.6060	-0.3700
H	3.0400	-0.6740	2.8840
O	-1.4040	-1.7830	-0.7330
C	-2.2810	-2.7520	-0.1780
H	-2.0500	-3.7420	-0.5930
H	-2.1530	-2.8140	0.9110
C	-3.7020	-2.3620	-0.4970
C	-4.1860	-1.1200	-0.0750
C	-4.5510	-3.2180	-1.1930
C	-5.5020	-0.7500	-0.3330
H	-3.5250	-0.4490	0.4720
C	-5.8680	-2.8470	-1.4600
H	-4.1800	-4.1820	-1.5320
C	-6.3470	-1.6150	-1.0300
H	-5.8790	0.2040	0.0260
H	-6.5190	-3.5230	-2.0050
H	-7.3750	-1.3300	-1.2300

Hub[6]-TEMPO

R(-1667.369622)	TS(-1667.345161)
C 1.8260 -2.3000 -1.5330	C 0.3420 2.6920 1.1530
C 1.8310 -3.0630 -2.6960	C -1.0440 3.3350 0.9070
C 2.8070 -2.8210 -3.6580	C -1.1360 2.2790 -1.5020
C 3.7530 -1.8350 -3.4270	C 0.3910 2.3030 -1.3350
C 3.6880 -1.1000 -2.2410	C 0.7980 1.7560 0.0330
N 2.7340 -1.3270 -1.3270	H 1.0690 3.5070 1.2650
H 2.8300 -3.4040 -4.5740	H 0.3340 2.1410 2.0980
H 1.0830 -3.8360 -2.8390	H 0.8300 1.6790 -2.1200
H 4.5390 -1.6280 -4.1460	H 0.7880 3.3170 -1.4650
C -0.0940 1.0770 -0.9870	H 0.3170 0.7860 0.1660

C 0.6380 2.4180 -1.1860	N -1.7710 2.5610 -0.1610
C 0.1150 3.0450 1.3430	O -3.0240 2.7020 -0.1910
C -1.1500 2.2410 1.0480	C -1.6030 0.9030 -1.9740
C -0.8450 0.9080 0.3520	H -2.6880 0.8040 -1.8710
H -0.8060 1.0040 -1.8110	H -1.3450 0.7870 -3.0310
H 0.6390 0.2740 -1.0930	H -1.1140 0.1140 -1.4040
H -1.6210 2.0280 2.0130	C -1.6160 3.3370 -2.5020
H -1.8710 2.8150 0.4640	H -1.1250 3.1570 -3.4630
H -0.2060 0.3230 1.0210	H -2.6970 3.2650 -2.6430
N 1.0690 2.9480 0.1570	H -1.3710 4.3520 -2.1820
O 2.1570 3.3930 0.2850	C -1.8730 3.3200 2.1880
C 0.8630 2.5070 2.5660	H -1.3330 3.8960 2.9450
H 1.8390 2.9850 2.6770	H -2.8510 3.7820 2.0390
H 0.2590 2.7450 3.4440	H -1.9960 2.3000 2.5580
H 0.9840 1.4240 2.5040	C -0.9150 4.7920 0.4300
C -0.1710 4.5470 1.5010	H -0.5280 5.3970 1.2560
H -0.6370 4.9690 0.6070	H -0.2250 4.9020 -0.4100
H -0.8720 4.6580 2.3320	H -1.8930 5.1880 0.1430
H 0.7440 5.0960 1.7360	C -3.1100 0.5490 0.9800
C 1.8800 2.2620 -2.0490	H -3.5000 1.6060 0.3590
H 1.5750 1.7590 -2.9710	H -3.5500 0.7820 1.9680
H 2.3250 3.2260 -2.3060	O -1.8220 0.6390 0.8680
H 2.6220 1.6450 -1.5380	C -3.7850 -0.5900 0.2930
C -0.2970 3.5020 -1.7730	C -5.0690 -0.8360 -0.1080
H -0.4580 3.2190 -2.8180	C -5.0820 -2.1690 -0.6030
H -1.2780 3.5260 -1.3010	H -5.8990 -0.1470 -0.0520
H 0.1780 4.4870 -1.7540	C -3.8060 -2.6430 -0.4720
C 3.1700 0.0280 1.7560	H -5.9260 -2.7140 -1.0020
H 3.2660 0.8240 2.5030	C -3.2080 -3.9440 -0.7390
H 4.1860 -0.2860 1.4810	O -2.0420 -4.2020 -0.5220
O 2.4630 0.5600 0.6580	H -3.9060 -4.6940 -1.1530
H 2.5100 -0.1370 -0.0590	O -3.0100 -1.6770 0.0720
C 0.8410 -2.5600 -0.4260	H -0.9250 -1.2750 0.4090
H 0.3810 -1.6350 -0.0650	C 0.2940 -1.9780 1.8790
H 0.0480 -3.2360 -0.7540	C 1.3560 -2.8140 2.1640
H 1.3580 -3.0210 0.4240	C 1.9340 -3.5630 1.1390
C 4.7110 -0.0370 -1.9360	C 1.4440 -3.4700 -0.1530
H 4.4960 0.4440 -0.9790	C 0.3860 -2.6100 -0.4270
H 5.7120 -0.4750 -1.8850	N -0.1360 -1.9090 0.5970
H 4.7310 0.7280 -2.7190	H 2.7650 -4.2260 1.3580
C 2.4600 -1.1500 2.3530	H 1.7160 -2.8830 3.1840
C 2.8810 -2.3100 2.9430	H 1.8740 -4.0420 -0.9650
C 1.7040 -3.0060 3.3330	C -0.4390 -1.1650 2.8990
H 3.9060 -2.6250 3.0750	H -0.4350 -0.1050 2.6290
C 0.6510 -2.2250 2.9390	H -1.4870 -1.4800 2.9480
H 1.6380 -3.9700 3.8170	H 0.0130 -1.3000 3.8820

C -0.7810 -2.4640 2.9160	C -0.1540 -2.3960 -1.8010
O -1.5730 -1.7410 2.3400	H -1.1940 -2.0680 -1.7700
H -1.1120 -3.3700 3.4510	H 0.4590 -1.6340 -2.2920
O 1.1130 -1.0860 2.3540	H -0.1020 -3.3280 -2.3660
N -2.0510 0.1280 0.1740	N 2.2250 1.5050 0.1350
H -2.1430 -0.6960 0.7530	H 2.8430 2.2790 0.3390
C -3.0780 0.5700 -0.6020	C 2.7530 0.3790 -0.4210
O -2.9810 1.6090 -1.2540	O 2.0360 -0.4580 -0.9650
C -4.3100 -0.2790 -0.6390	C 4.2350 0.2090 -0.3270
C -4.5640 -1.2980 0.2850	C 5.0070 0.8460 0.6480
C -5.2390 -0.0070 -1.6450	C 4.8450 -0.6390 -1.2520
C -5.7350 -2.0420 0.1900	C 6.3830 0.6460 0.6840
H -3.8770 -1.5120 1.0990	H 4.5370 1.4690 1.4050
C -6.4050 -0.7570 -1.7400	C 6.2210 -0.8310 -1.2190
H -5.0270 0.7990 -2.3400	H 4.2250 -1.1350 -1.9920
C -6.6530 -1.7760 -0.8240	C 6.9910 -0.1860 -0.2530
H -5.9340 -2.8260 0.9120	H 6.9800 1.1350 1.4480
H -7.1220 -0.5450 -2.5270	H 6.6950 -1.4810 -1.9470
H -7.5660 -2.3600 -0.8940	H 8.0660 -0.3360 -0.2260

P(-1667.384976)

C -2.3650 2.9870 -0.8710
C -3.3500 3.9510 -0.6910
C -2.9880 5.2880 -0.5710
C -1.6480 5.6620 -0.6370
C -0.6780 4.6890 -0.8140
N -1.0810 3.4030 -0.9150
H -3.7540 6.0450 -0.4350
H -4.3880 3.6420 -0.6550
H -1.3480 6.7010 -0.5600
C -0.2860 0.5490 1.7040
C 1.0510 1.1480 2.2120
C 2.2210 -1.1140 2.0680
C 0.7820 -1.6540 2.2100
C -0.1480 -0.9130 1.2520
H -1.0390 0.6100 2.5000
H -0.6610 1.1350 0.8570
H 0.7840 -2.7270 1.9870
H 0.4160 -1.5420 3.2390
H 0.2790 -0.9370 0.2460
N 2.1160 0.3050 1.6150
O 3.3700 0.9340 1.6930
C 2.9440 -1.8840 0.9580
H 3.8500 -1.3530 0.6530
H 3.2240 -2.8800 1.3160
H 2.2940 -2.0090 0.0860

C	3.0140	-1.3090	3.3690
H	2.6370	-0.7160	4.2060
H	2.9540	-2.3610	3.6630
H	4.0760	-1.0830	3.2200
C	1.2120	2.5690	1.6620
H	0.3180	3.1530	1.9140
H	2.0860	3.0690	2.0860
H	1.3240	2.5400	0.5760
C	1.0670	1.2510	3.7490
H	0.3560	2.0210	4.0660
H	0.7830	0.3210	4.2450
H	2.0530	1.5570	4.1200
C	1.8210	0.8860	-1.3590
H	2.6600	1.4150	-0.8810
O	0.6930	1.3650	-1.4240
C	-2.6370	1.5230	-1.0210
H	-1.7460	0.9570	-1.3050
H	-3.0080	1.1130	-0.0750
H	-3.4120	1.3640	-1.7750
C	0.7910	4.9660	-0.8910
H	1.2150	5.0380	0.1170
H	1.3160	4.1700	-1.4250
H	0.9710	5.9140	-1.4020
C	2.1220	-0.4110	-1.9210
C	1.3200	-1.4000	-2.4390
C	2.1980	-2.4610	-2.7640
H	0.2400	-1.3630	-2.5100
C	3.4590	-2.0360	-2.4200
H	1.9440	-3.4230	-3.1880
C	4.7700	-2.7100	-2.4790
O	5.7810	-2.2070	-2.0580
H	4.7460	-3.7180	-2.9310
O	3.4200	-0.7920	-1.9140
H	-0.3490	2.6800	-1.0560
H	3.6380	0.9460	2.6320
N	-1.4390	-1.5660	1.1370
H	-1.9190	-1.8350	1.9850
C	-2.0530	-1.7760	-0.0580
O	-1.5960	-1.3450	-1.1150
C	-3.3400	-2.5420	-0.0130
C	-3.6620	-3.4220	1.0230
C	-4.2300	-2.3710	-1.0750
C	-4.8730	-4.1050	1.0090
H	-2.9560	-3.6150	1.8270
C	-5.4440	-3.0490	-1.0850
H	-3.9470	-1.7110	-1.8890

C -5.7680 -3.9130 -0.0410<br>H -5.1130 -4.7960 1.8100<br>H -6.1360 -2.9100 -1.9100<br>H -6.7120 -4.4480 -0.0520

Hub^2-TEMPO

R(-1593.31832)	TS(-1593.307652)
C 0.4900 -2.3760 1.7890	C -0.1570 -2.5280 -0.3770
C 0.9710 -3.3940 2.6100	C -1.6520 -2.9160 -0.3250
C 0.2680 -3.7150 3.7630	C -1.9520 -1.4760 1.8400
C -0.8940 -3.0150 4.0620	C -0.4630 -1.8230 2.0120
C -1.3180 -2.0090 3.1980	C 0.2930 -1.5500 0.7170
N -0.6290 -1.6960 2.0870	H 0.4420 -3.4410 -0.2770
H 0.6210 -4.5020 4.4220	H 0.0650 -2.0950 -1.3590
H 1.8820 -3.9190 2.3420	H -0.0810 -1.1990 2.8270
H -1.4710 -3.2410 4.9530	H -0.3170 -2.8630 2.3220
C 0.9950 1.9140 1.3210	H 0.0110 -0.5310 0.4130
C -0.0680 2.9920 1.6350	N -2.3800 -1.8540 0.4430
C -0.4250 3.3370 -0.9790	O -3.5900 -1.6600 0.1790
C 1.0390 2.8890 -1.0070	C -2.1970 0.0220 2.0290
C 1.2450 1.6290 -0.1660	H -3.2320 0.2790 1.7830
H 1.9360 2.2580 1.7650	H -2.0310 0.2700 3.0810
H 0.7010 0.9840 1.8200	H -1.5320 0.6240 1.4090
H 1.2840 2.6890 -2.0550	C -2.8320 -2.2470 2.8340
H 1.7200 3.6780 -0.6750	H -2.5330 -1.9700 3.8490
H 0.4870 0.8980 -0.4950	H -3.8830 -1.9830 2.6960
N -0.9730 3.1540 0.4360	H -2.7210 -3.3290 2.7320
O -2.1400 3.2510 0.5970	C -2.2650 -3.0280 -1.7180
C -1.3140 2.5010 -1.9060	H -1.6570 -3.7260 -2.3010
H -2.3550 2.8250 -1.8510	H -3.2850 -3.4160 -1.6720
H -0.9600 2.6620 -2.9270	H -2.2720 -2.0620 -2.2220
H -1.2520 1.4350 -1.6740	C -1.8650 -4.2670 0.3850
C -0.5960 4.8320 -1.2950	H -1.4730 -5.0570 -0.2630
H -0.0100 5.4710 -0.6310	H -1.3450 -4.3390 1.3400
H -0.2360 4.9860 -2.3160	H -2.9330 -4.4520 0.5410
H -1.6490 5.1210 -1.2500	C -2.9430 0.2810 -1.2170
C -0.9240 2.6370 2.8400	H -3.6550 -0.5450 -0.5470
H -0.2450 2.4460 3.6750	H -3.3730 0.0580 -2.2120
H -1.5970 3.4510 3.1180	O -1.7400 -0.1430 -0.9690
H -1.5010 1.7350 2.6320	C -3.3210 1.6230 -0.6960
C 0.5640 4.3890 1.8380	C -4.5000 2.3180 -0.6500
H 1.1050 4.3430 2.7870	C -4.2060 3.5480 -0.0020

H 1.2790 4.6570 1.0610	H -5.4560 1.9830 -1.0260
H -0.2040 5.1620 1.9220	C -2.8710 3.5130 0.2980
C -2.8410 0.1560 0.1390	H -4.8890 4.3560 0.2200
H -3.3190 1.1010 -0.1570	C -1.9950 4.4590 0.9780
H -3.4840 -0.2900 0.9100	O -0.8170 4.2540 1.1850
O -1.5580 0.4600 0.6330	H -2.4900 5.3920 1.3020
H -1.2040 -0.3520 1.1040	O -2.3290 2.3410 -0.1300
C 1.1930 -2.0170 0.5080	H -0.2480 0.5720 -1.5570
H 0.9380 -0.9950 0.2120	C 1.1260 -0.1400 -2.9060
H 2.2800 -2.1060 0.5980	C 2.4780 -0.2360 -3.2020
H 0.8740 -2.6860 -0.3000	C 3.4000 0.4430 -2.4100
C -2.5930 -1.2510 3.4510	C 2.9680 1.2320 -1.3490
H -2.5110 -0.2220 3.0930	C 1.6110 1.3160 -1.0680
H -3.4260 -1.7270 2.9210	N 0.7570 0.6090 -1.8440
H -2.8430 -1.2370 4.5140	H 4.4630 0.3630 -2.6190
C -2.7670 -0.7630 -1.0390	H 2.7920 -0.8390 -4.0460
C -1.7180 -1.2900 -1.7380	H 3.6750 1.7650 -0.7210
C -2.3020 -2.1010 -2.7560	C 0.0430 -0.7870 -3.7140
H -0.6690 -1.1190 -1.5420	H 0.2860 -1.8320 -3.9200
C -3.6530 -2.0030 -2.5960	H -0.9090 -0.7370 -3.1840
H -1.7920 -2.6840 -3.5110	H -0.0580 -0.2700 -4.6740
C -4.7710 -2.6000 -3.3320	C 1.0200 2.1790 -0.0030
O -5.9310 -2.4010 -3.0640	H 1.7370 2.3330 0.8050
H -4.4600 -3.2570 -4.1660	H 0.7640 3.1620 -0.4150
O -3.9380 -1.1850 -1.5460	H 0.0920 1.7620 0.3950
N 2.5930 1.1130 -0.3190	N 1.7410 -1.6500 0.8750
H 2.7920 0.4640 0.4420	H 2.1560 -1.4450 -0.0360
C 2.8120 0.4050 -1.5840	C 2.2820 -0.6920 1.8440
H 2.9490 1.1470 -2.3800	H 2.2980 -1.1650 2.8340
H 1.9360 -0.2080 -1.8660	H 1.6370 0.2000 1.9370
C 4.0300 -0.4820 -1.4920	C 3.6740 -0.2310 1.4750
C 3.9760 -1.8080 -1.9190	C 4.1180 1.0300 1.8850
C 5.2270 0.0190 -0.9710	C 4.5240 -1.0260 0.7030
C 5.1010 -2.6250 -1.8380	C 5.3710 1.5030 1.5040
H 3.0460 -2.2040 -2.3240	H 3.4750 1.6470 2.5100
C 6.3500 -0.7980 -0.8840	C 5.7740 -0.5510 0.3110
H 5.2700 1.0510 -0.6350	H 4.2080 -2.0240 0.4130
C 6.2900 -2.1200 -1.3190	C 6.1970 0.7180 0.7000
H 5.0480 -3.6540 -2.1790	H 5.7010 2.4830 1.8320
H 7.2760 -0.4000 -0.4820	H 6.4250 -1.1810 -0.2880
H 7.1690 -2.7540 -1.2570	H 7.1720 1.0860 0.3970

P(-1593.354013)

C -2.3340 -0.8030 -1.6250
C -3.7080 -0.6210 -1.5370

C	-4.2760	0.5310	-2.0700
C	-3.4810	1.4820	-2.7050
C	-2.1120	1.2810	-2.7870
N	-1.6020	0.1590	-2.2310
H	-5.3490	0.6820	-1.9930
H	-4.3150	-1.3740	-1.0440
H	-3.9110	2.3760	-3.1430
C	-0.3330	2.1940	0.4800
C	1.1630	2.6010	0.4070
C	1.7990	0.7250	2.0260
C	0.3160	0.8280	2.4310
C	-0.5650	0.8580	1.1870
H	-0.9020	2.9570	1.0230
H	-0.7550	2.1460	-0.5340
H	0.0760	-0.0240	3.0760
H	0.1180	1.7330	3.0200
H	-0.2280	0.0400	0.5190
N	1.9510	1.3740	0.6920
O	3.3130	1.5630	0.3990
C	2.1820	-0.7470	1.8330
H	3.1960	-0.8160	1.4290
H	2.1530	-1.2760	2.7910
H	1.4940	-1.2420	1.1390
C	2.7000	1.3080	3.1250
H	2.5640	2.3820	3.2690
H	2.4580	0.8200	4.0740
H	3.7570	1.1050	2.9230
C	1.4870	3.0410	-1.0230
H	0.8580	3.8980	-1.2890
H	2.5330	3.3360	-1.1330
H	1.2730	2.2270	-1.7240
C	1.4570	3.7820	1.3490
H	0.9630	4.6830	0.9710
H	1.0910	3.6100	2.3630
H	2.5300	4.0040	1.3950
C	2.2670	-0.2710	-1.7110
H	2.7360	0.7200	-1.7090
O	1.1080	-0.4570	-2.0680
C	-1.6090	-1.9930	-1.0820
H	-0.6100	-2.0780	-1.5170
H	-1.5050	-1.8960	0.0030
H	-2.1820	-2.9010	-1.2820
C	-1.1610	2.2290	-3.4480
H	-0.9980	3.1070	-2.8130
H	-0.1930	1.7560	-3.6330
H	-1.5740	2.5740	-4.3990

C 3.1250 -1.3620 -1.2870<br>C 2.9330 -2.7190 -1.1970<br>C 4.1400 -3.2440 -0.6790<br>H 2.0370 -3.2550 -1.4730<br>C 4.9730 -2.1660 -0.4970<br>H 4.3780 -4.2770 -0.4670<br>C 6.3510 -2.0750 0.0240<br>O 6.9330 -1.0280 0.1510<br>H 6.8020 -3.0470 0.2940<br>O 4.3650 -1.0280 -0.8690<br>H -0.5750 0.0320 -2.2580<br>H 3.6770 2.1930 1.0510<br>N -1.9820 0.7320 1.5050<br>H -2.5150 0.8040 0.6390<br>C -2.3500 -0.5140 2.1860<br>H -2.3250 -0.3460 3.2700<br>H -1.6260 -1.3240 1.9870<br>C -3.7270 -0.9730 1.7660<br>C -3.9860 -2.3220 1.5120<br>C -4.7590 -0.0430 1.5980<br>C -5.2470 -2.7370 1.0840<br>H -3.1970 -3.0560 1.6560<br>C -6.0170 -0.4530 1.1630<br>H -4.5680 1.0050 1.8120<br>C -6.2630 -1.8010 0.8980<br>H -5.4360 -3.7890 0.8990<br>H -6.8110 0.2780 1.0460<br>H -7.2460 -2.1220 0.5680

Hub^{8}-TEMPO

R(-1898.332493)	TS(-1898.308007)
C -2.3000 2.7680 -1.3460<br>C -2.0500 3.6630 -2.3810<br>C -2.9840 3.7910 -3.4040<br>C -4.1430 3.0310 -3.3580<br>C -4.3300 2.1460 -2.2940<br>N -3.4170 2.0170 -1.3210<br>H -2.8110 4.4840 -4.2210<br>H -1.1390 4.2520 -2.3780<br>H -4.9020 3.1150 -4.1290<br>C -1.3350 -1.1040 -1.0500<br>C -2.3610 -2.1840 -1.4460<br>C -2.2930 -3.1150 1.0400	C 1.3310 -2.7570 1.0780<br>C 2.7850 -3.2310 0.8480<br>C 2.8220 -2.0540 -1.5000<br>C 1.3130 -2.3170 -1.4030<br>C 0.7860 -1.8670 -0.0410<br>H 0.7040 -3.6540 1.1770<br>H 1.2690 -2.2110 2.0230<br>H 0.8170 -1.7480 -2.1950<br>H 1.0790 -3.3750 -1.5660<br>H 1.1390 -0.8480 0.1320<br>N 3.4460 -2.3120 -0.1460<br>O 4.7090 -2.2890 -0.1370

C -0.8380 -2.6630 0.9340	C 3.0910 -0.5980 -1.8860
C -0.7020 -1.2490 0.3520	H 4.1490 -0.3540 -1.7470
H -0.5510 -1.1540 -1.8070	H 2.8440 -0.4670 -2.9440
H -1.8250 -0.1300 -1.1260	H 2.4830 0.0840 -1.2910
H -0.4340 -2.6570 1.9520	C 3.4940 -2.9700 -2.5300
H -0.2340 -3.3660 0.3600	H 2.9860 -2.8450 -3.4900
H -1.2240 -0.5630 1.0250	H 4.5460 -2.7010 -2.6520
N -3.0570 -2.6730 -0.2040	H 3.4340 -4.0230 -2.2470
O -4.2330 -2.8060 -0.2080	C 3.5670 -3.1980 2.1590
C -2.9990 -2.4890 2.2460	H 3.0700 -3.8710 2.8640
H -4.0720 -2.6910 2.2280	H 4.5940 -3.5430 2.0210
H -2.5750 -2.9480 3.1430	H 3.5670 -2.1940 2.5860
H -2.8220 -1.4130 2.2840	C 2.8480 -4.6640 0.2920
C -2.4300 -4.6460 1.0680	H 2.4840 -5.3530 1.0610
H -1.9950 -5.1110 0.1800	H 2.2300 -4.8080 -0.5960
H -1.8800 -5.0040 1.9420	H 3.8810 -4.9320 0.0540
H -3.4780 -4.9420 1.1650	C 4.4310 -0.2100 1.1310
C -3.4160 -1.6390 -2.3940	H 5.0050 -1.1510 0.4660
H -2.8910 -1.1770 -3.2350	H 4.8830 -0.4150 2.1180
H -4.0700 -2.4260 -2.7780	O 3.1830 -0.5150 0.9690
H -4.0190 -0.8790 -1.8940	C 4.9240 1.0550 0.5170
C -1.6780 -3.4320 -2.0530	C 6.1710 1.5420 0.2320
H -1.3260 -3.1250 -3.0420	C 5.9860 2.8700 -0.2360
H -0.8020 -3.7590 -1.4960	H 7.1030 1.0090 0.3490
H -2.3960 -4.2480 -2.1830	C 4.6410 3.1070 -0.2100
C -4.4640 0.5720 1.5550	H 6.7490 3.5690 -0.5530
H -4.8430 -0.2290 2.2010	C 3.8400 4.2850 -0.4990
H -5.3290 1.1750 1.2450	O 2.6310 4.3120 -0.3770
O -3.8200 -0.0370 0.4600	H 4.4140 5.1670 -0.8340
H -3.6140 0.7040 -0.1820	O 3.9870 1.9960 0.2480
C -1.3630 2.6350 -0.1770	H 1.9560 1.8070 0.3600
H -1.1660 1.5870 0.0680	C 0.4080 1.8540 1.6930
H -0.4100 3.1290 -0.3770	C -0.9290 2.1690 1.8470
H -1.8120 3.1010 0.7090	C -1.6490 2.6720 0.7600
C -5.5840 1.3170 -2.1950	C -1.0290 2.8380 -0.4660
H -5.5740 0.7040 -1.2910	C 0.3060 2.4780 -0.6150
H -6.4680 1.9630 -2.1670	N 0.9590 2.0220 0.4660
H -5.6920 0.6600 -3.0640	H -2.6990 2.9220 0.8760
C -3.5290 1.4540 2.3270	H -1.3940 2.0340 2.8160
C -3.6900 2.6190 3.0250	H -1.5710 3.1990 -1.3320
C -2.4130 2.9370 3.5630	C 1.3090 1.3700 2.7840
H -4.6080 3.1790 3.1320	H 2.0210 2.1560 3.0610
C -1.5640 1.9500 3.1420	H 0.7230 1.1150 3.6680
H -2.1460 3.7970 4.1620	H 1.8770 0.4940 2.4530
C -0.1230 1.8050 3.2330	C 1.0140 2.5000 -1.9270
O 0.5090 0.9610 2.6260	H 2.0790 2.2900 -1.8060

H 0.3790 2.5380 3.8890<br>O -2.2490 1.0380 2.4000<br>N 0.6800 -0.8210 0.3410<br>H 0.9340 -0.1090 1.0130<br>C 1.6170 -1.4670 -0.4050<br>O 1.3020 -2.3950 -1.1520<br>C 3.0260 -0.9830 -0.3000<br>C 3.4650 -0.0620 0.6570<br>C 3.9410 -1.4960 -1.2220<br>C 4.7930 0.3430 0.6750<br>H 2.7900 0.3480 1.4050<br>C 5.2660 -1.0860 -1.2020<br>H 3.5900 -2.2240 -1.9460<br>C 5.7140 -0.1560 -0.2550<br>H 5.1200 1.0750 1.4070<br>H 5.9720 -1.5070 -1.9110<br>C 7.1270 0.2920 -0.2360<br>C 7.7890 0.5210 0.9760<br>C 7.8220 0.5020 -1.4330<br>C 9.1110 0.9500 0.9910<br>H 7.2710 0.3340 1.9120<br>C 9.1440 0.9340 -1.4170<br>H 7.3140 0.3520 -2.3810<br>C 9.7920 1.1600 -0.2060<br>H 9.6130 1.1130 1.9390<br>H 9.6660 1.1010 -2.3540<br>H 10.8250 1.4950 -0.1940	H 0.5730 1.7330 -2.5770<br>H 0.9110 3.4830 -2.3930<br>N -0.6650 -1.7990 0.0260<br>H -1.1810 -2.6160 0.3260<br>C -1.3120 -0.7830 -0.6050<br>O -0.6860 0.0530 -1.2600<br>C -2.7870 -0.6830 -0.4320<br>C -3.4740 -1.2720 0.6340<br>C -3.4940 0.1000 -1.3480<br>C -4.8460 -1.1020 0.7630<br>H -2.9420 -1.8420 1.3900<br>C -4.8650 0.2690 -1.2160<br>H -2.9470 0.5680 -2.1600<br>C -5.5660 -0.3330 -0.1610<br>H -5.3710 -1.5790 1.5840<br>H -5.4030 0.8920 -1.9240<br>C -7.0290 -0.1480 -0.0180<br>C -7.8570 -0.1230 -1.1470<br>C -7.6090 0.0090 1.2460<br>C -9.2290 0.0590 -1.0140<br>H -7.4260 -0.2790 -2.1320<br>C -8.9810 0.1950 1.3780<br>H -6.9780 0.0090 2.1310<br>C -9.7940 0.2220 0.2480<br>H -9.8590 0.0650 -1.8980<br>H -9.4140 0.3270 2.3640<br>H -10.8650 0.3660 0.3510

P(-1898.347839)

C 0.5550 3.7680 -0.8910<br>C 0.1480 5.0850 -0.7210<br>C 1.1010 6.0950 -0.6580<br>C 2.4560 5.7920 -0.7710<br>C 2.8460 4.4740 -0.9400<br>N 1.8810 3.5280 -0.9850<br>H 0.7880 7.1260 -0.5290<br>H -0.9110 5.3030 -0.6470<br>H 3.2120 6.5690 -0.7360<br>C 1.3490 0.7360 1.7280<br>C 2.8320 0.6100 2.1650<br>C 2.7300 -1.9370 2.0750<br>C 1.2170 -1.6970 2.2680<br>C 0.7420 -0.6100 1.3060<br>H 0.7590 1.1550 2.5540

H	1.2640	1.4310	0.8850
H	0.6830	-2.6350	2.0810
H	0.9910	-1.4000	3.3010
H	1.0820	-0.8520	0.2960
N	3.3180	-0.6610	1.5700
O	4.7200	-0.7290	1.5880
C	2.9380	-2.9920	0.9820
H	3.9790	-2.9990	0.6500
H	2.6860	-3.9840	1.3710
H	2.2900	-2.7920	0.1230
C	3.3750	-2.4690	3.3660
H	3.3690	-1.7520	4.1880
H	2.8150	-3.3490	3.6970
H	4.4030	-2.8000	3.1850
C	3.6410	1.7570	1.5510
H	3.1640	2.7100	1.8130
H	4.6690	1.7690	1.9190
H	3.6630	1.6610	0.4630
C	2.9740	0.7240	3.6950
H	2.7500	1.7510	4.0000
H	2.2950	0.0660	4.2410
H	4.0000	0.5120	4.0200
C	3.2310	-0.0680	-1.3910
H	4.2310	0.0180	-0.9390
O	2.4540	0.8800	-1.4540
C	-0.3760	2.6000	-0.9770
H	0.1290	1.6850	-1.2970
H	-0.8270	2.4090	0.0040
H	-1.1890	2.8200	-1.6750
C	4.2680	4.0240	-1.0680
H	4.7180	3.9050	-0.0770
H	4.3330	3.0650	-1.5890
H	4.8500	4.7660	-1.6190
C	2.8820	-1.3670	-1.9190
C	1.7060	-1.8750	-2.4180
C	1.9800	-3.2340	-2.7100
H	0.7700	-1.3390	-2.4980
C	3.2950	-3.4430	-2.3650
H	1.3010	-3.9730	-3.1110
C	4.1310	-4.6570	-2.3890
O	5.2570	-4.6820	-1.9600
H	3.6370	-5.5460	-2.8220
O	3.8480	-2.3140	-1.8930
H	2.1810	2.5420	-1.1170
H	4.9990	-0.8350	2.5180
N	-0.7050	-0.5430	1.2150

H -1.2420 -0.4940 2.0700
C -1.3590 -0.5040 0.0220
O -0.7580 -0.4340 -1.0490
C -2.8530 -0.5130 0.0770
C -3.5840 -0.9900 1.1690
C -3.5380 -0.0400 -1.0440
C -4.9720 -0.9760 1.1430
H -3.0820 -1.4280 2.0280
C -4.9250 -0.0190 -1.0630
H -2.9610 0.3030 -1.8970
C -5.6670 -0.4850 0.0310
H -5.5290 -1.3860 1.9810
H -5.4470 0.3780 -1.9290
C -7.1490 -0.4740 0.0050
C -7.8410 -0.7820 -1.1710
C -7.8800 -0.1590 1.1560
C -9.2320 -0.7770 -1.1960
H -7.2860 -1.0570 -2.0640
C -9.2700 -0.1500 1.1300
H -7.3540 0.1060 2.0690
C -9.9500 -0.4590 -0.0460
H -9.7560 -1.0280 -2.1130
H -9.8250 0.1050 2.0280
H -11.0350 -0.4540 -0.0650

Hub[4]-TEMPO

R(-1602.190471)	TS(-1602.16144)
C -2.5370 -1.4220 1.5630	C -1.7140 -0.9790 1.2210
C -3.2670 -2.1630 2.4910	C -1.2240 -2.4110 0.9390
C -4.2410 -1.5230 3.2470	C -0.3100 -1.6930 -1.4130
C -4.4610 -0.1650 3.0570	C -1.4730 -0.6960 -1.3170
C -3.6940 0.5180 2.1150	C -1.5420 0.0010 0.0430
N -2.7540 -0.1080 1.3880	H -2.7710 -1.0430 1.4870
H -4.8260 -2.0790 3.9730	H -1.1700 -0.5770 2.0790
H -3.0720 -3.2240 2.6080	H -1.3340 0.0590 -2.0990
H -5.2200 0.3650 3.6230	H -2.4280 -1.1860 -1.5200
C 0.8970 0.5870 0.9600	H -0.5910 0.5270 0.1950
C 0.9550 2.1270 1.0160	N -0.1080 -2.3480 -0.0690
C 1.6560 2.1640 -1.5480	O 0.7020 -3.3180 -0.0810
C 2.3380 0.8540 -1.1550	C 0.9870 -0.9910 -1.8190
C 1.4180 -0.0650 -0.3390	H 1.8370 -1.6760 -1.7330
H 1.4890 0.2400 1.8090	H 0.9020 -0.6910 -2.8690
H -0.1390 0.2800 1.1240	H 1.1820 -0.1160 -1.1960

H 2.5940 0.3370 -2.0860	C -0.6180 -2.7880 -2.4420
H 3.2730 1.0330 -0.6200	H -0.8080 -2.3150 -3.4100
H 0.5530 -0.2910 -0.9680	H 0.2300 -3.4680 -2.5450
N 0.8240 2.6820 -0.3780	H -1.5020 -3.3670 -2.1680
O 0.1250 3.6160 -0.5690	C -0.6980 -3.0530 2.2190
C 0.6980 1.9880 -2.7290	H -1.5120 -3.0610 2.9480
H 0.1320 2.9040 -2.9150	H -0.3780 -4.0830 2.0480
H 1.3070 1.7720 -3.6100	H 0.1280 -2.4730 2.6350
H 0.0150 1.1530 -2.5620	C -2.3480 -3.2990 0.3760
C 2.6690 3.2830 -1.8410	H -3.0760 -3.4740 1.1730
H 3.3210 3.4820 -0.9870	H -2.8860 -2.8320 -0.4500
H 3.2920 2.9470 -2.6730	H -1.9470 -4.2670 0.0600
H 2.1570 4.2040 -2.1350	C 2.1640 -1.7870 1.1410
C -0.1660 2.7050 1.8670	H 1.7600 -2.8510 0.5140
H -0.1290 2.2030 2.8370	H 2.3550 -2.2540 2.1250
H -0.0480 3.7790 2.0250	O 1.1300 -1.0190 1.0220
H -1.1360 2.5110 1.4040	C 3.4100 -1.3870 0.4300
C 2.3250 2.6410 1.5190	C 4.5760 -2.0450 0.1210
H 2.3660 2.3900 2.5830	C 5.3950 -1.1110 -0.5580
H 3.1720 2.1500 1.0410	H 4.8040 -3.0770 0.3480
H 2.3960 3.7280 1.4200	C 4.6760 0.0570 -0.6170
C -2.5820 1.1780 -1.6820	H 6.3860 -1.2720 -0.9580
H -2.3240 1.8750 -2.4890	C 4.9440 1.3670 -1.1420
H -3.6050 1.4120 -1.3590	O 4.1500 2.2980 -1.0360
O -1.6390 1.3660 -0.6470	H 5.9110 1.5080 -1.6480
H -1.9680 0.8160 0.1220	O 3.4660 -0.1150 -0.0100
C -1.4650 -2.0700 0.7280	H 2.6220 2.1020 -0.1170
H -1.1970 -1.4320 -0.1170	C 1.4730 2.2540 1.5880
H -0.5590 -2.2470 1.3210	C 0.2750 2.7430 2.1000
H -1.8010 -3.0380 0.3440	C -0.6110 3.4010 1.2570
C -3.8810 1.9950 1.8880	C -0.3150 3.5470 -0.0990
H -3.4620 2.2970 0.9250	C 0.8710 3.0300 -0.5960
H -4.9390 2.2660 1.9160	N 1.7080 2.4140 0.2710
H -3.3760 2.5700 2.6720	H -1.5380 3.8060 1.6540
C -2.5530 -0.2310 -2.1880	H 0.0660 2.6240 3.1560
C -3.5030 -1.1540 -2.5230	H -0.9890 4.0670 -0.7710
C -2.7950 -2.3230 -2.9260	C 2.5070 1.5690 2.4200
H -4.5730 -1.0150 -2.4820	H 2.4980 1.9710 3.4340
C -1.4660 -2.0300 -2.7930	H 2.2690 0.5020 2.4590
H -3.2120 -3.2660 -3.2510	H 3.5050 1.6910 1.9930
C -0.2530 -2.8260 -2.8800	C 1.3050 3.1160 -2.0260
O 0.8280 -2.4170 -2.5000	H 1.4170 2.1120 -2.4480
H -0.3750 -3.8400 -3.2980	H 0.5680 3.6650 -2.6110
O -1.3190 -0.7500 -2.3550	H 2.2750 3.6150 -2.1040
N 2.0340 -1.3490 -0.0720	N -2.5630 1.0390 0.0650
H 1.7810 -2.1140 -0.6880	H -2.2770 2.0050 0.0230

C 3.1760 -1.5280 0.6350<br>C 4.8290 -0.7720 1.9560<br>C 4.7770 -2.9200 1.3630<br>H 5.3020 0.0510 2.4890<br>H 5.2130 -3.9150 1.3980<br>N 3.7230 -0.4760 1.2730<br>N 5.4120 -1.9590 2.0480<br>N 3.6730 -2.7790 0.6510	C -3.8940 0.8650 -0.1180<br>C -5.7070 -0.4250 -0.3880<br>C -5.9250 1.7930 -0.3240<br>H -6.1480 -1.4150 -0.4840<br>H -6.5560 2.6790 -0.3660<br>N -4.6280 1.9950 -0.1610<br>N -6.5380 0.6110 -0.4430<br>N -4.3860 -0.3800 -0.2320

P(-1602.206281)

C 3.7520 0.1810 -0.8570
C 5.1340 0.2680 -0.7470
C 5.9090 -0.8730 -0.9150
C 5.3070 -2.0980 -1.1970
C 3.9280 -2.1690 -1.3030
N 3.2190 -1.0320 -1.1220
H 6.9890 -0.8090 -0.8350
H 5.5860 1.2320 -0.5400
H 5.8970 -2.9960 -1.3430
C 0.8590 -0.4240 1.8650
C 0.1200 -1.7850 1.9750
C -2.1610 -0.6430 2.0210
C -1.3380 0.5720 2.4980
C -0.0980 0.7470 1.6170
H 1.4180 -0.2400 2.7920
H 1.5940 -0.4610 1.0530
H -1.9660 1.4680 2.4540
H -1.0230 0.4550 3.5440
H -0.4020 0.7540 0.5660
N -1.2100 -1.5760 1.3450
O -1.8410 -2.7990 1.0670
C -3.1730 -0.1810 0.9680
H -3.6190 -1.0420 0.4640
H -3.9700 0.3930 1.4510
H -2.6940 0.4660 0.2260
C -2.9560 -1.2670 3.1800
H -2.3300 -1.7190 3.9530
H -3.5480 -0.4820 3.6600
H -3.6660 -2.0150 2.8130
C 0.8800 -2.8320 1.1530
H 1.9190 -2.8680 1.5030
H 0.4420 -3.8270 1.2600
H 0.8780 -2.5620 0.0940

C	0.0920	-2.2820	3.4320
H	1.1060	-2.5630	3.7360
H	-0.2600	-1.5250	4.1350
H	-0.5330	-3.1760	3.5400
C	-0.6410	-1.2820	-1.5290
H	-0.8940	-2.3040	-1.2070
O	0.5100	-0.8570	-1.5360
C	2.8190	1.3390	-0.6900
H	1.8130	1.1050	-1.0470
H	2.7470	1.6090	0.3710
H	3.2000	2.2080	-1.2330
C	3.1620	-3.4230	-1.5930
H	2.9560	-3.9630	-0.6630
H	2.2070	-3.1990	-2.0750
H	3.7430	-4.0780	-2.2440
C	-1.7430	-0.4560	-1.9670
C	-1.8060	0.8650	-2.3390
C	-3.1750	1.1240	-2.5890
H	-0.9680	1.5440	-2.3790
C	-3.8340	-0.0580	-2.3480
H	-3.6290	2.0560	-2.8960
C	-5.2690	-0.4040	-2.3800
O	-5.6820	-1.4830	-2.0410
H	-5.9310	0.4090	-2.7270
O	-2.9710	-1.0190	-1.9830
H	2.1870	-1.0880	-1.2210
H	-2.0990	-3.2030	1.9180
N	0.5790	2.0110	1.8510
H	0.8940	2.2260	2.7900
C	0.4990	3.0930	1.0400
C	0.9420	5.2690	0.7080
C	-0.0390	4.0810	-0.9000
H	1.3440	6.2130	1.0710
H	-0.4720	4.0200	-1.8990
N	1.0120	4.2390	1.5330
N	0.4270	5.2670	-0.5270
N	-0.0350	2.9570	-0.1880

Hub[5]-TEMPO

R(-1687.224798)	TS(-1687.200151)
C 1.3550 -2.8390 -0.7210	C -1.0350 -1.7190 1.3000
C 1.5130 -4.1750 -1.0870	C -0.0860 -2.9380 1.2740
C 2.7500 -4.6070 -1.5440	C 0.4550 -2.5330 -1.2660
C 3.7980 -3.6980 -1.6230	C -0.9560 -1.9210 -1.2490

C 3.5740 -2.3760 -1.2430	C -1.1170 -0.9940 -0.0490
N 2.3720 -1.9660 -0.8020	H -2.0280 -2.0700 1.5890
H 2.9000 -5.6440 -1.8300	H -0.6910 -1.0050 2.0540
H 0.6730 -4.8580 -1.0090	H -1.0890 -1.3440 -2.1710
H 4.7800 -4.0020 -1.9680	H -1.7240 -2.6940 -1.2420
C 0.2310 1.0830 -1.4760	H -0.3000 -0.2730 -0.0880
C 1.2060 2.2570 -1.7230	N 0.9000 -2.7510 0.1560
C 0.3230 3.4360 0.5100	O 2.0320 -3.2980 0.2720
C -0.9460 2.6600 0.1480	C 1.4640 -1.6080 -1.9450
C -0.5780 1.2160 -0.1800	H 2.4810 -2.0020 -1.8480
H -0.4450 1.0470 -2.3320	H 1.2240 -1.5480 -3.0100
H 0.8050 0.1510 -1.4470	H 1.4370 -0.6090 -1.5110
H -1.5910 2.6580 1.0320	C 0.4610 -3.8850 -1.9930
H -1.4920 3.1330 -0.6680	H 0.1380 -3.7250 -3.0260
H 0.0390 0.8420 0.6390	H 1.4690 -4.3060 -2.0040
N 1.4560 2.9840 -0.4170	H -0.2200 -4.6020 -1.5330
O 2.5620 3.2880 -0.1320	C 0.6740 -3.0660 2.5900
C 0.8050 3.1650 1.9380	H -0.0610 -3.1510 3.3950
H 1.6880 3.7620 2.1730	H 1.3040 -3.9580 2.6030
H -0.0070 3.4460 2.6120	H 1.2860 -2.1810 2.7750
H 1.0190 2.1070 2.0920	C -0.8620 -4.2460 1.0340
C 0.1760 4.9470 0.2750	H -1.4430 -4.4670 1.9340
H -0.1460 5.1830 -0.7420	H -1.5700 -4.1780 0.2060
H -0.5950 5.3000 0.9650	H -0.1710 -5.0760 0.8610
H 1.1090 5.4690 0.5000	C 2.9110 -1.1130 0.9680
C 2.5480 1.7930 -2.2680	H 2.8580 -2.3550 0.5540
H 2.3440 1.1800 -3.1500	H 3.3410 -1.3350 1.9610
H 3.1810 2.6330 -2.5660	O 1.6630 -0.7770 0.9120
H 3.0740 1.1890 -1.5250	C 3.8350 -0.3870 0.0520
C 0.5810 3.3220 -2.6540	C 5.1290 -0.5530 -0.3640
H 0.5630 2.8770 -3.6530	C 5.4130 0.5470 -1.2210
H -0.4470 3.5690 -2.3860	H 5.7900 -1.3630 -0.0940
H 1.1950 4.2260 -2.6950	C 4.2760 1.3060 -1.2650
C 2.8340 -0.2180 1.8720	H 6.3390 0.7540 -1.7380
H 3.2210 0.5780 2.5160	C 3.9300 2.5630 -1.9150
H 3.6220 -0.9720 1.7520	O 2.8460 3.0970 -1.7810
O 2.4790 0.3760 0.6340	H 4.7260 3.0070 -2.5380
H 2.3460 -0.3940 0.0130	O 3.3130 0.7370 -0.4890
C 0.0270 -2.3140 -0.2490	H 1.2800 1.0150 0.5300
H 0.1550 -1.4170 0.3630	C 1.3460 2.4890 1.9330
H -0.6130 -2.0510 -1.1000	C 1.0170 3.8020 2.2240
H -0.5050 -3.0650 0.3430	C 0.3620 4.5710 1.2650
C 4.6620 -1.3400 -1.3310	C 0.0440 4.0240 0.0300
H 4.5450 -0.5860 -0.5480	C 0.3790 2.7040 -0.2440
H 5.6510 -1.7950 -1.2430	N 1.0020 1.9970 0.7210
H 4.6190 -0.8270 -2.2990	H 0.1070 5.6040 1.4830

C 1.6380 -0.8570 2.5070	H 1.2840 4.2120 3.1910
C 1.3220 -2.0930 2.9970	H -0.4500 4.6080 -0.7370
C -0.0510 -2.0290 3.3810	C 2.0840 1.5790 2.8640
H 1.9850 -2.9440 3.0640	H 2.1920 2.0470 3.8430
C -0.4720 -0.7610 3.0880	H 1.5640 0.6230 2.9670
H -0.6500 -2.8200 3.8110	H 3.0830 1.3710 2.4630
C -1.7440 -0.0490 3.1740	C 0.0670 2.0240 -1.5380
O -1.8770 1.0910 2.7790	H -0.8620 1.4480 -1.4500
H -2.5810 -0.6150 3.6180	H -0.0550 2.7710 -2.3230
O 0.5610 -0.0490 2.5690	H 0.8880 1.3640 -1.8250
O -1.6940 0.3330 -0.1740	O -2.2760 -0.1530 -0.1420
C -2.7070 0.5500 -1.0430	C -3.4980 -0.7390 -0.1800
O -2.7120 1.4650 -1.8350	O -3.6540 -1.9380 -0.1510
C -3.7790 -0.4680 -0.9060	C -4.6020 0.2520 -0.2630
C -3.6920 -1.5050 0.0280	C -4.3770 1.6300 -0.2080
C -4.8920 -0.3610 -1.7420	C -5.9000 -0.2460 -0.3970
C -4.7220 -2.4340 0.1200	C -5.4540 2.5050 -0.2870
H -2.8220 -1.5750 0.6730	H -3.3660 2.0070 -0.0950
C -5.9190 -1.2920 -1.6440	C -6.9720 0.6340 -0.4790
H -4.9350 0.4550 -2.4570	H -6.0470 -1.3200 -0.4350
C -5.8340 -2.3260 -0.7140	C -6.7490 2.0080 -0.4240
H -4.6620 -3.2410 0.8430	H -5.2860 3.5760 -0.2410
H -6.7870 -1.2120 -2.2910	H -7.9810 0.2490 -0.5850
H -6.6380 -3.0520 -0.6370	H -7.5870 2.6960 -0.4870

P(-1687.248879)

C -2.1380 3.3800 -0.9020
C -3.0020 4.4570 -0.7470
C -2.4820 5.7230 -0.5030
C -1.1050 5.9150 -0.4180
C -0.2580 4.8300 -0.5740
N -0.8130 3.6180 -0.8020
H -3.1530 6.5690 -0.3860
H -4.0700 4.2910 -0.8270
H -0.6820 6.8970 -0.2410
C -0.4030 0.2680 1.6420
C 0.8950 0.8380 2.2730
C 2.0970 -1.4010 2.0370
C 0.6590 -1.9570 2.0820
C -0.2130 -1.1600 1.1290
H -1.2060 0.2630 2.3890
H -0.7260 0.8980 0.8050
H 0.6720 -3.0120 1.7900
H 0.2330 -1.9100 3.0910
H 0.2350 -1.1400 0.1320

N	2.0020	0.0460	1.6760
O	3.2430	0.6770	1.8650
C	2.8710	-2.0920	0.9100
H	3.7890	-1.5440	0.6810
H	3.1360	-3.1110	1.2080
H	2.2600	-2.1540	0.0040
C	2.8350	-1.6790	3.3570
H	2.4140	-1.1460	4.2130
H	2.7680	-2.7480	3.5760
H	3.9000	-1.4380	3.2710
C	1.0750	2.2960	1.8420
H	0.1620	2.8560	2.0810
H	1.9170	2.7660	2.3550
H	1.2510	2.3490	0.7660
C	0.8250	0.8280	3.8120
H	0.0900	1.5690	4.1430
H	0.5230	-0.1360	4.2240
H	1.7860	1.1120	4.2570
C	1.8270	0.8610	-1.2480
H	2.6560	1.3180	-0.6850
O	0.7210	1.3850	-1.3350
C	-2.5780	1.9740	-1.1600
H	-1.7510	1.3320	-1.4690
H	-3.0100	1.5450	-0.2490
H	-3.3530	1.9590	-1.9310
C	1.2350	4.9080	-0.4950
H	1.5610	4.8400	0.5480
H	1.7040	4.0930	-1.0520
H	1.5860	5.8600	-0.8990
C	2.1120	-0.4010	-1.8950
C	1.3000	-1.3140	-2.5240
C	2.1540	-2.3790	-2.9010
H	0.2290	-1.2220	-2.6450
C	3.4130	-2.0280	-2.4750
H	1.8860	-3.2920	-3.4140
C	4.7040	-2.7390	-2.5400
O	5.7110	-2.3030	-2.0420
H	4.6700	-3.7070	-3.0720
O	3.3930	-0.8280	-1.8700
H	-0.1710	2.8110	-0.9300
H	3.4610	0.6210	2.8150
O	-1.4850	-1.8180	1.0310
C	-2.1630	-1.6620	-0.1170
O	-1.7530	-0.9910	-1.0440
C	-3.4560	-2.3920	-0.1210
C	-3.8950	-3.1150	0.9920

C -4.2350 -2.3420 -1.2800
C -5.1110 -3.7840 0.9410
H -3.2820 -3.1480 1.8860
C -5.4490 -3.0150 -1.3270
H -3.8700 -1.7780 -2.1330
C -5.8870 -3.7360 -0.2170
H -5.4550 -4.3480 1.8020
H -6.0530 -2.9840 -2.2270
H -6.8350 -4.2630 -0.2540

Hub^3-TEMPO

R(-1622.03694)	TS(-1622.015284)
C -2.7120 1.7410 -0.7030	C -1.9070 -0.6080 1.2850
C -3.6120 2.7220 -1.1160	C -1.6050 -2.1210 1.3560
C -4.7220 2.3490 -1.8630	C -0.9300 -2.1610 -1.1850
C -4.9050 1.0070 -2.1780	C -1.9180 -0.9820 -1.2460
C -3.9660 0.0770 -1.7370	C -1.6430 -0.0140 -0.1030
N -2.8950 0.4470 -1.0140	H -2.9530 -0.4590 1.5600
H -5.4390 3.0930 -2.1930	H -1.2830 -0.0760 2.0090
H -3.4380 3.7590 -0.8480	H -1.7750 -0.4650 -2.2020
H -5.7630 0.6780 -2.7540	H -2.9510 -1.3310 -1.2210
C 0.7550 -0.3070 -1.3150	H -0.5850 0.2400 -0.1540
C 0.8690 -1.7680 -1.8020	N -0.6350 -2.4680 0.2610
C 1.9910 -2.4420 0.5290	O 0.1270 -3.4550 0.4410
C 2.4840 -0.9940 0.4590	C 0.3910 -1.8260 -1.8800
C 1.3310 -0.0650 0.0870	H 1.1160 -2.6340 -1.7480
H 1.2850 0.3030 -2.0500	H 0.2040 -1.7110 -2.9510
H -0.2980 -0.0130 -1.3250	H 0.8240 -0.9060 -1.4870
H 2.8270 -0.7160 1.4600	C -1.5240 -3.4150 -1.8390
H 3.3230 -0.8920 -0.2280	H -1.7380 -3.1920 -2.8890
H 0.5350 -0.2480 0.8110	H -0.8100 -4.2410 -1.7950
N 0.9870 -2.6840 -0.6030	H -2.4560 -3.7240 -1.3620
O 0.3420 -3.6730 -0.5710	C -0.9880 -2.4930 2.7010
C 1.2560 -2.7660 1.8310	H -1.6840 -2.1850 3.4860
H 0.9540 -3.8150 1.8580	H -0.8300 -3.5700 2.7840
H 1.9510 -2.5740 2.6520	H -0.0420 -1.9700 2.8540
H 0.3870 -2.1230 1.9660	C -2.8830 -2.9580 1.1650
C 3.1250 -3.4520 0.2890	H -3.5070 -2.8340 2.0560
H 3.6510 -3.2740 -0.6520	H -3.4780 -2.6380 0.3080
H 3.8400 -3.3260 1.1070	H -2.6360 -4.0190 1.0650
H 2.7450 -4.4760 0.3140	C 1.8900 -1.8550 1.0490
C -0.3440 -2.2020 -2.6100	H 1.2970 -2.9630 0.7010
H -0.4880 -1.4610 -3.4010	H 2.1760 -2.1840 2.0660

H -0.1980 -3.1820 -3.0710	O 0.9200 -1.0040 0.9430
H -1.2340 -2.2280 -1.9770	C 3.0410 -1.6680 0.1230
C 2.1680 -1.9960 -2.6070	C 4.1230 -2.4170 -0.2540
H 2.0190 -1.4960 -3.5690	C 4.8700 -1.6070 -1.1560
H 3.0430 -1.5480 -2.1350	H 4.3520 -3.4210 0.0700
H 2.3370 -3.0590 -2.8000	C 4.1930 -0.4230 -1.2630
C -2.4320 -1.6610 1.4260	H 5.7900 -1.8650 -1.6620
H -2.2290 -2.6090 1.9350	C 4.4510 0.8110 -1.9920
H -3.4980 -1.6350 1.1700	O 3.7270 1.7840 -1.9260
O -1.6090 -1.6210 0.2700	H 5.3650 0.8060 -2.6120
H -1.9630 -0.8550 -0.2650	O 3.0770 -0.4590 -0.4840
C -1.4840 2.1000 0.0880	H 1.4010 0.7600 0.5300
H -1.0890 1.2240 0.6090	C 2.1740 2.0860 1.8710
H -0.6950 2.4880 -0.5670	C 2.5150 3.4070 2.0960
H -1.7010 2.8720 0.8300	C 2.2990 4.3510 1.0930
C -4.0960 -1.3850 -2.0710	C 1.7480 3.9650 -0.1210
H -3.6510 -2.0040 -1.2880	C 1.4070 2.6350 -0.3290
H -5.1430 -1.6710 -2.1990	N 1.6260 1.7620 0.6750
H -3.5760 -1.6080 -3.0100	H 2.5680 5.3890 1.2610
C -2.1150 -0.5100 2.3300	H 2.9510 3.6870 3.0480
C -2.8150 0.4900 2.9420	H 1.5820 4.6800 -0.9180
C -1.8470 1.3060 3.6030	C 2.3890 0.9770 2.8520
H -3.8860 0.6310 2.9170	H 2.7120 1.3800 3.8120
C -0.6280 0.7470 3.3360	H 1.4730 0.3960 2.9890
H -2.0300 2.1970 4.1870	H 3.1630 0.2960 2.4820
C 0.7590 1.0900 3.6380	C 0.7810 2.1280 -1.5860
O 1.6910 0.4300 3.2250	H 1.1390 1.1200 -1.8080
H 0.9080 1.9870 4.2620	H -0.3110 2.1230 -1.4840
O -0.7970 -0.3640 2.5740	H 1.0570 2.7750 -2.4200
O 1.6250 1.3160 0.2960	O -2.2540 1.2720 -0.2790
C 2.5920 1.9390 -0.3800	C -3.5790 1.4220 -0.3570
C 4.2730 2.0190 -1.8550	C -5.6830 0.6650 -0.3370
C 3.6580 3.8600 -0.7590	C -5.2780 2.8380 -0.6350
N 2.6940 3.2370 -0.0950	N -4.3840 0.3680 -0.2380
N 4.4870 3.3100 -1.6530	N -6.1960 1.8720 -0.5340
N 3.3430 1.2690 -1.2550	N -3.9630 2.6830 -0.5560
H 3.7850 4.9210 -0.5570	H -6.3800 -0.1640 -0.2460
H 4.9110 1.5150 -2.5780	H -5.6430 3.8490 -0.7980

P(-1622.056237)

C -2.2950 2.6860 -1.1180
C -3.6160 3.1110 -1.0600
C -4.0070 3.9960 -0.0620
C -3.0820 4.4620 0.8710
C -1.7690 4.0260 0.8000
N -1.4410 3.1590 -0.1830

H	-5.0370	4.3340	-0.0130
H	-4.3200	2.7450	-1.7990
H	-3.3680	5.1610	1.6480
C	-1.2050	-0.4520	0.8470
C	-0.1230	-0.4410	1.9560
C	1.3330	-2.1260	0.6930
C	-0.0130	-2.5220	0.0440
C	-0.7600	-1.2620	-0.3690
H	-2.1320	-0.8670	1.2500
H	-1.4140	0.5750	0.5220
H	0.1820	-3.1470	-0.8350
H	-0.6240	-3.1080	0.7320
H	-0.0810	-0.6450	-0.9670
N	1.1640	-0.7620	1.2820
O	2.2590	-0.4510	2.1060
C	2.4290	-2.0190	-0.3700
H	3.3550	-1.6570	0.0850
H	2.6180	-3.0040	-0.8080
H	2.1380	-1.3340	-1.1740
C	1.7680	-3.2040	1.6990
H	1.0850	-3.3090	2.5450
H	1.7960	-4.1690	1.1830
H	2.7800	-3.0170	2.0720
C	-0.0240	0.9790	2.5220
H	-1.0130	1.2850	2.8820
H	0.6840	1.0410	3.3510
H	0.2900	1.6750	1.7380
C	-0.5140	-1.3840	3.1080
H	-1.3640	-0.9560	3.6500
H	-0.8180	-2.3720	2.7560
H	0.3010	-1.4990	3.8320
C	2.1330	1.6740	-0.1000
H	2.3060	1.5650	0.9800
O	1.1250	2.2010	-0.5620
C	-1.7620	1.7360	-2.1440
H	-0.6700	1.7100	-2.1250
H	-2.1410	0.7220	-1.9650
H	-2.0950	2.0420	-3.1400
C	-0.6860	4.4460	1.7460
H	-0.7550	3.8740	2.6780
H	0.3040	4.2790	1.3140
H	-0.7900	5.5050	1.9920
C	3.1820	1.1680	-0.9600
C	3.3290	1.1360	-2.3250
C	4.5480	0.4570	-2.5630
H	2.6400	1.5480	-3.0480

C	5.0440	0.1290	-1.3240
H	5.0060	0.2310	-3.5150
C	6.2360	-0.6390	-0.9170
O	6.4490	-0.9440	0.2300
H	6.9070	-0.9260	-1.7470
O	4.2280	0.5680	-0.3530
H	-0.4580	2.8290	-0.2260
H	2.2700	-1.0890	2.8460
O	-1.8480	-1.4670	-1.2910
C	-2.9070	-2.2210	-0.9910
C	-4.0690	-3.6080	0.3230
C	-4.8940	-2.9890	-1.6530
N	-2.9610	-2.8820	0.1640
N	-5.0690	-3.7120	-0.5430
N	-3.8500	-2.2210	-1.9360
H	-4.1540	-4.1650	1.2530
H	-5.6860	-3.0300	-2.3970

Hub^7-TEMPO

R(-1739.666557)				TS(-1739.649003)			
C	2.0270	2.1110	0.4490	C	-0.0520	2.3060	0.8210
C	3.2830	2.6820	0.2200	C	-1.4220	3.0110	0.9750
C	3.4090	3.6710	-0.7430	C	-2.1180	2.2080	-1.4300
C	2.2830	4.0640	-1.4600	C	-0.5970	2.1790	-1.6490
C	1.0660	3.4460	-1.1930	C	0.0810	1.4970	-0.4720
N	0.9470	2.4850	-0.2550	H	0.7210	3.0820	0.8480
H	4.3730	4.1340	-0.9350	H	0.1110	1.6440	1.6760
H	4.1370	2.3420	0.7970	H	-0.3860	1.6180	-2.5620
H	2.3430	4.8360	-2.2200	H	-0.1890	3.1850	-1.7950
C	-1.4330	-0.2680	-1.9020	H	-0.4090	0.5320	-0.3290
C	-2.9160	0.0430	-2.2330	N	-2.4100	2.3500	0.0500
C	-3.6590	-1.6190	-0.2810	O	-3.6230	2.4490	0.3340
C	-2.2860	-2.2360	-0.5650	C	-2.7760	0.9100	-1.9000
C	-1.2360	-1.1340	-0.6490	H	-3.8660	0.9940	-1.8470
H	-1.0030	-0.7880	-2.7650	H	-2.4950	0.7250	-2.9410
H	-0.9050	0.6820	-1.7780	H	-2.4600	0.0700	-1.2790
H	-2.0420	-2.9060	0.2620	C	-2.7580	3.3860	-2.1740
H	-2.2880	-2.8380	-1.4800	H	-2.6310	3.2210	-3.2490
H	-1.3240	-0.4980	0.2400	H	-3.8270	3.4400	-1.9570
N	-3.7540	-0.2790	-1.0160	H	-2.2910	4.3400	-1.9240
O	-4.5550	0.5080	-0.6440	C	-1.9330	2.9180	2.4100
C	-3.8790	-1.3080	1.2010	H	-1.2320	3.4560	3.0540

H -4.8910 -0.9380 1.3760	H -2.9150 3.3860 2.5060
H -3.7380 -2.2390 1.7520	H -1.9880 1.8800 2.7380
H -3.1500 -0.5850 1.5680	C -1.3360 4.4990 0.5900
C -4.8200 -2.4820 -0.8060	H -0.8130 5.0330 1.3880
H -4.7140 -2.7500 -1.8580	H -0.7860 4.6680 -0.3370
H -4.8070 -3.4040 -0.2190	H -2.3390 4.9250 0.4970
H -5.7790 -1.9830 -0.6440	C -3.4350 0.1710 1.2870
C -3.1440 1.5020 -2.5950	H -3.9980 1.1670 0.7700
H -2.4950 1.7270 -3.4460	H -3.7680 0.3460 2.3280
H -4.1780 1.6910 -2.8950	O -2.1730 0.3520 1.0150
H -2.8730 2.1480 -1.7560	C -4.0600 -1.0440 0.6770
C -3.4510 -0.8790 -3.3510	C -5.3250 -1.5570 0.5940
H -2.9670 -0.5500 -4.2750	C -5.1960 -2.8380 -0.0140
H -3.1960 -1.9280 -3.1960	H -6.2330 -1.0780 0.9300
H -4.5320 -0.7700 -3.4760	C -3.8600 -3.0260 -0.2380
C -1.9680 2.5230 1.5190	H -5.9870 -3.5410 -0.2340
H -3.0520 2.5630 1.6790	C -3.0770 -4.1490 -0.7320
H -1.5870 3.5510 1.5650	O -1.8620 -4.1430 -0.7520
O -1.7610 1.9300 0.2530	H -3.6630 -5.0250 -1.0610
H -0.7880 2.0190 0.0610	O -3.1660 -1.9240 0.1670
C 1.8600 1.0540 1.5050	H -1.1250 -1.4070 0.3420
H 0.8800 0.5780 1.4240	C 0.3550 -1.8050 1.6420
H 2.6320 0.2830 1.4040	C 1.6850 -2.1420 1.7910
H 1.9500 1.4940 2.5040	C 2.4500 -2.4270 0.6560
C -0.1810 3.8030 -1.9570	C 1.8630 -2.4050 -0.5970
H -1.0360 3.8940 -1.2820	C 0.5220 -2.0580 -0.7340
H -0.0560 4.7400 -2.5020	N -0.1660 -1.7750 0.3890
H -0.4140 3.0200 -2.6880	H 3.5070 -2.6600 0.7490
C -1.3200 1.7100 2.6000	H 2.1190 -2.1670 2.7840
C -0.7060 1.9710 3.7930	H 2.4400 -2.6200 -1.4880
C -0.3260 0.7050 4.3290	C -0.5650 -1.4410 2.7690
H -0.5420 2.9450 4.2310	H -1.5870 -1.7460 2.5330
C -0.7310 -0.2340 3.4220	H -0.2540 -1.9400 3.6880
H 0.1900 0.5150 5.2600	H -0.5740 -0.3600 2.9450
C -0.6280 -1.6860 3.3130	C -0.1390 -1.9440 -2.0700
O -1.0690 -2.2920 2.3580	H -0.1410 -2.9240 -2.5580
H -0.1270 -2.1990 4.1510	H -1.1810 -1.6330 -1.9920
O -1.3440 0.3820 2.3760	H 0.4260 -1.2270 -2.6760
C 1.2690 -1.0290 -0.8300	C 2.5620 1.2170 0.0700
C 2.2410 -1.9870 -0.6370	C 3.5870 0.6180 -0.6510
N 0.1040 -1.6960 -0.6610	N 1.4980 1.1950 -0.7650
N 0.3290 -2.9900 -0.3940	N 1.8320 0.6190 -1.9350
N 1.6070 -3.1690 -0.3810	N 3.0780 0.2920 -1.8760
H 1.3040 0.0300 -1.0420	H 2.5150 1.6100 1.0730
C 3.7020 -1.8490 -0.6330	C 4.9630 0.3030 -0.2140
C 4.5070 -2.9440 -0.3040	C 5.7810 -0.5360 -0.9830

C 4.3020 -0.6210 -0.9290	C 5.4430 0.8010 1.0020
C 5.8900 -2.8060 -0.2710	C 7.0510 -0.8730 -0.5270
H 4.0350 -3.8940 -0.0760	H 5.4170 -0.9130 -1.9350
C 5.6850 -0.4860 -0.8920	C 6.7130 0.4580 1.4530
H 3.6870 0.2340 -1.2020	H 4.8270 1.4770 1.5900
C 6.4840 -1.5790 -0.5620	C 7.5200 -0.3820 0.6910
H 6.5080 -3.6610 -0.0160	H 7.6810 -1.5200 -1.1280
H 6.1410 0.4710 -1.1280	H 7.0740 0.8540 2.3970
H 7.5640 -1.4760 -0.5350	H 8.5130 -0.6480 1.0380

P(-1739.695635)

C -1.6130 3.1510 -1.0290
C -2.5670 4.1560 -0.9320
C -2.1650 5.4600 -0.6640
C -0.8150 5.7620 -0.4980
C 0.1250 4.7490 -0.5980
N -0.3180 3.4970 -0.8530
H -2.9070 6.2490 -0.5910
H -3.6120 3.9050 -1.0740
H -0.4840 6.7750 -0.3000
C 0.0700 0.5090 1.5480
C 1.3830 0.8090 2.3200
C 2.1920 -1.5980 2.0210
C 0.6860 -1.8890 1.8290
C 0.1200 -0.8570 0.8590
H -0.7790 0.5340 2.2420
H -0.1070 1.2790 0.7900
H 0.5620 -2.9040 1.4360
H 0.1460 -1.8400 2.7830
H 0.7660 -0.8030 -0.0190
N 2.3950 -0.1360 1.7790
O 3.6920 0.2400 2.1580
C 2.9920 -2.3370 0.9410
H 4.0050 -1.9300 0.8730
H 3.0540 -3.4020 1.1850
H 2.5060 -2.2440 -0.0340
C 2.6800 -2.0940 3.3910
H 2.2430 -1.5560 4.2340
H 2.4070 -3.1480 3.4990
H 3.7720 -2.0430 3.4610
C 1.8440 2.2320 1.9880
H 1.0320 2.9310 2.2220
H 2.7260 2.5150 2.5650

H	2.0830	2.3140	0.9240
C	1.1570	0.7370	3.8410
H	0.5310	1.5790	4.1540
H	0.6520	-0.1800	4.1540
H	2.1010	0.8210	4.3920
C	2.5430	0.8230	-1.1780
H	3.3890	1.2290	-0.6000
O	1.4680	1.4080	-1.2640
C	-1.9260	1.7140	-1.3030
H	-1.0350	1.1510	-1.5900
H	-2.3530	1.2450	-0.4070
H	-2.6700	1.6270	-2.0980
C	1.6000	4.9400	-0.4290
H	1.8680	4.8870	0.6310
H	2.1600	4.1670	-0.9610
H	1.9010	5.9190	-0.8070
C	2.7700	-0.4450	-1.8320
C	1.9350	-1.2840	-2.5330
C	2.7270	-2.4090	-2.8680
H	0.8840	-1.1180	-2.7280
C	3.9750	-2.1670	-2.3450
H	2.4240	-3.2910	-3.4150
C	5.2070	-2.9800	-2.3290
O	6.2050	-2.6340	-1.7500
H	5.1350	-3.9360	-2.8800
O	4.0080	-0.9770	-1.7230
H	0.3950	2.7510	-0.9400
H	3.7770	0.0830	3.1180
C	-2.3720	-1.3840	0.9070
C	-3.2630	-1.5850	-0.1310
N	-1.1780	-1.2050	0.3000
N	-1.3090	-1.2870	-1.0290
N	-2.5560	-1.5120	-1.2950
H	-2.4840	-1.3780	1.9810
C	-4.7100	-1.8410	-0.1050
C	-5.3810	-2.1540	-1.2910
C	-5.4280	-1.7750	1.0920
C	-6.7500	-2.3990	-1.2740
H	-4.8180	-2.2090	-2.2170
C	-6.7960	-2.0220	1.1060
H	-4.9190	-1.5290	2.0200
C	-7.4610	-2.3350	-0.0770
H	-7.2630	-2.6450	-2.1980
H	-7.3430	-1.9720	2.0420
H	-8.5280	-2.5300	-0.0660

10. References

[1] H. G. Cha, K-S. Choi, *Nat. Chem.* **2015**, *7*, 328–333.

[2] B. You, N. Jiang, X. Liu, Y. Sun, *Angew. Chem. Int. Ed.* **2016**, *55*, 9913–9917.

[3] N. Jiang, B. You, R. Boonstra, I. M. T. Rodriguez, Y. Sun, *ACS Energy Lett.* **2016**, *1*, 386–390.

[4] L. Gao, Y. Bao, S. Gan, Z. Sun, Z. Song, D. Han, F. Li, L. Niu, *ChemSusChem* **2018**, *11*, 2547–2553.

[5] A. C. Cardiel, B. J. Taitt, K-S. Cho, *ACS Sustainable Chem. Eng.* **2019**, *7*, 11138–11149.

[6] X. H. Chadderdon, D. J. Chadderdon, T. Pfennig, B. H. Shanks, W. Li, *Green Chem.* **2019**, *21*, 6210–6219.

[7] S. Nakatsuji, T. Ojima, H. Akutsu, J-i. Yamada, *J. Org. Chem.* **2002**, *67*, 916–921.

[8] N. Mibu, K. Yokomizo, A. Yuzuriha, M. Otsubo, Y. Kawaguchi, M. Sano, I. Sakai, K. Nakayama, J-R. Zhou, K. Sumoto, *Heterocycles* **2017**, *94*, 1653–1677.

[9] J. Wang, Y. Xia, H. Liu, J. Xia, M. Qian, L. Zhang, L. Chen, Q. Che, *J. Colloid Interface Sci.* **2019**, *551*, 1–9.

[10] M. Kavala, V. Brezova, Ľ. Švorc, Z. Vihonska, P. Olejnikova, J. Moncol, J. Kožíšek, P. Herich, P. Szolcsanyi, *Org. Biomol. Chem.* **2014**, *12*, 4491–4502.

[11] a) T. Kalai, R.Altman, I. Maezawa, M. Balog, C. Morisseau, J. Petrlova, B. D. Hammock, L-W. Jin, J. R.Trudell, J. C.Voss, K. Hideg, *Eur. J. Med Chem.* **2014**, *77*, 343–350; b) K. Hideg, H. O. Hankovszky, J. Tigyi, *Acta Chim. Acad. Sci. Hun.* **1977**, *92*, 85–87.

[12] C. Cougnon, S. Boisard, O. Cador, M. Dias, E. Levillaina, T. Breton, *Chem. Commun.* **2013**, *49*, 4555–4557.

[13] Source: https://www.ika.com/en/Products-Lab-Eq/Electrochemistry-Kit-csp-516/ElectraSyn-20-Package-Accessories-cpacc-20008980/ (12.11.2019.)

[14] T. Fodi, C. Didaskalou, J. Kupai, G. T. Balogh, P. Huszthy, G. Szekely, *ChemSusChem* **2017**, *10*, 3435–3444.

[15] S. R. McCabe, P. Wipf, *Angew. Chem. Int. Ed.* **2016**, *56*, 324–327.
